# Supplementary material for: Cooperative redox activation for carbon dioxide conversion
Source: Nat Commun. 2016 Dec 16;7:13782. doi: 10.1038/ncomms13782 (PMC5171923; doi:10.1038/ncomms13782)
Supplement: Supplementary Information — Supplementary Figures, Supplementary Tables, Supplementary Note and Supplementary Methods. [file ncomms13782-s1.pdf]

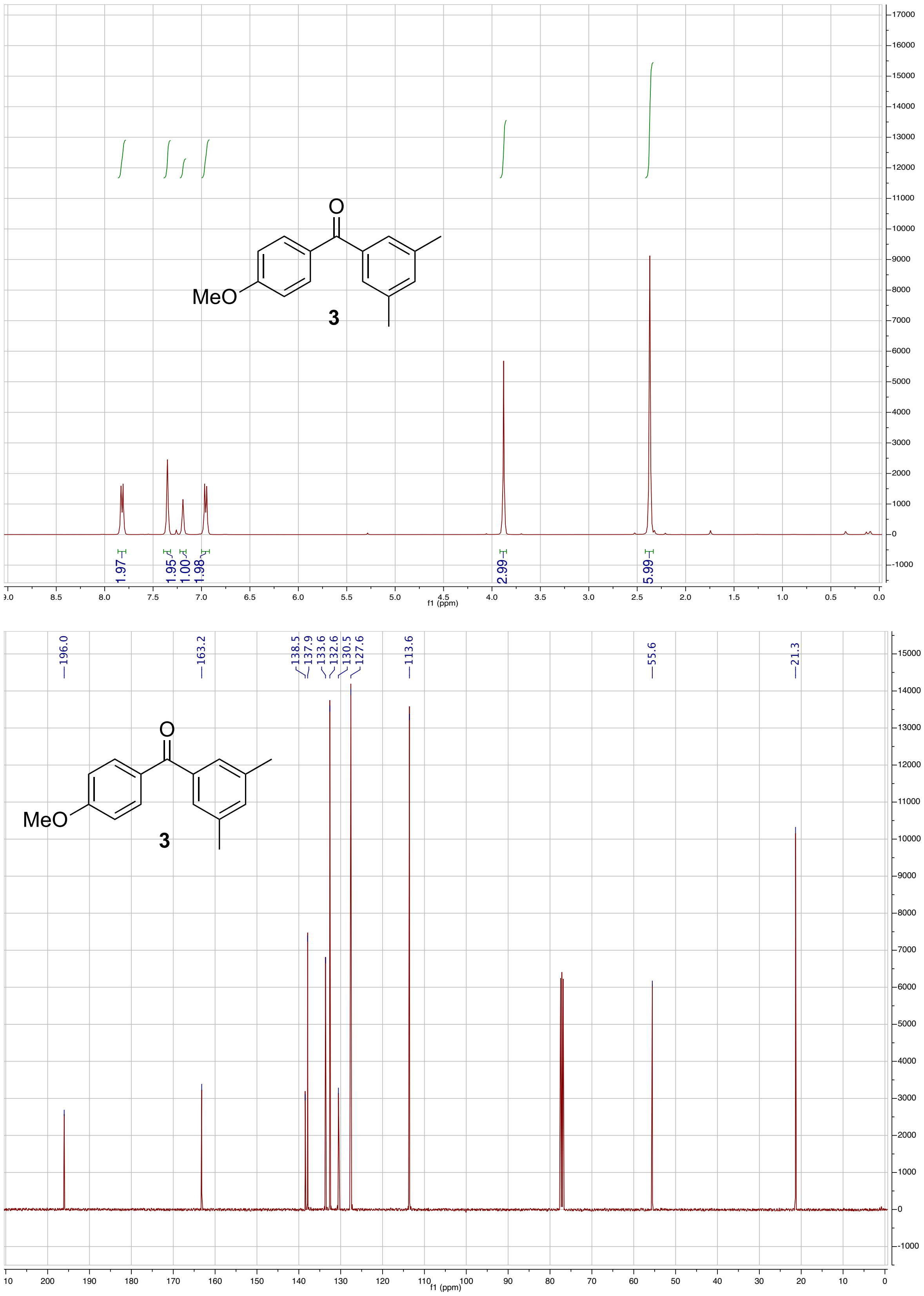

Supplementary Figure 1: ^{1}H and ^{13}C NMR of compound **3**.

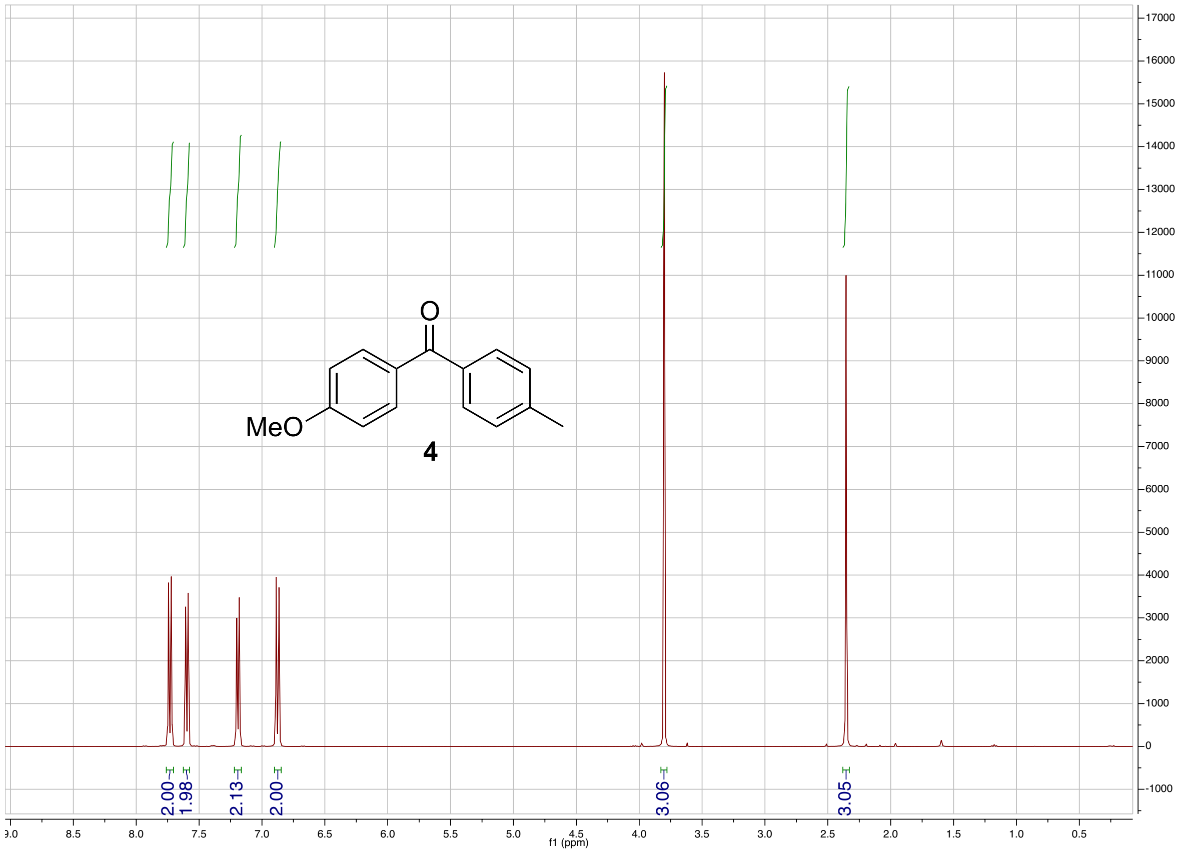

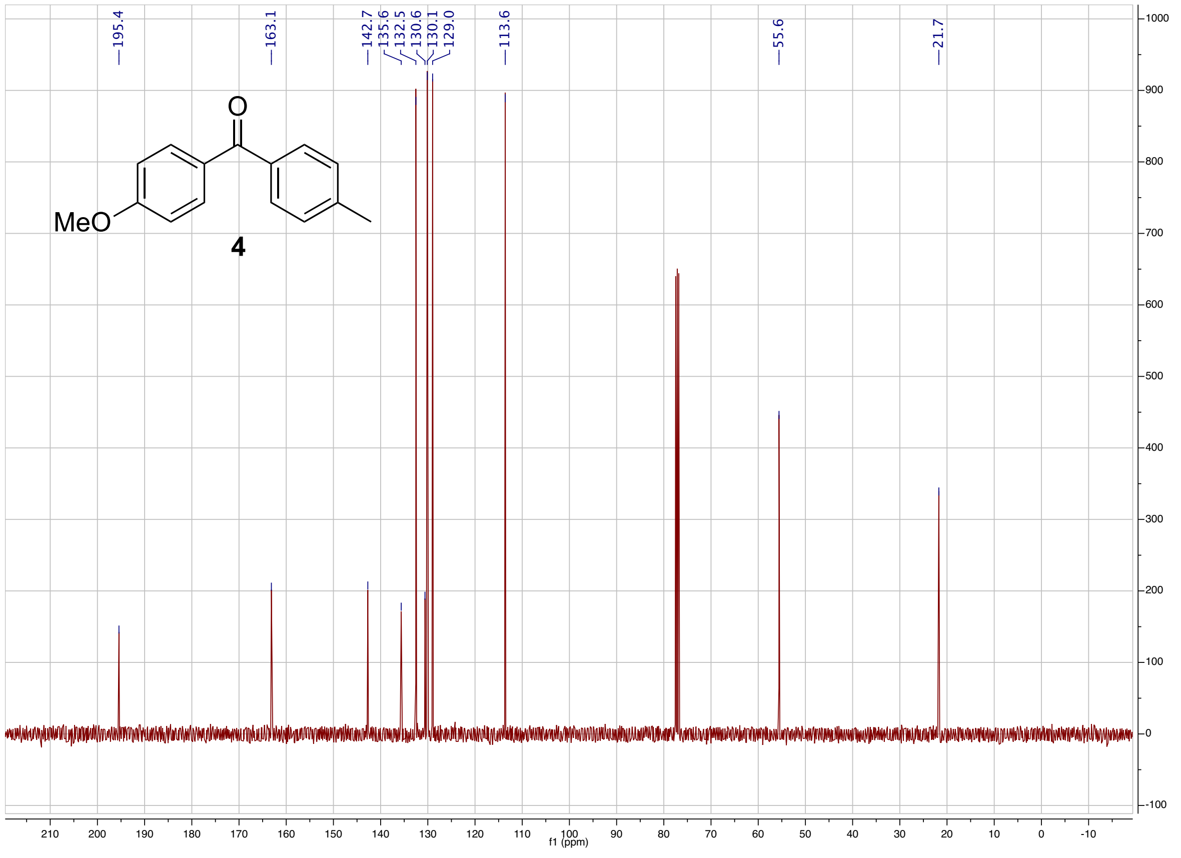

Supplementary Figure 2: ^{1}H and ^{13}C NMR of compound **4.**

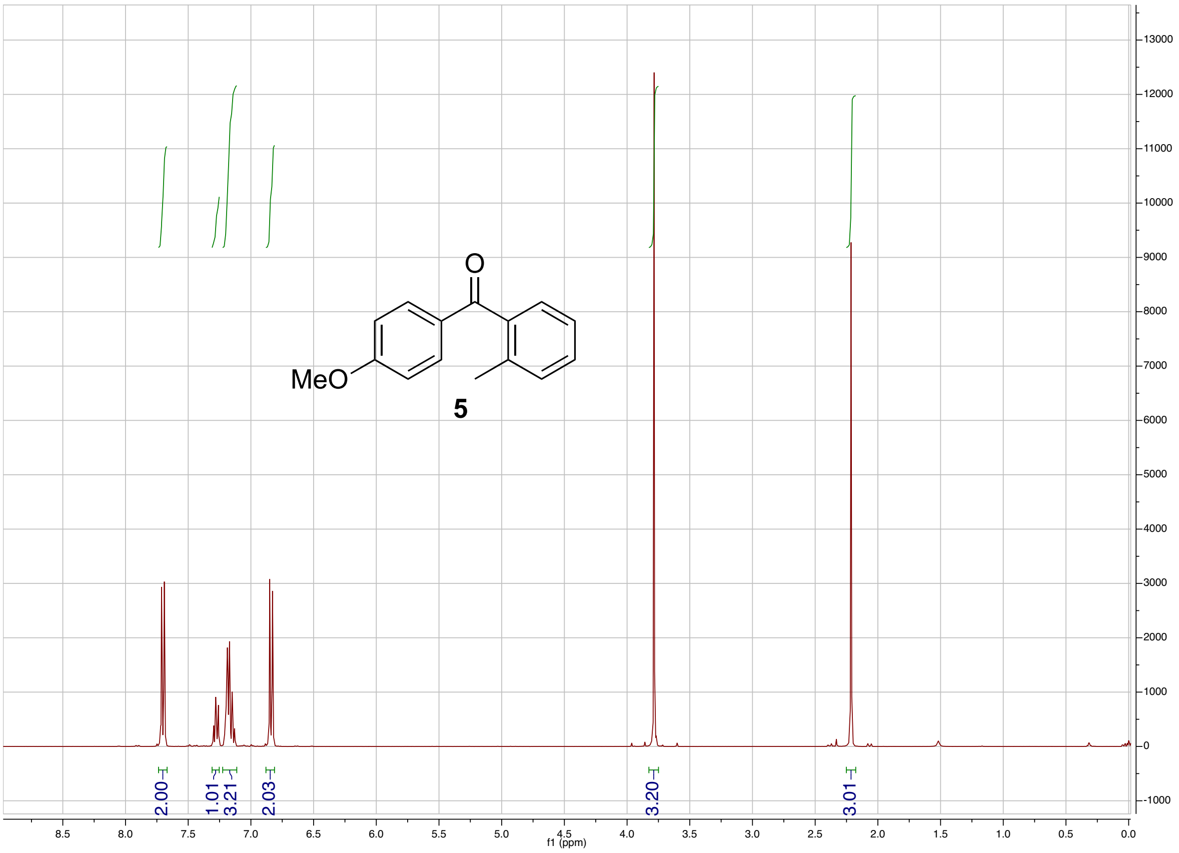

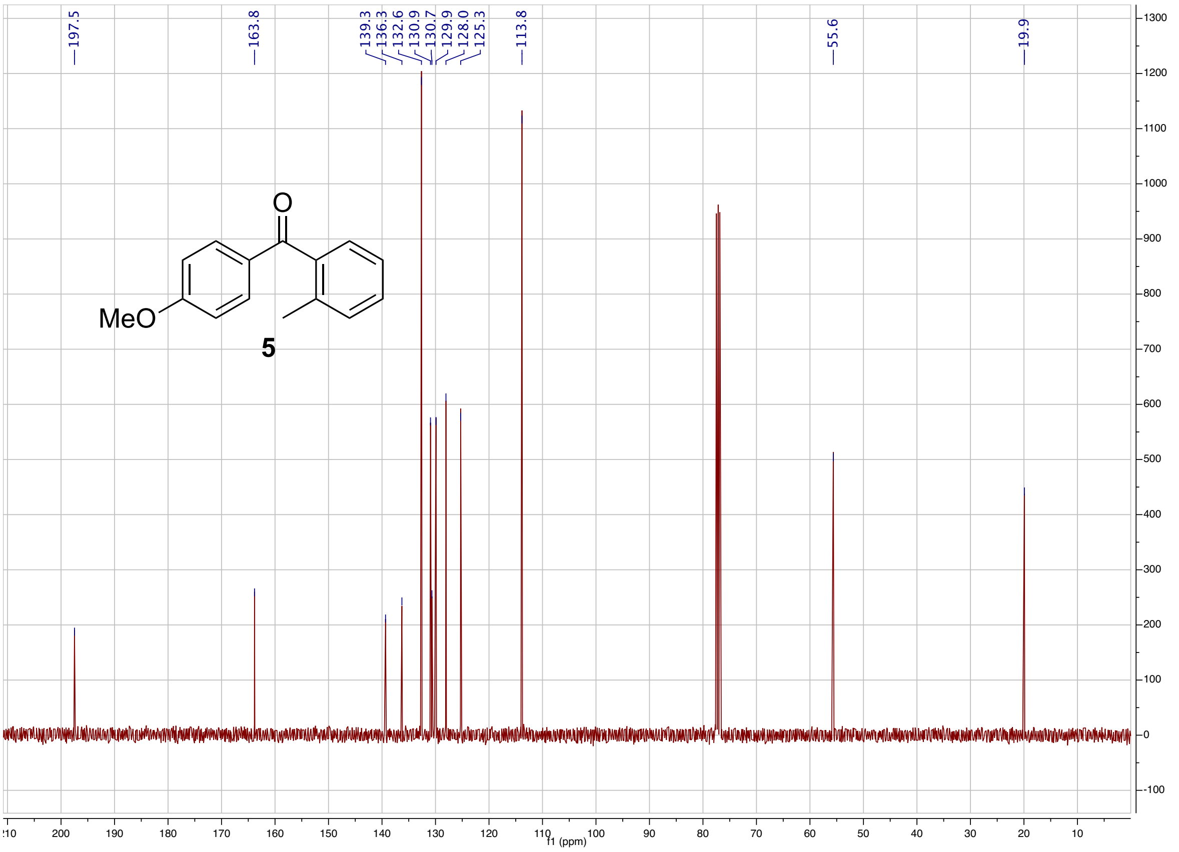

Supplementary Figure 3: ¹H and ¹³C NMR of compound **5**.

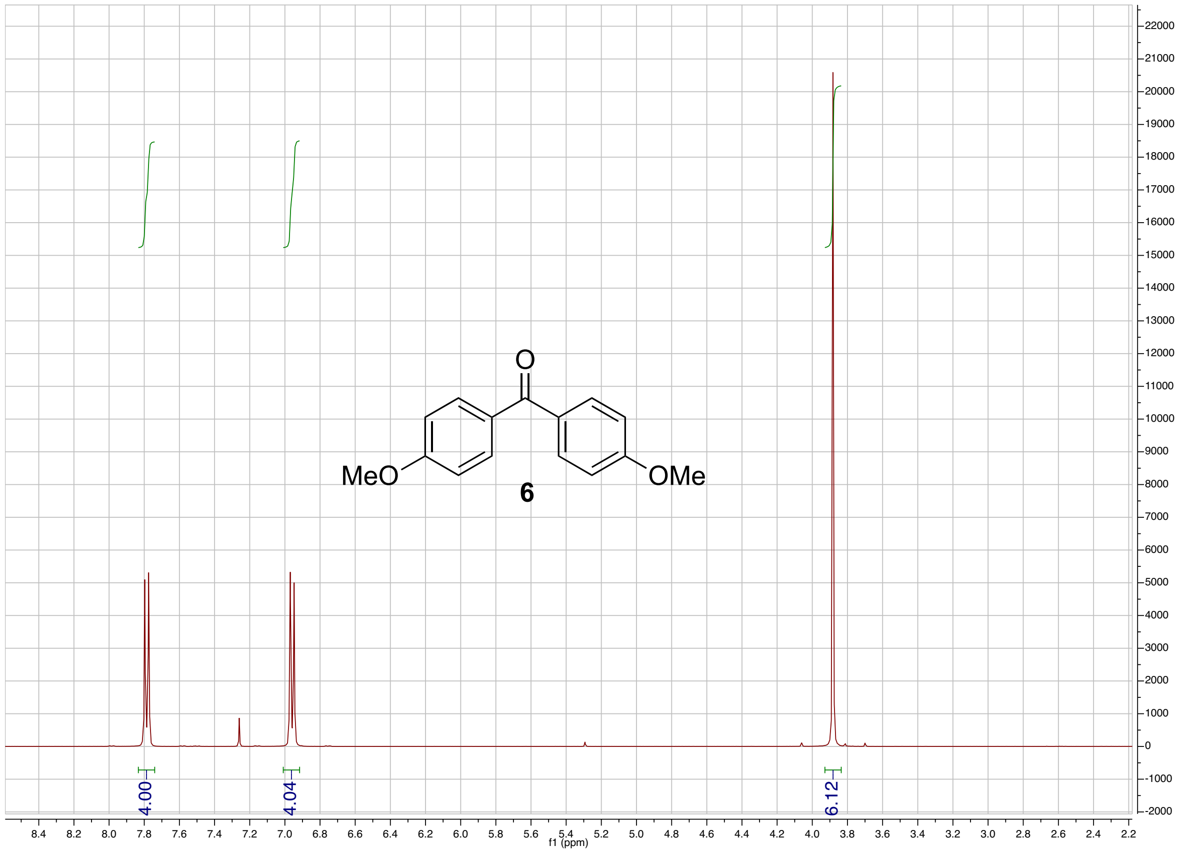

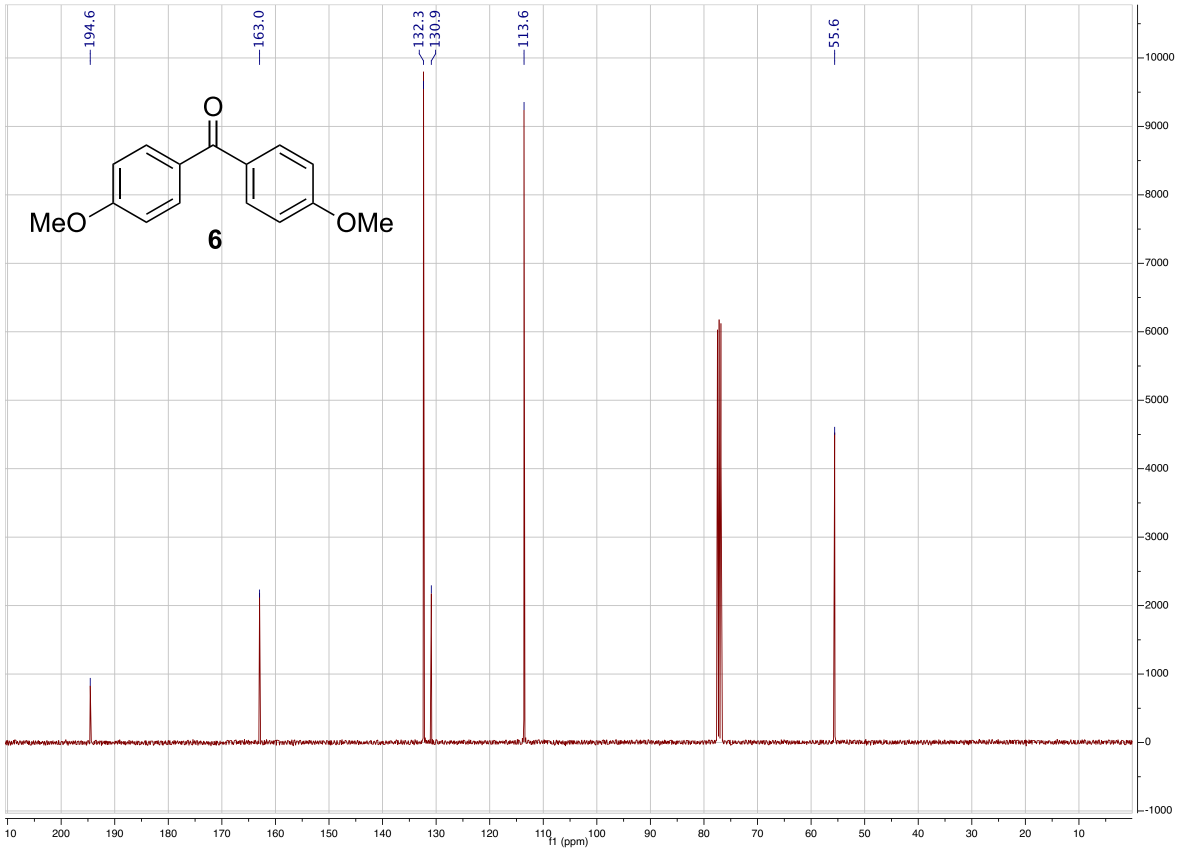

Supplementary Figure 4: ^{1}H and ^{13}C NMR of compound **6.**

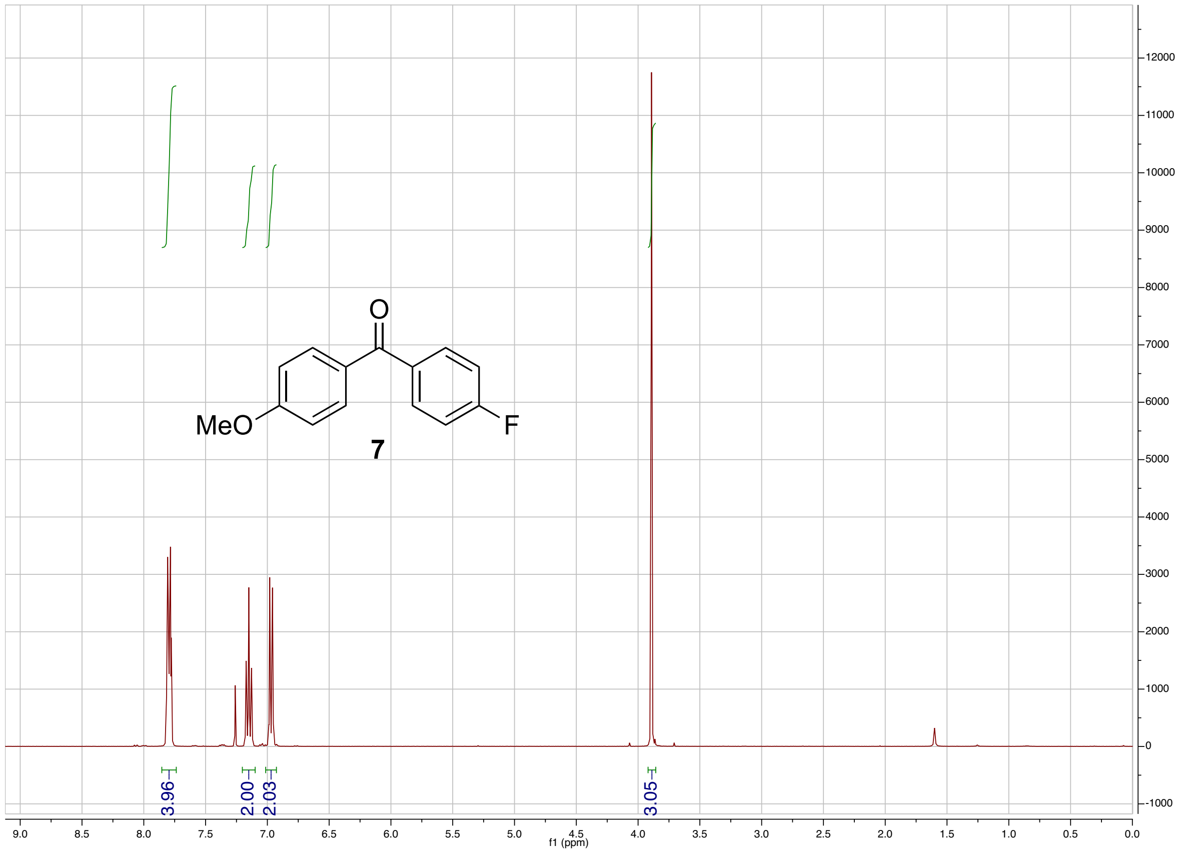
MeO
F
7
3.96
2.00
2.03
3.05
f1 (ppm)

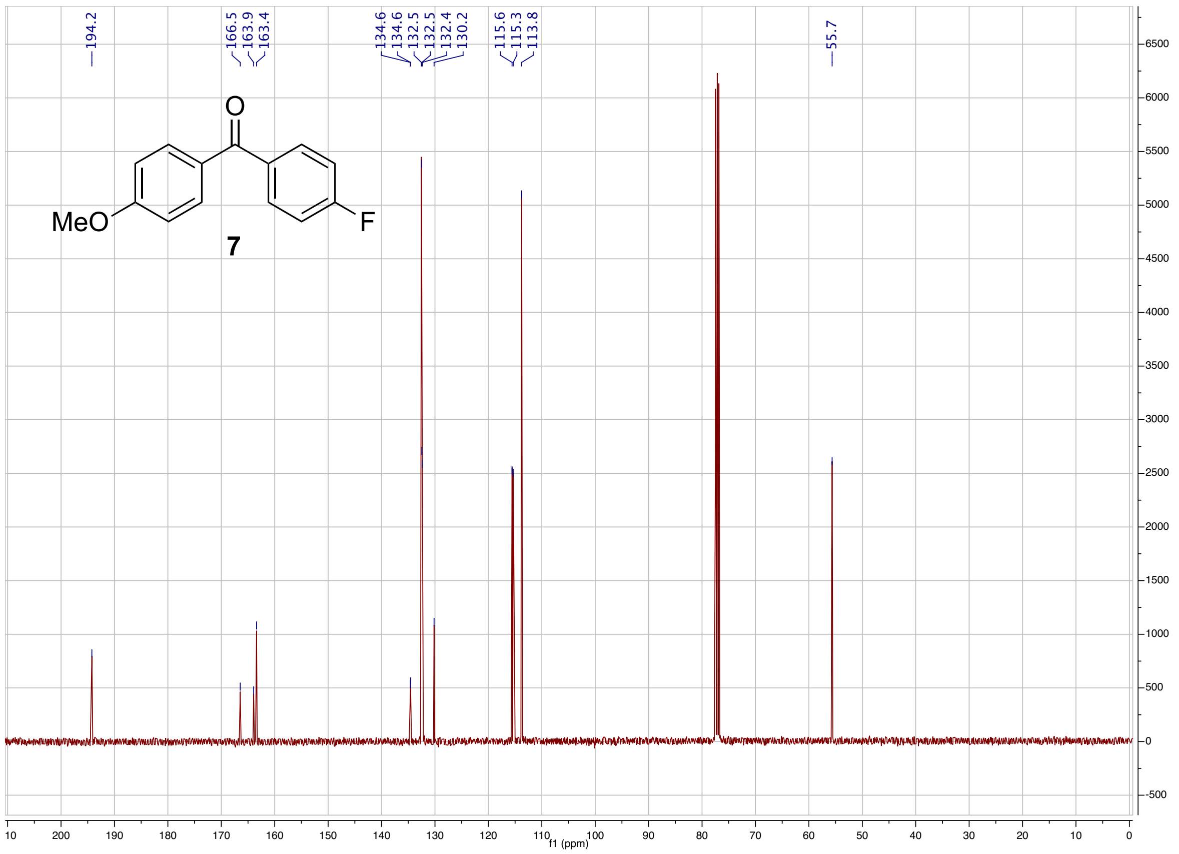
194.2
166.5
163.9
163.4
134.6
134.6
132.5
132.5
132.4
130.2
115.6
115.3
113.8
55.7
MeO
F
7
f1 (ppm)

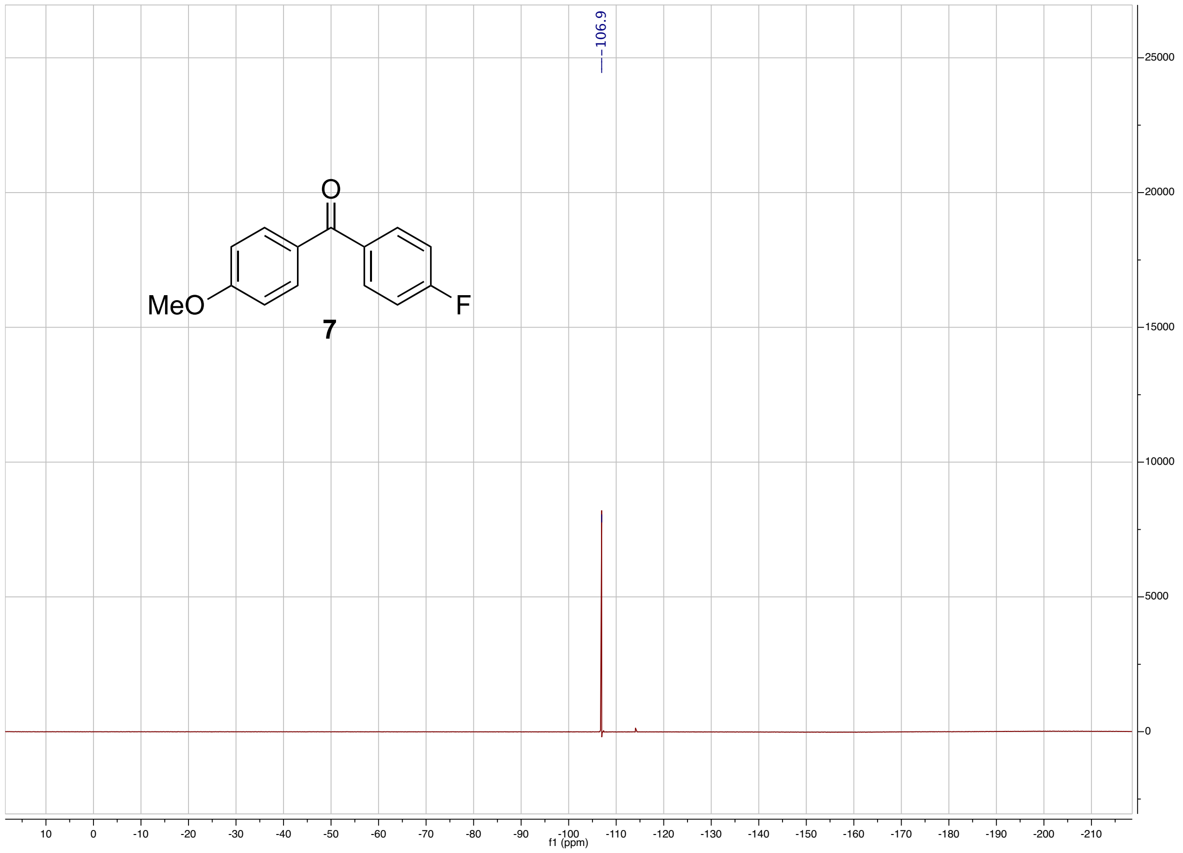

Supplementary Figure 5: ^{1}H, ^{13}C and ^{19}F NMR of compound **7**.

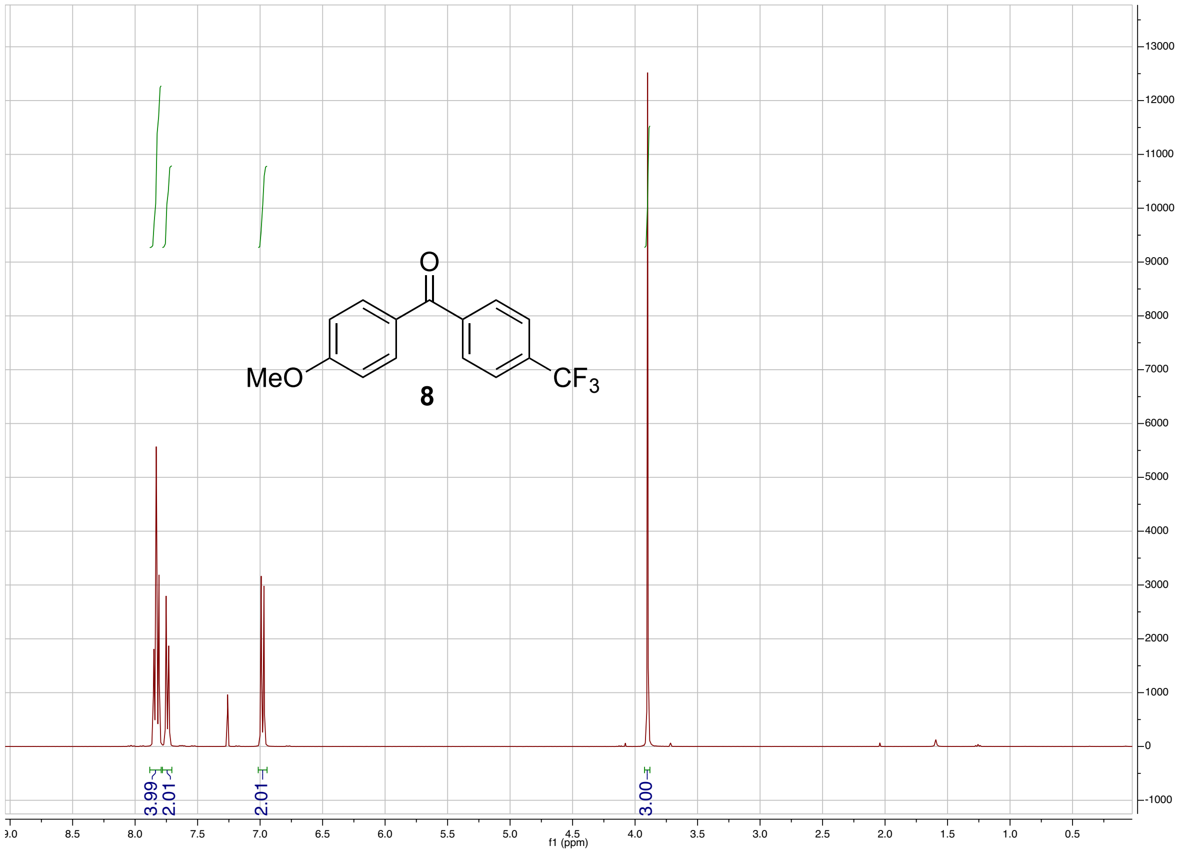

MeO
CF3
8
3.99
2.01
2.01
3.00
f1 (ppm)

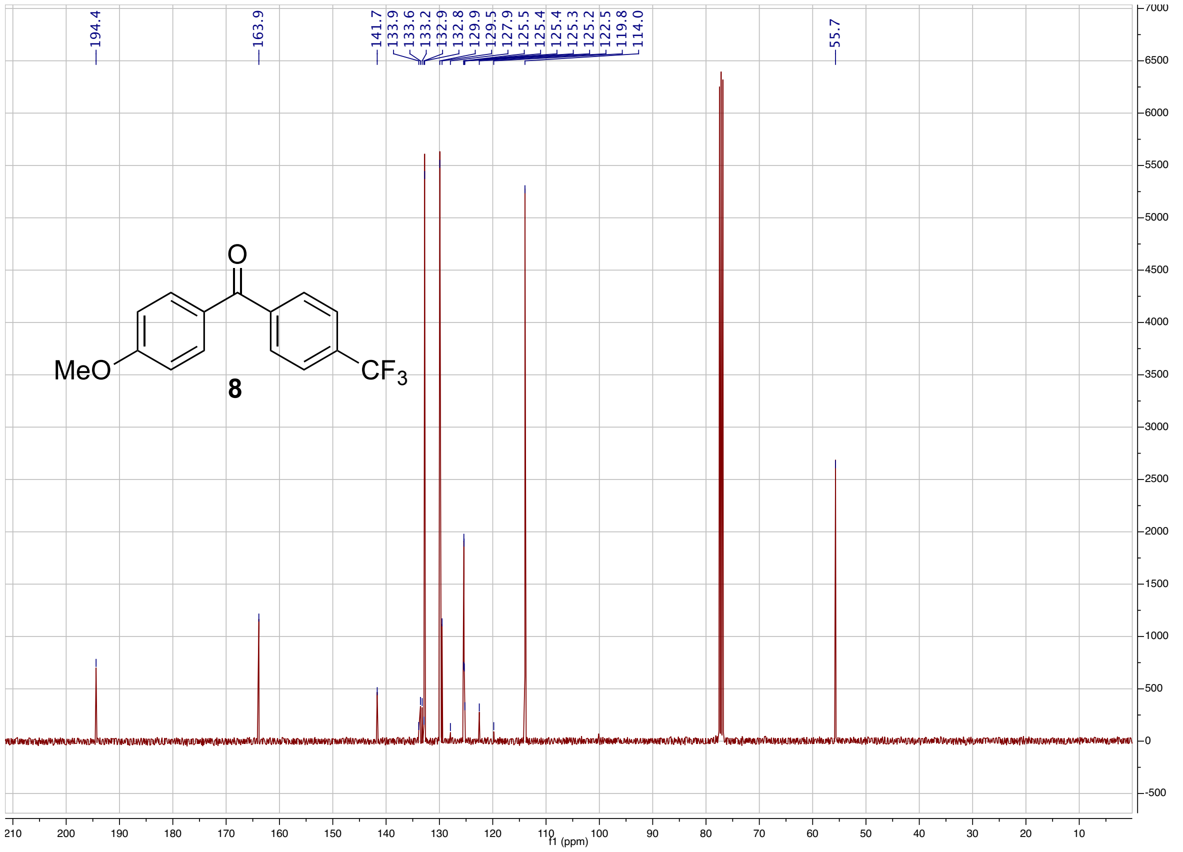

194.4
163.9
141.7
133.9
133.6
133.2
132.9
132.8
129.9
129.5
127.9
125.5
125.4
125.3
125.2
122.5
119.8
114.0
55.7
MeO
CF3
8
f1 (ppm)

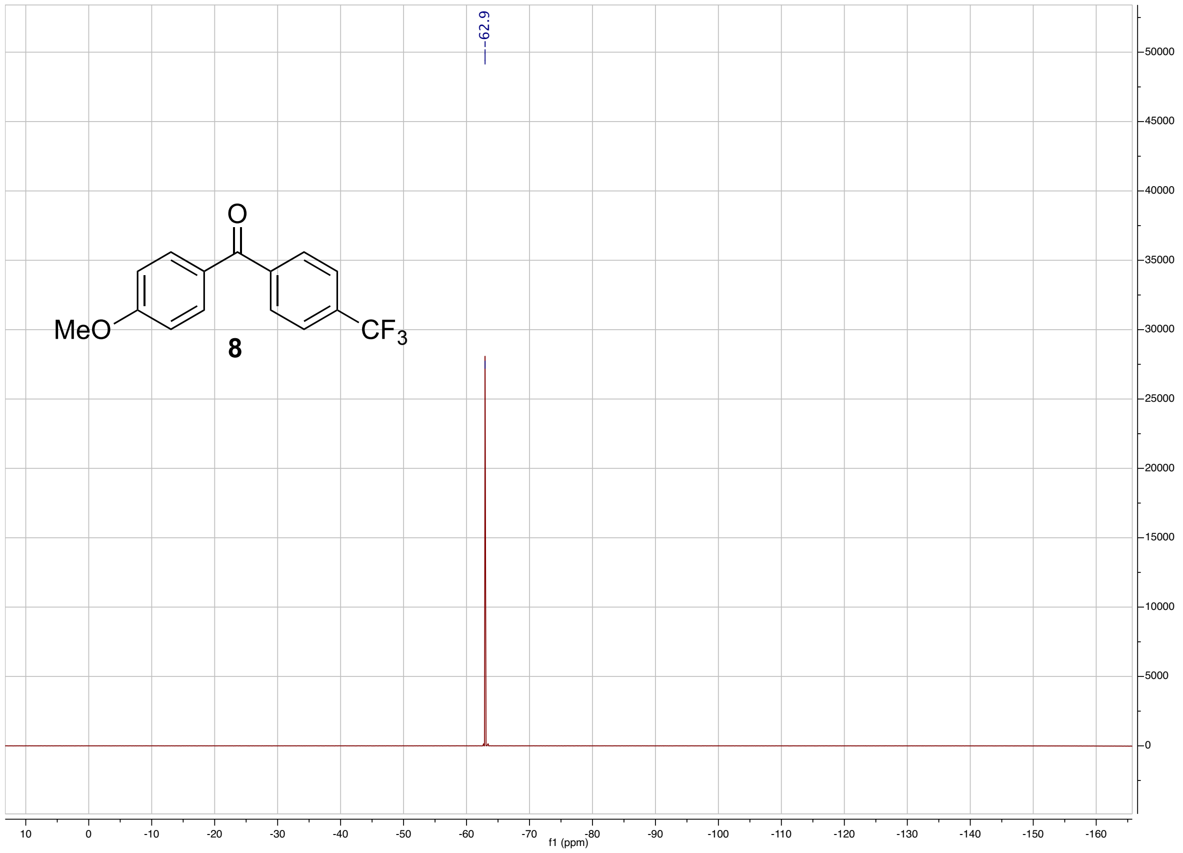

Supplementary Figure 6: ^{1}H, ^{13}C and ^{19}F NMR of compound **8**.

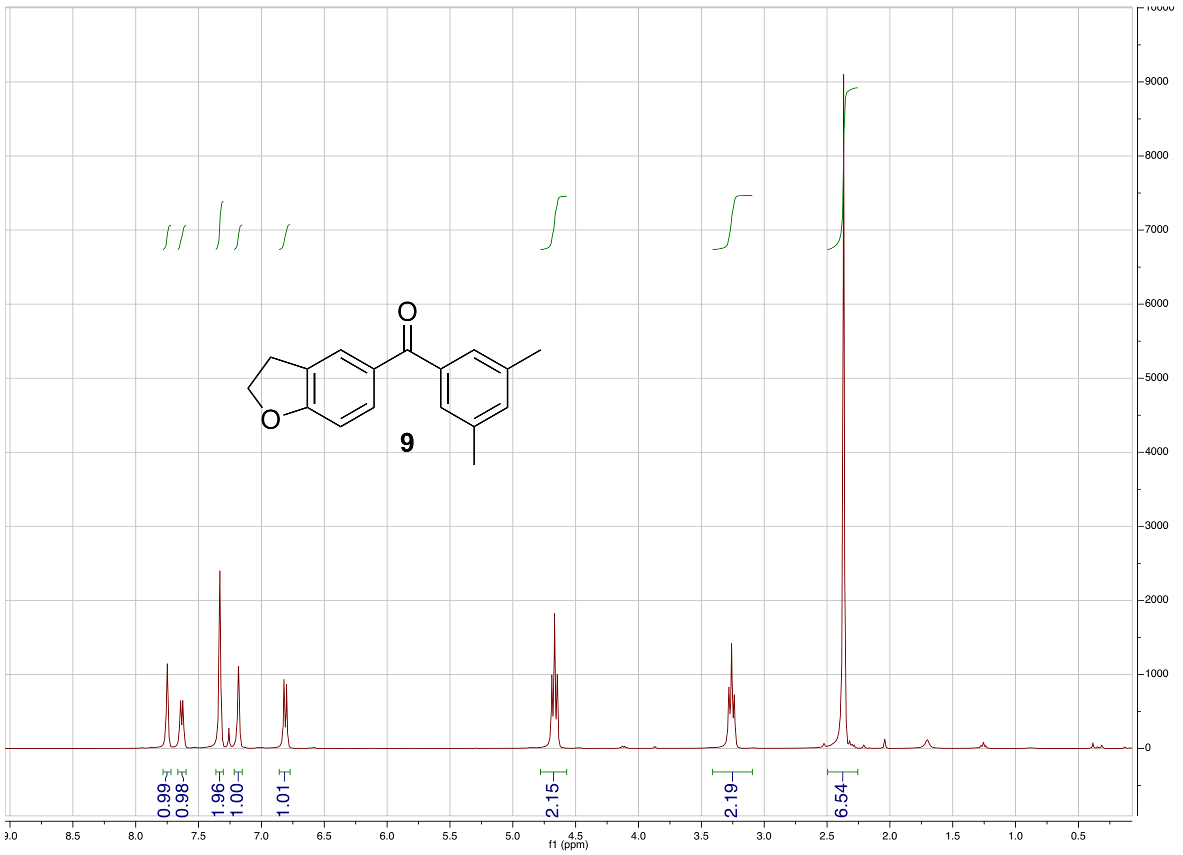

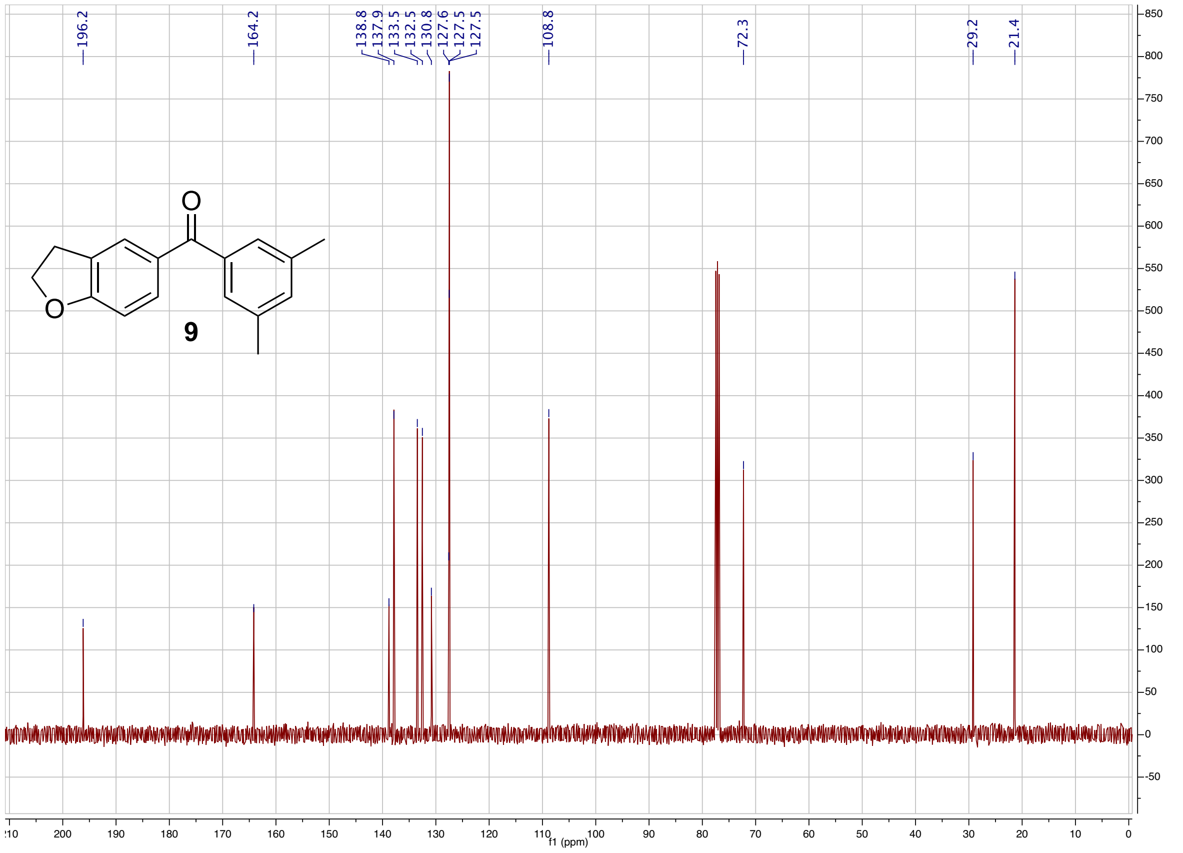

Supplementary Figure 7: ^{1}H and ^{13}C NMR of compound **9**.

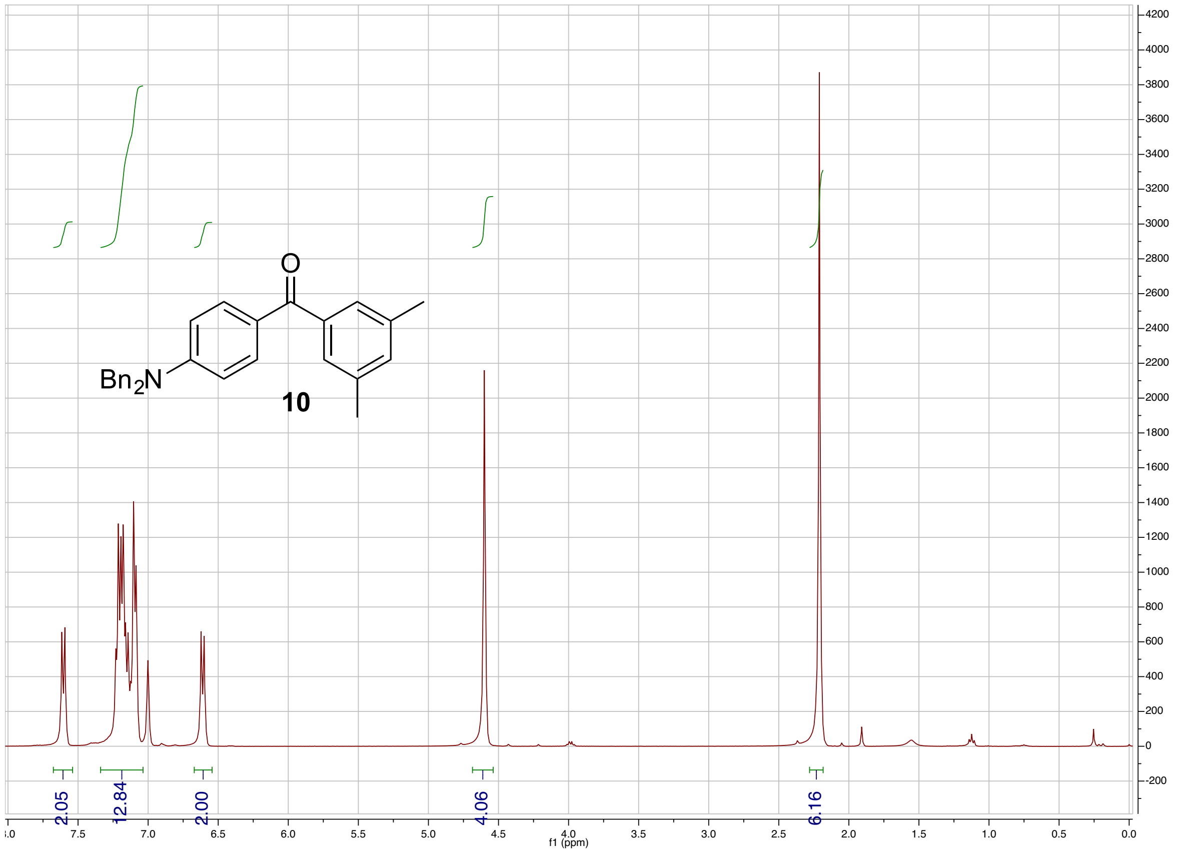

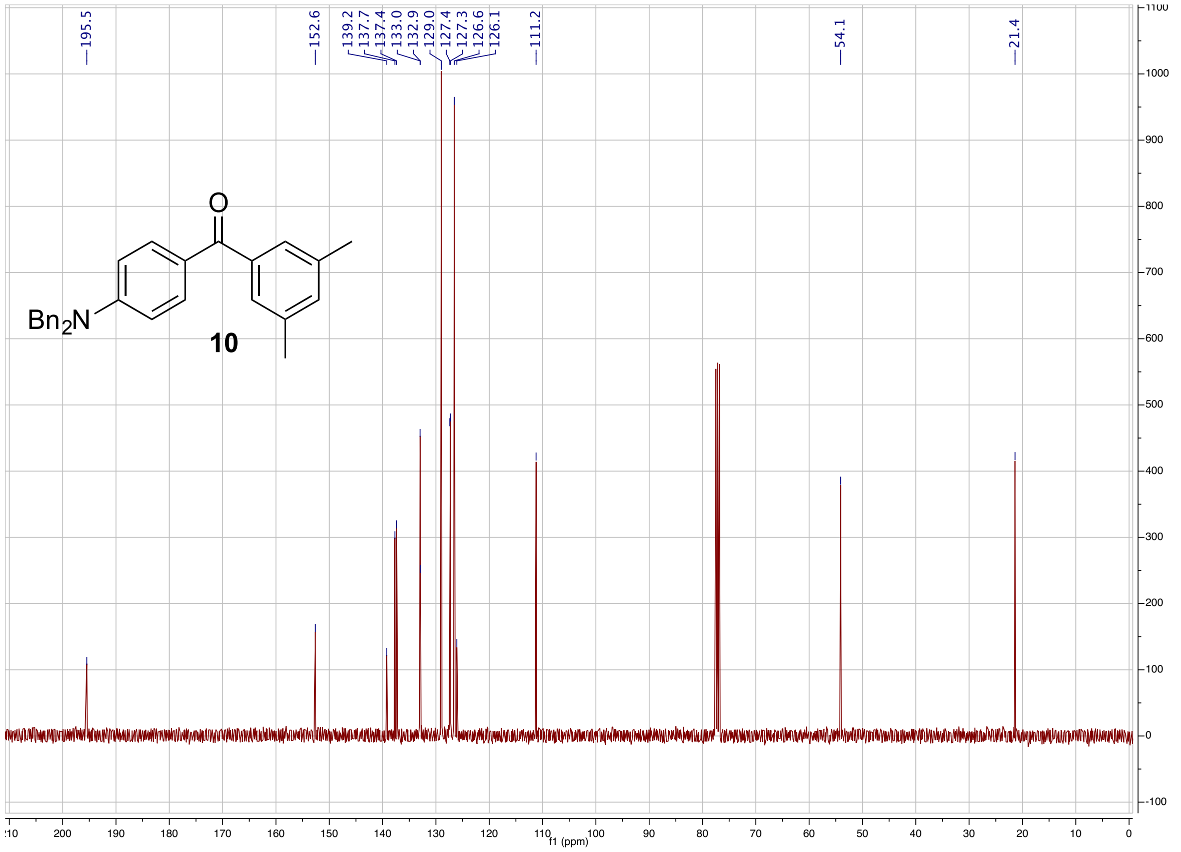

Supplementary Figure 8: ^{1}H and ^{13}C NMR of compound **10**.

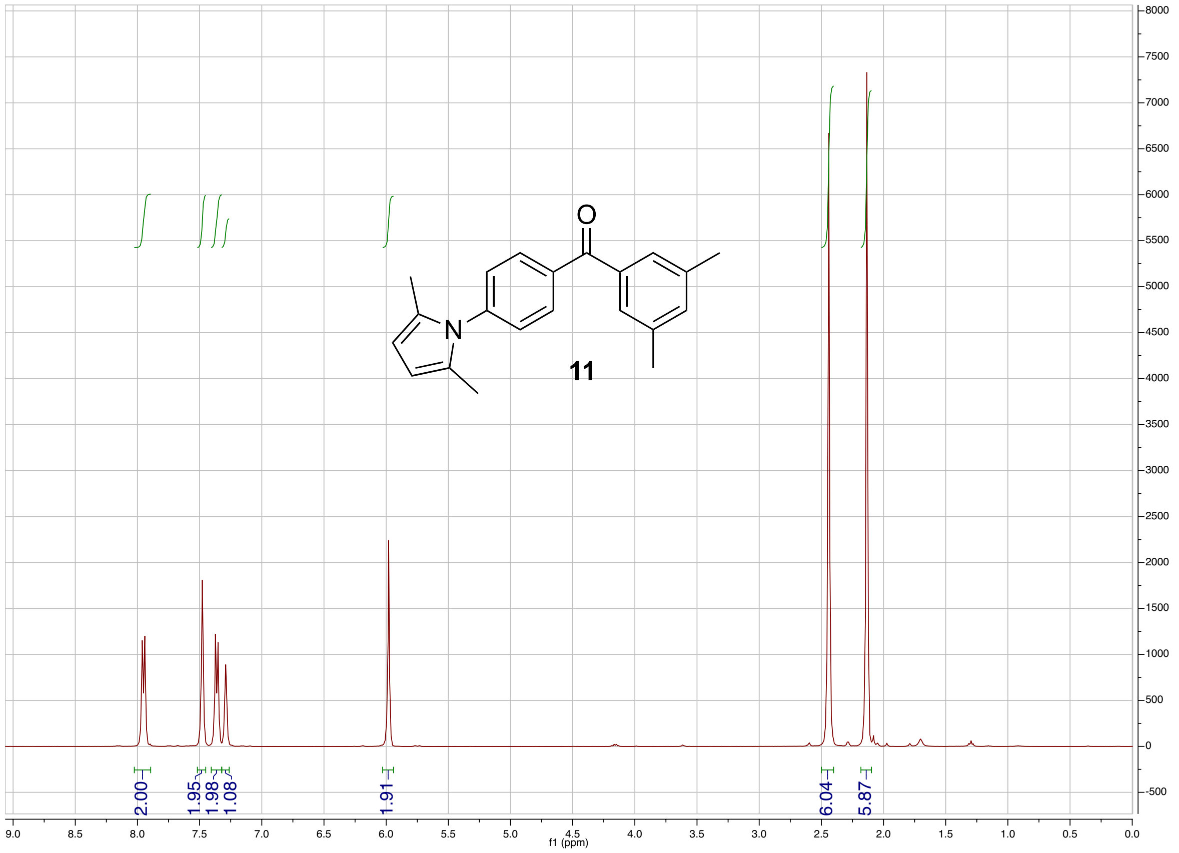

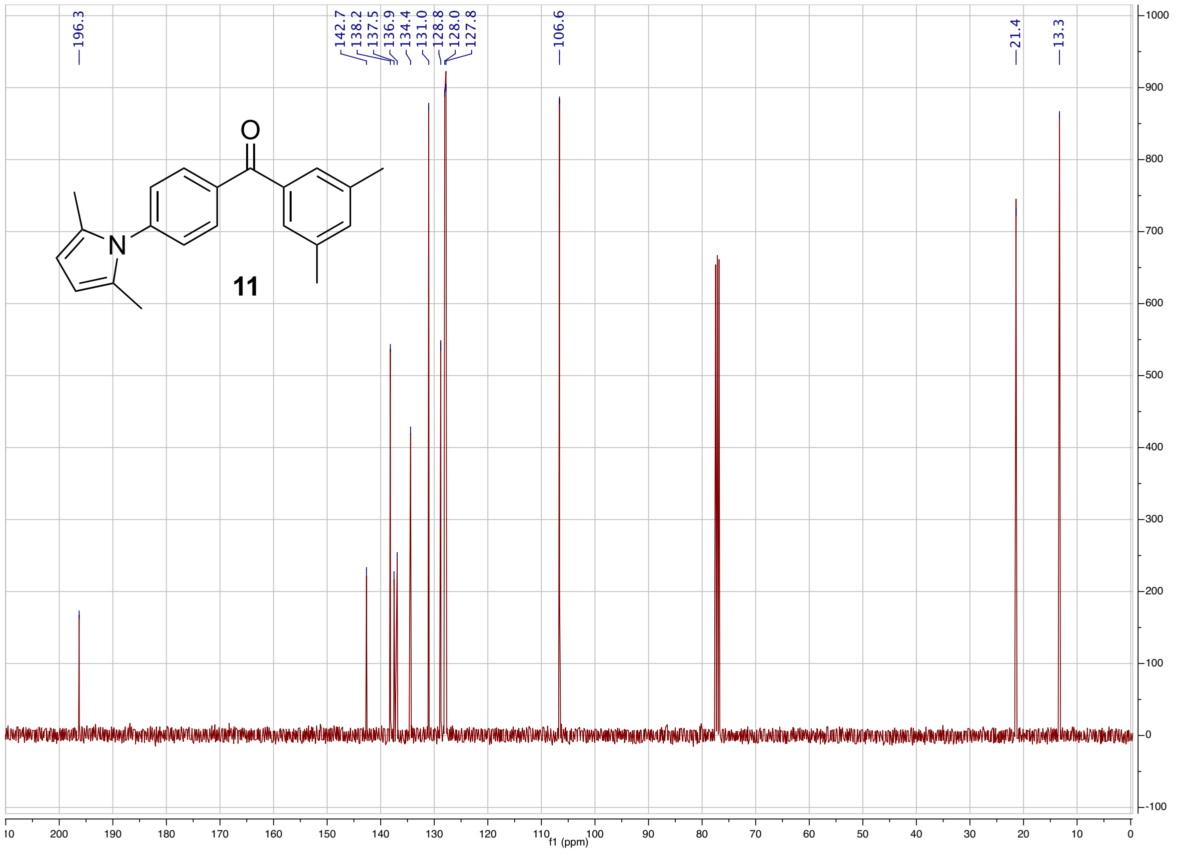

Supplementary Figure 9: ^{1}H and ^{13}C NMR of compound **11**.

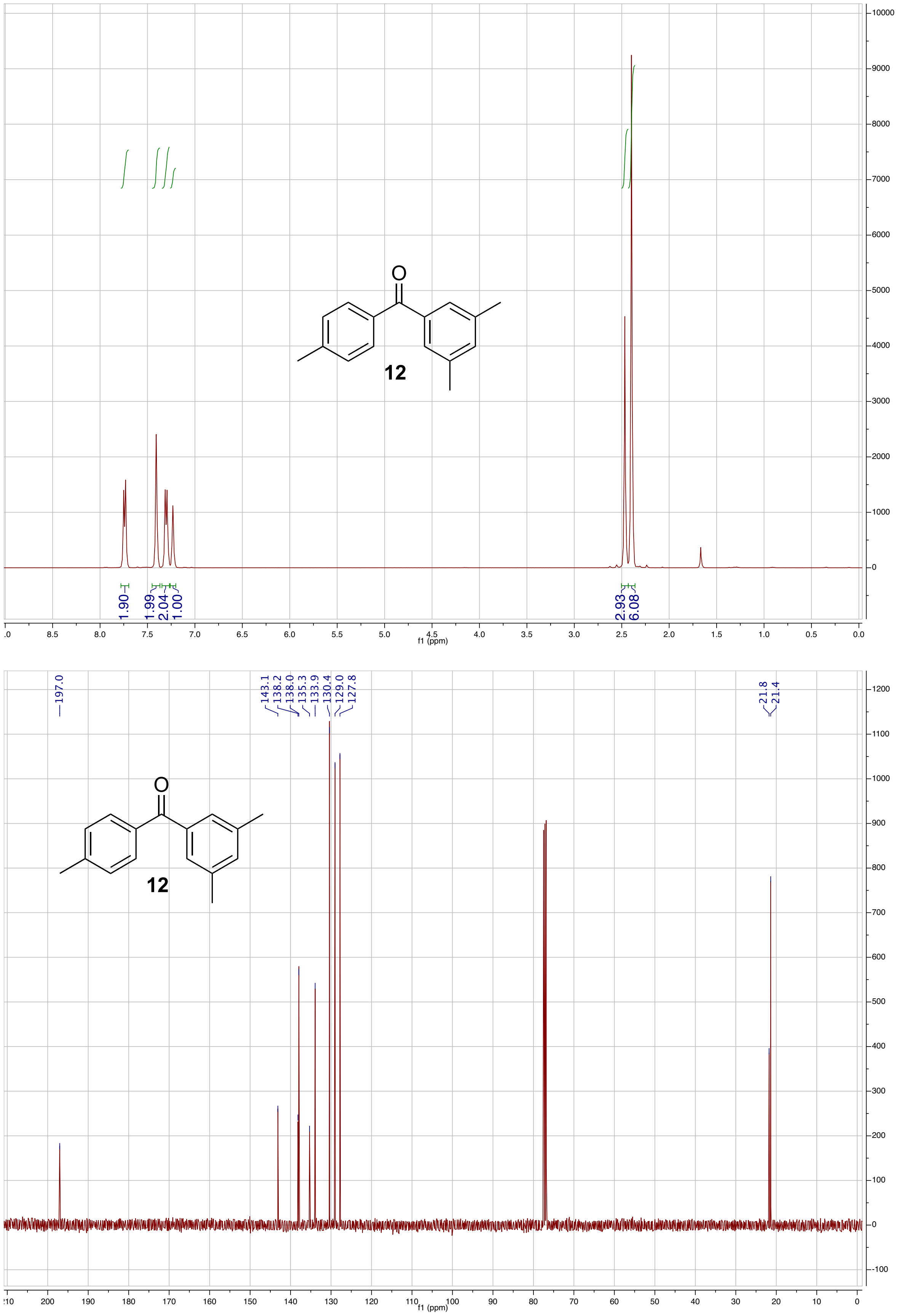

Supplementary Figure 10: ^{1}H and ^{13}C NMR of compound **12**.

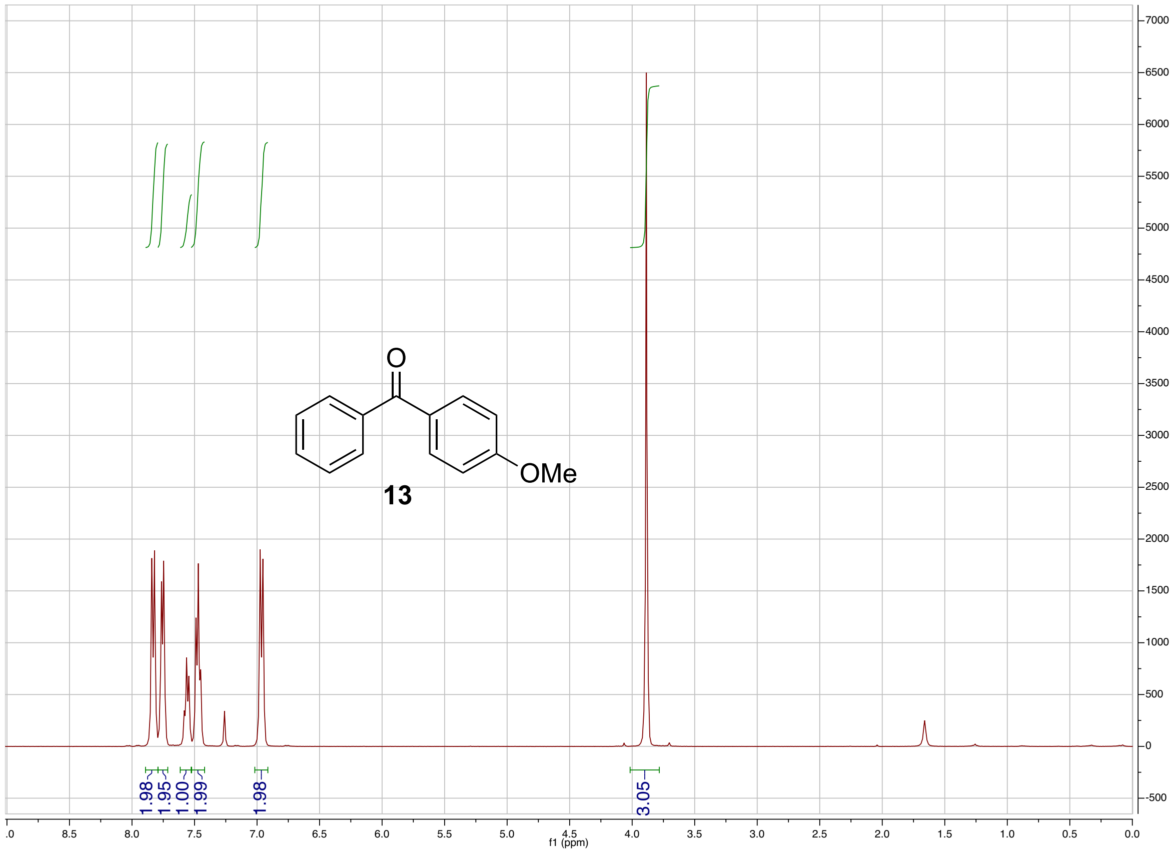

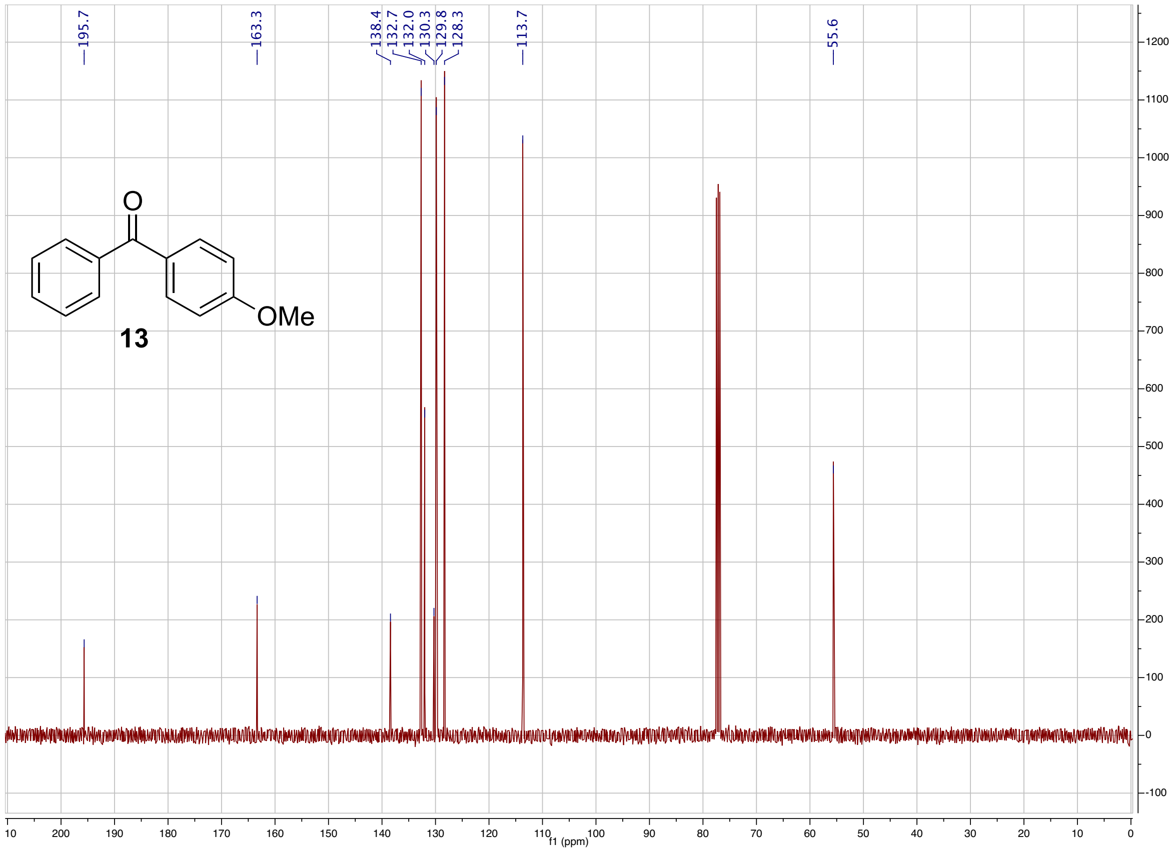

Supplementary Figure 11: ^{1}H and ^{13}C NMR of compound **13**.

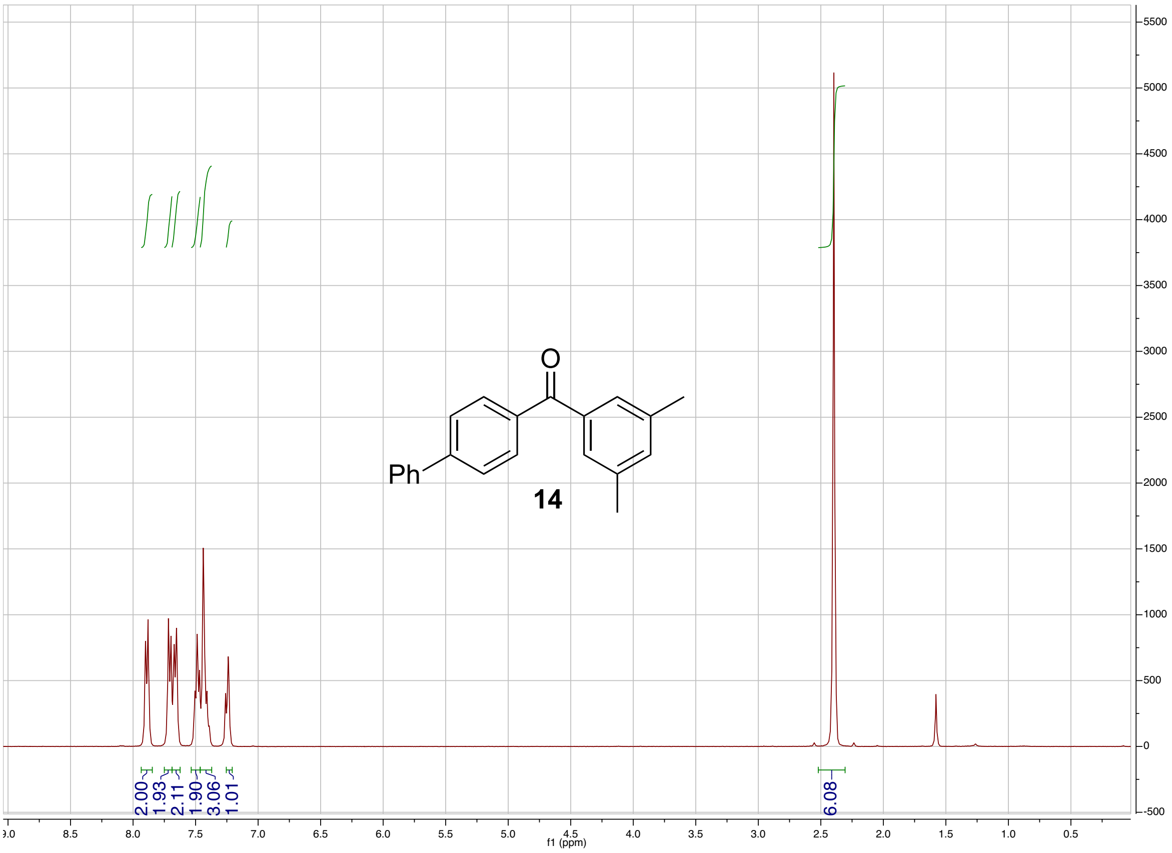

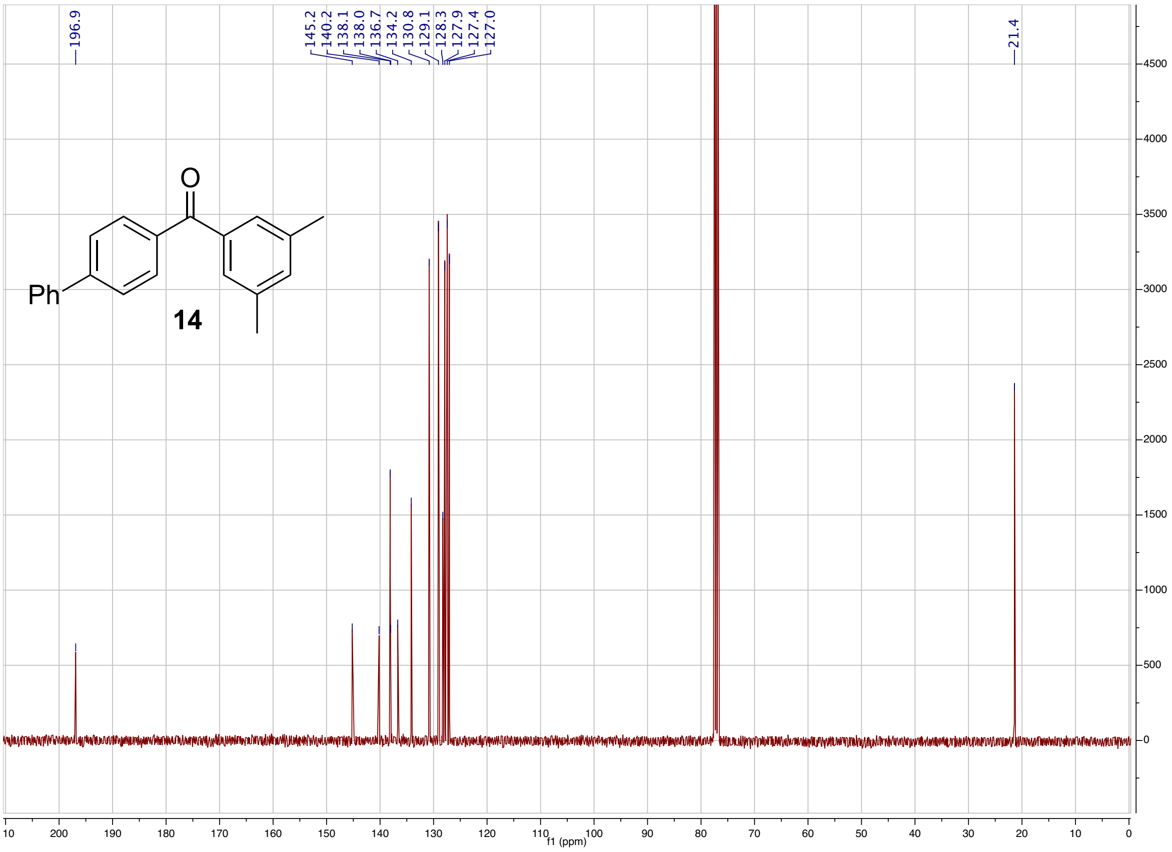

Supplementary Figure 12: ^{1}H and ^{13}C NMR of compound **14**.

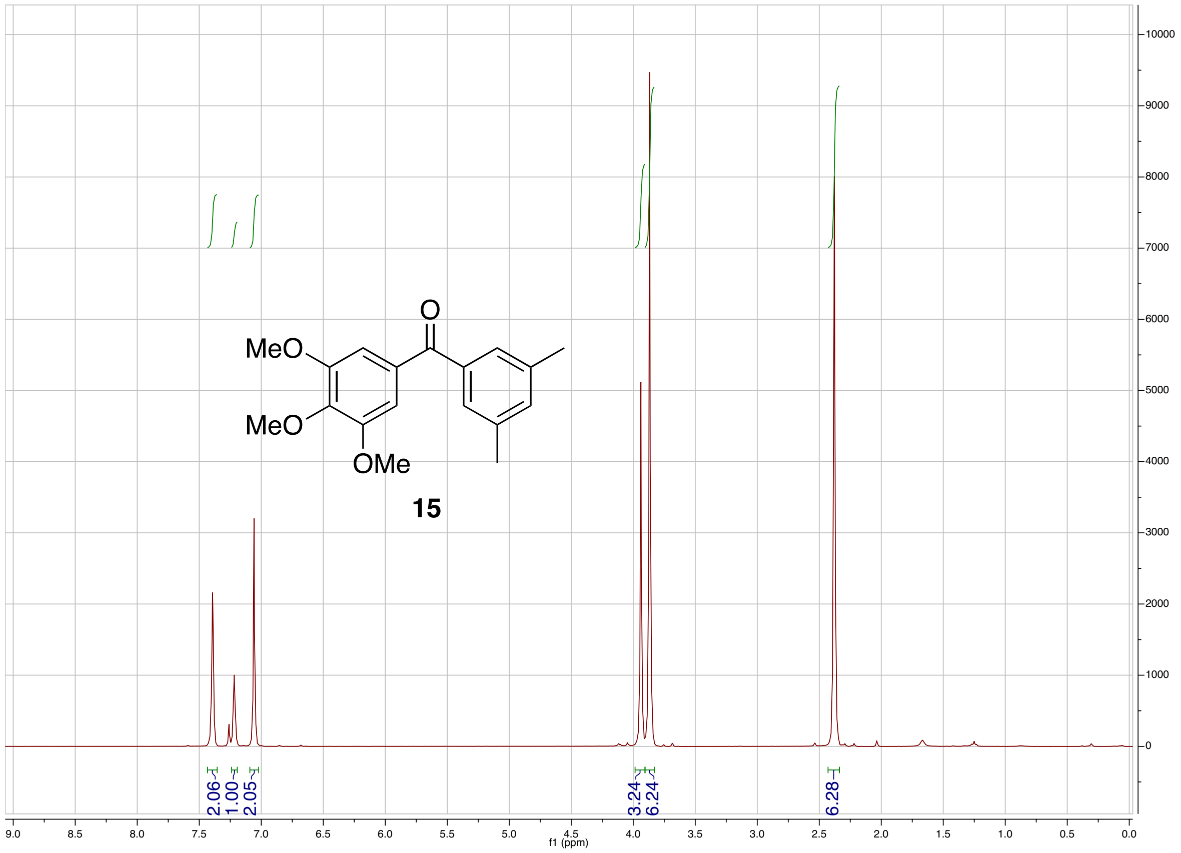

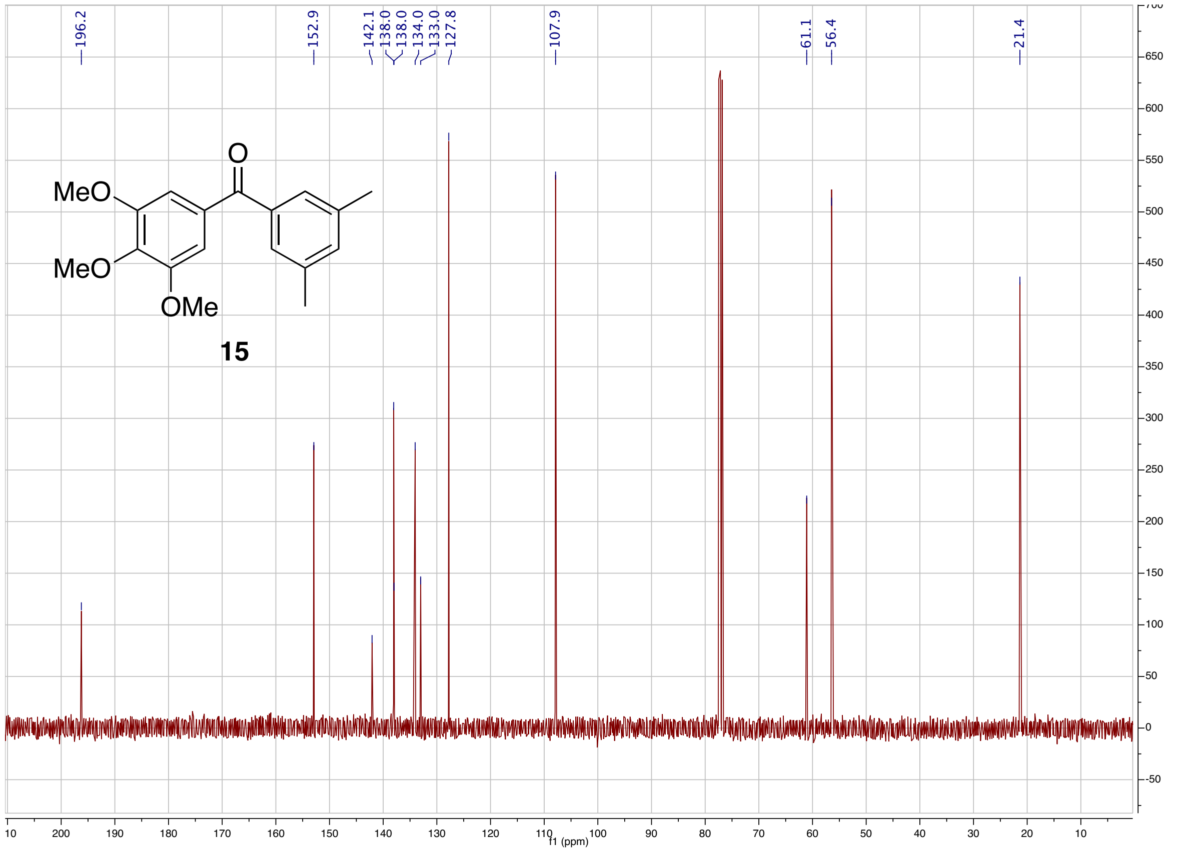

Supplementary Figure 13: ^{1}H and ^{13}C NMR of compound **15**.

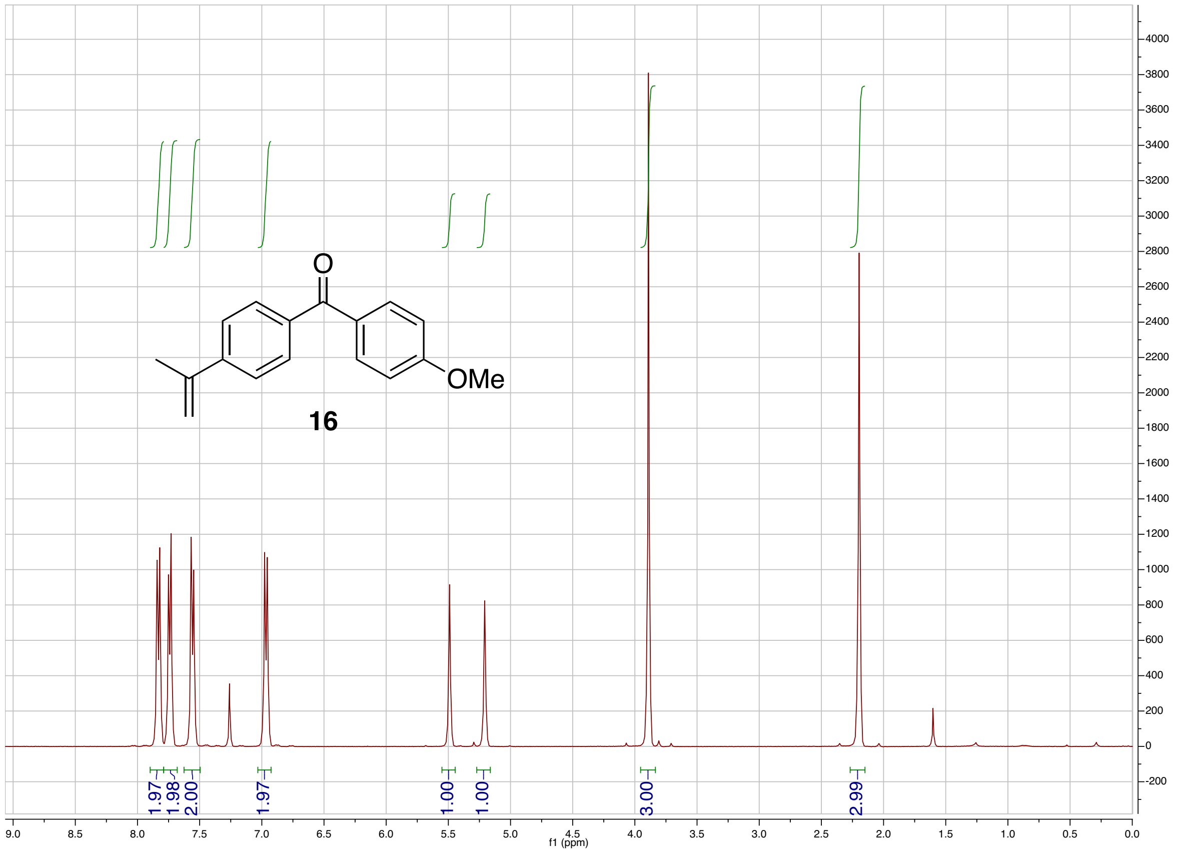

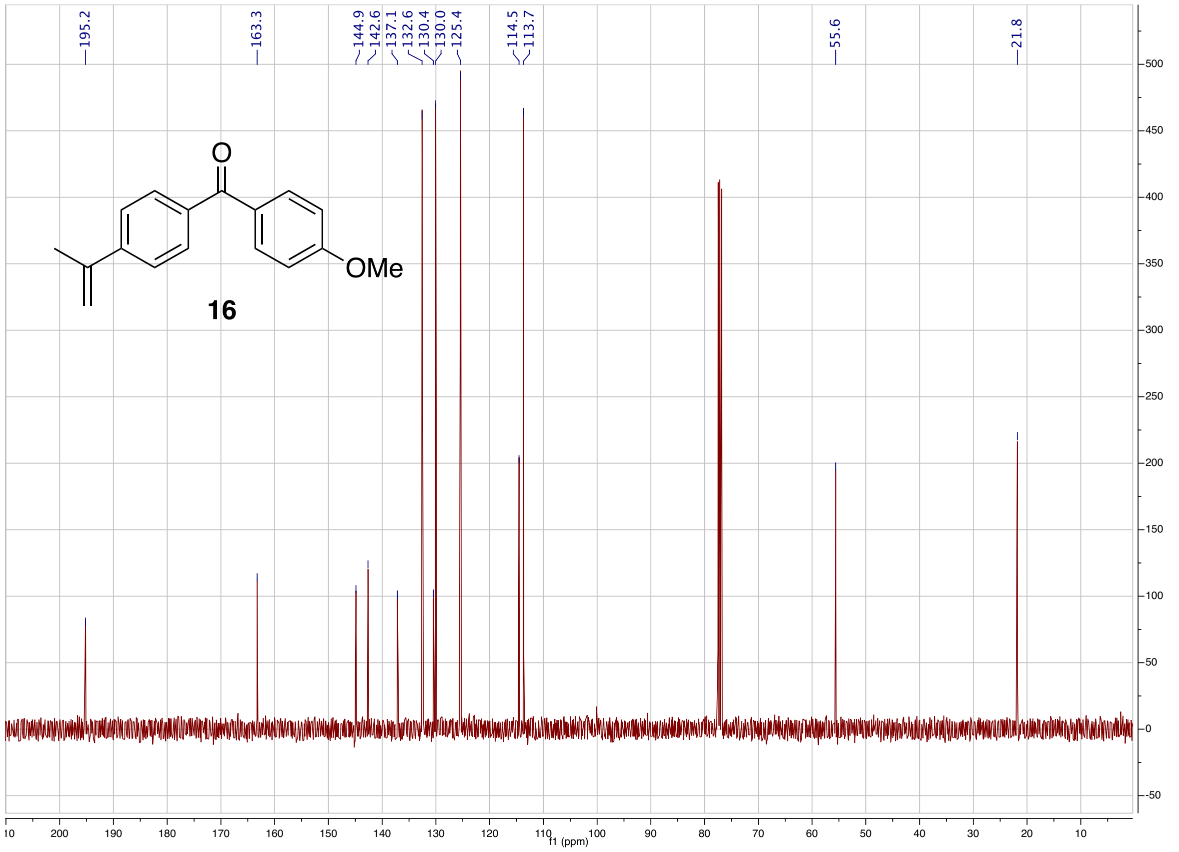

Supplementary Figure 14: ^{1}H and ^{13}C NMR of compound **16**.

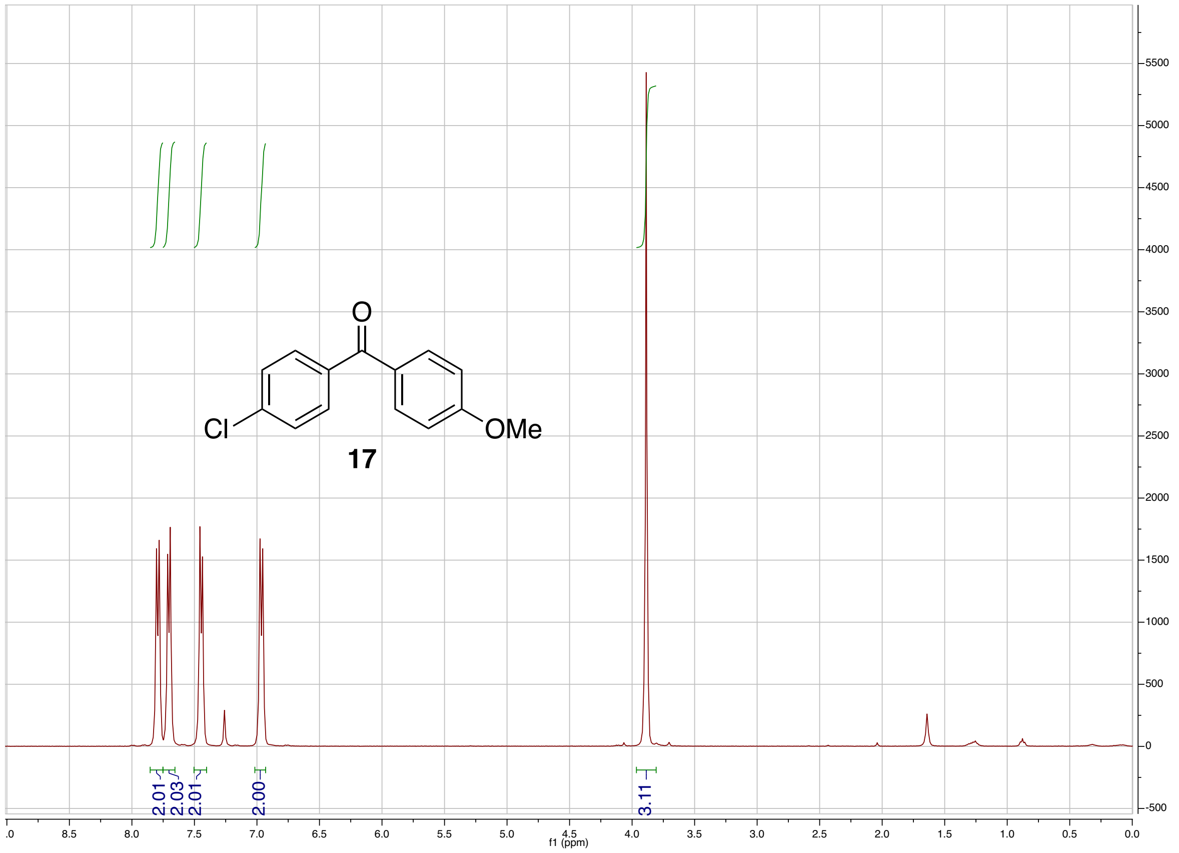

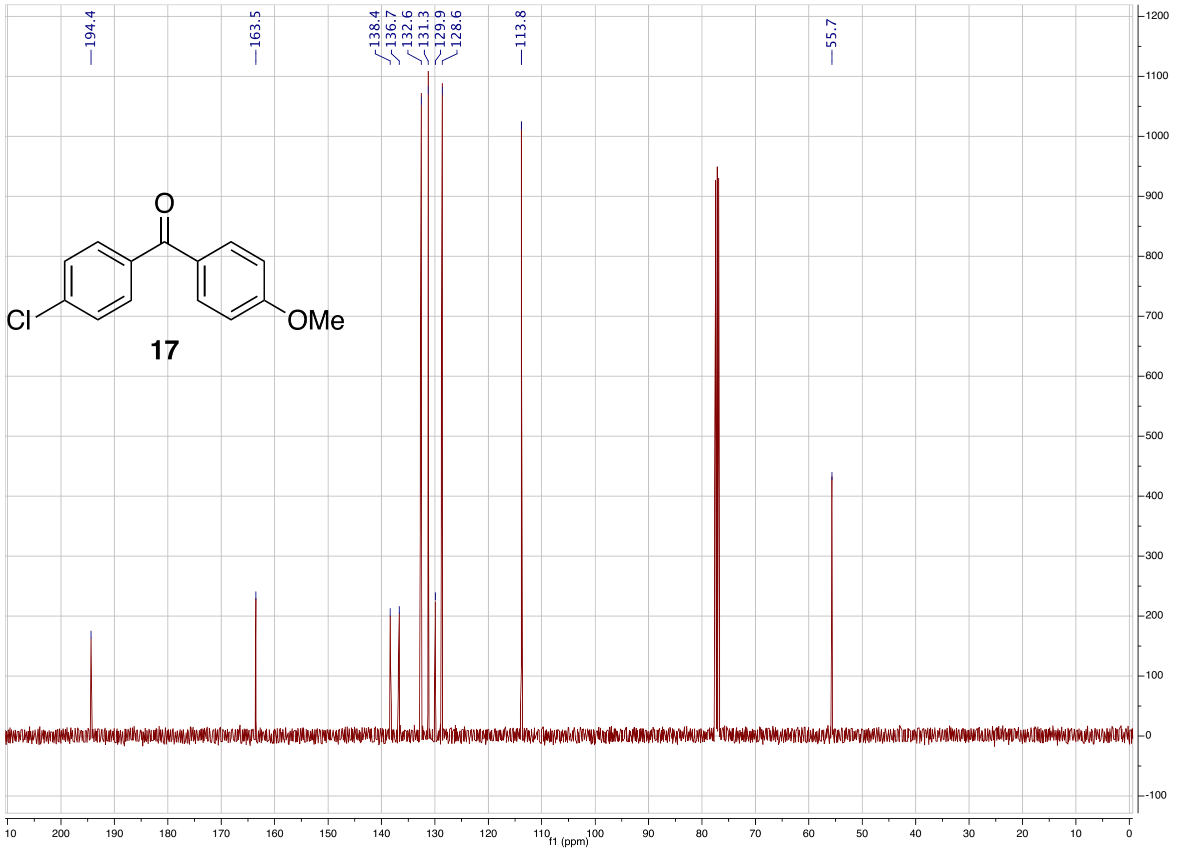

Supplementary Figure 15: ^{1}H and ^{13}C NMR of compound **17**.

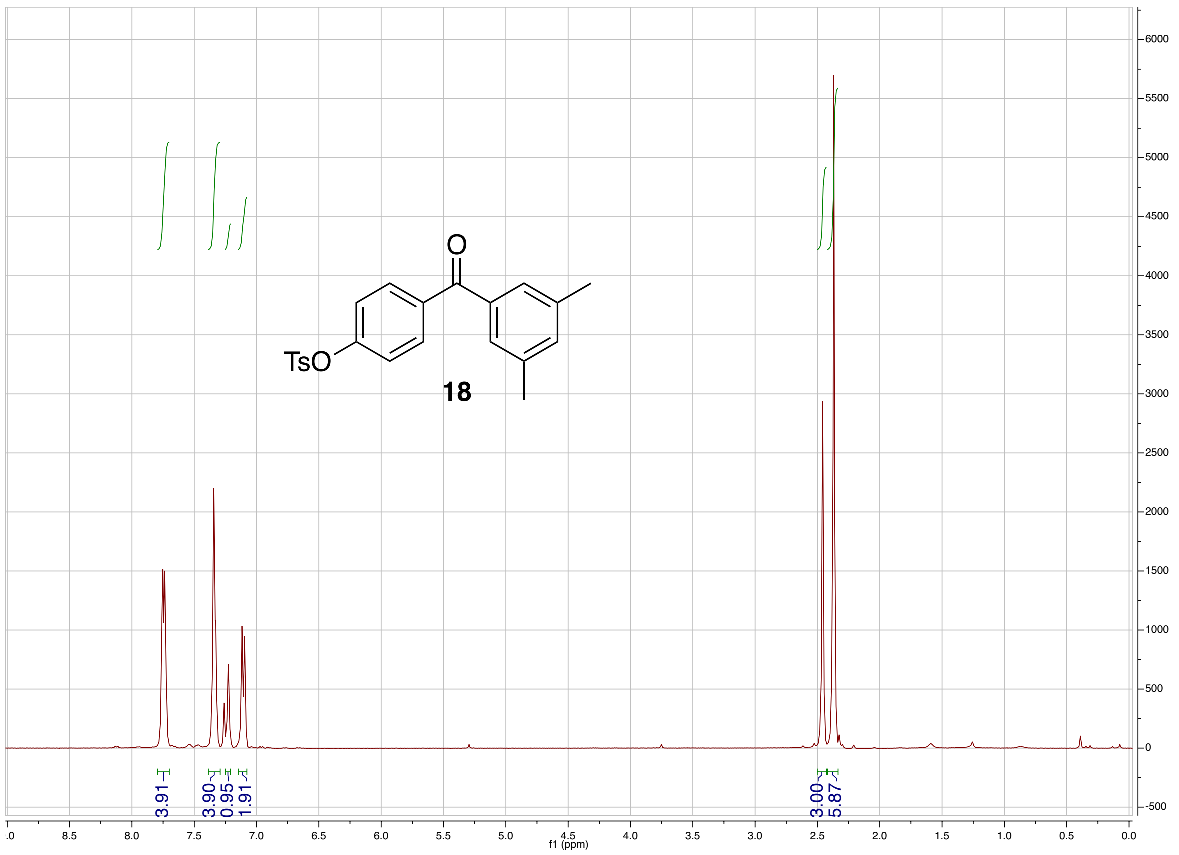

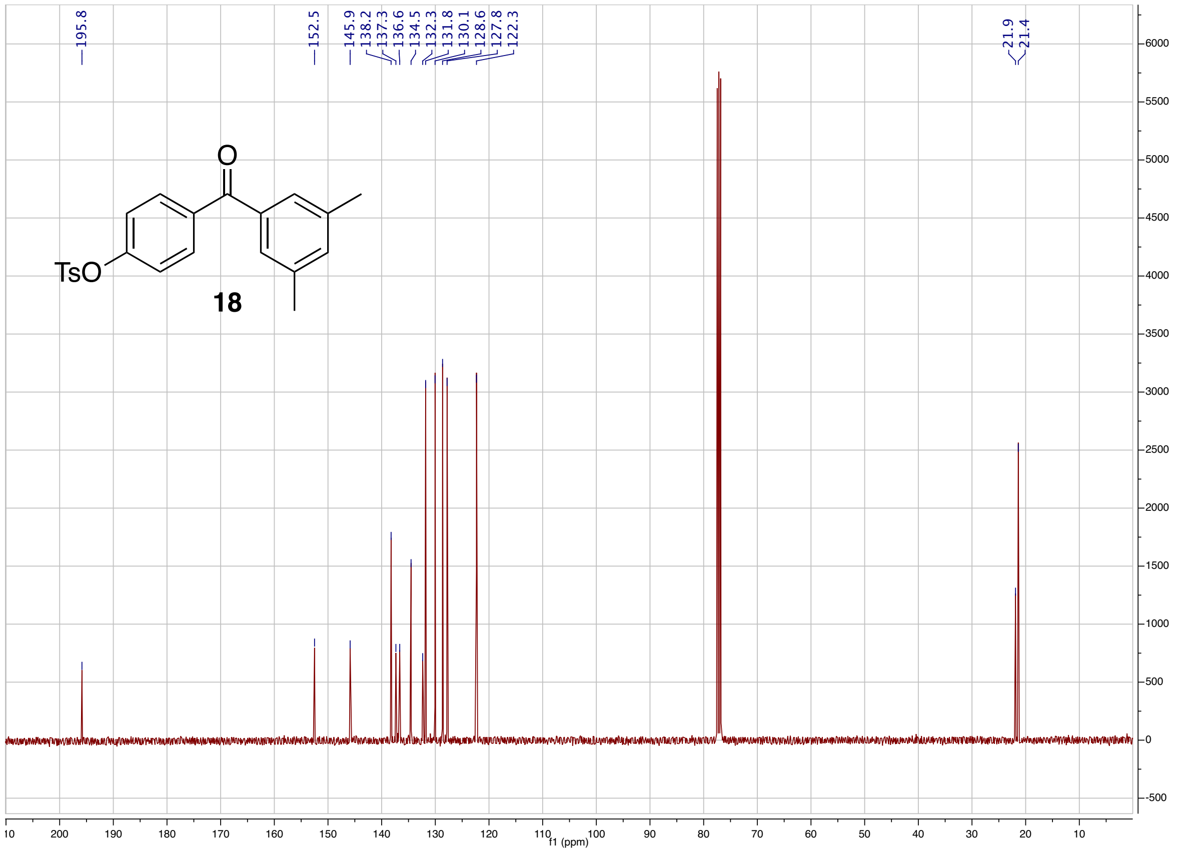

Supplementary Figure 16: ^{1}H and ^{13}C NMR of compound **18**.

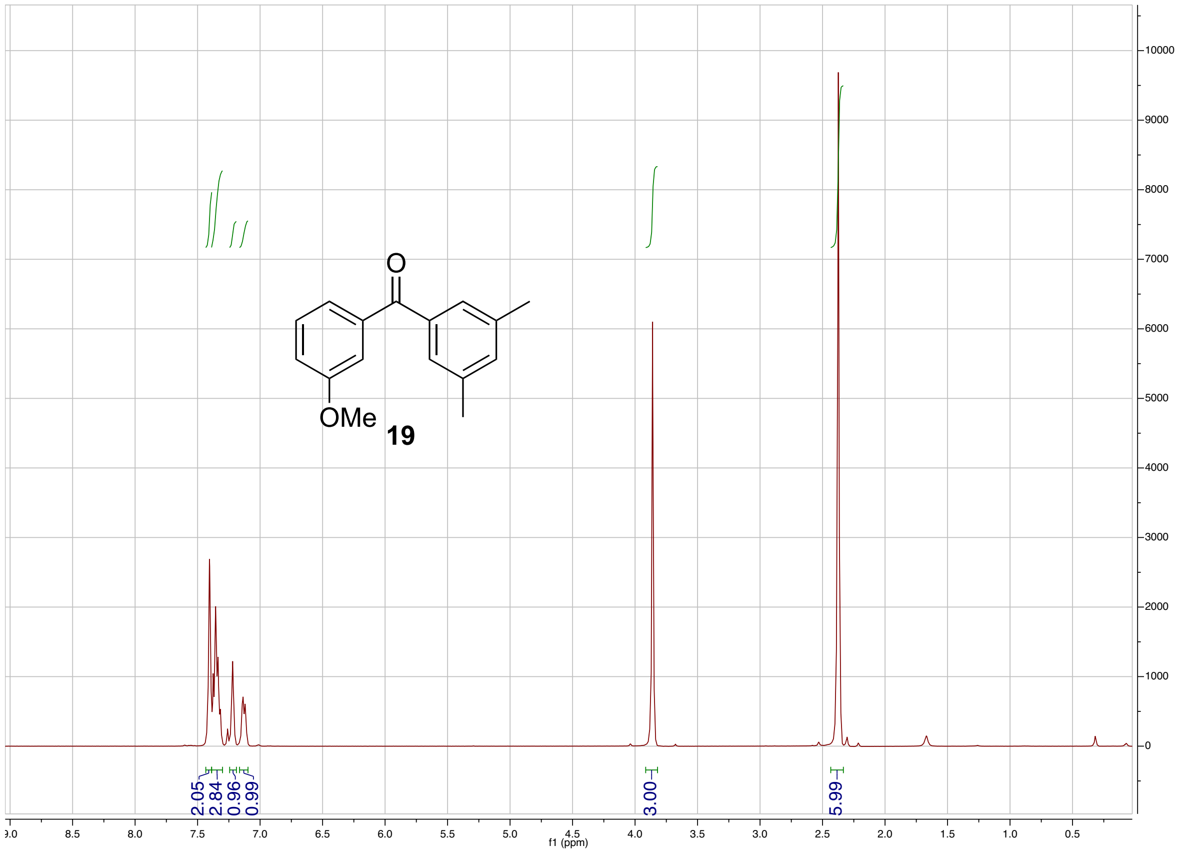

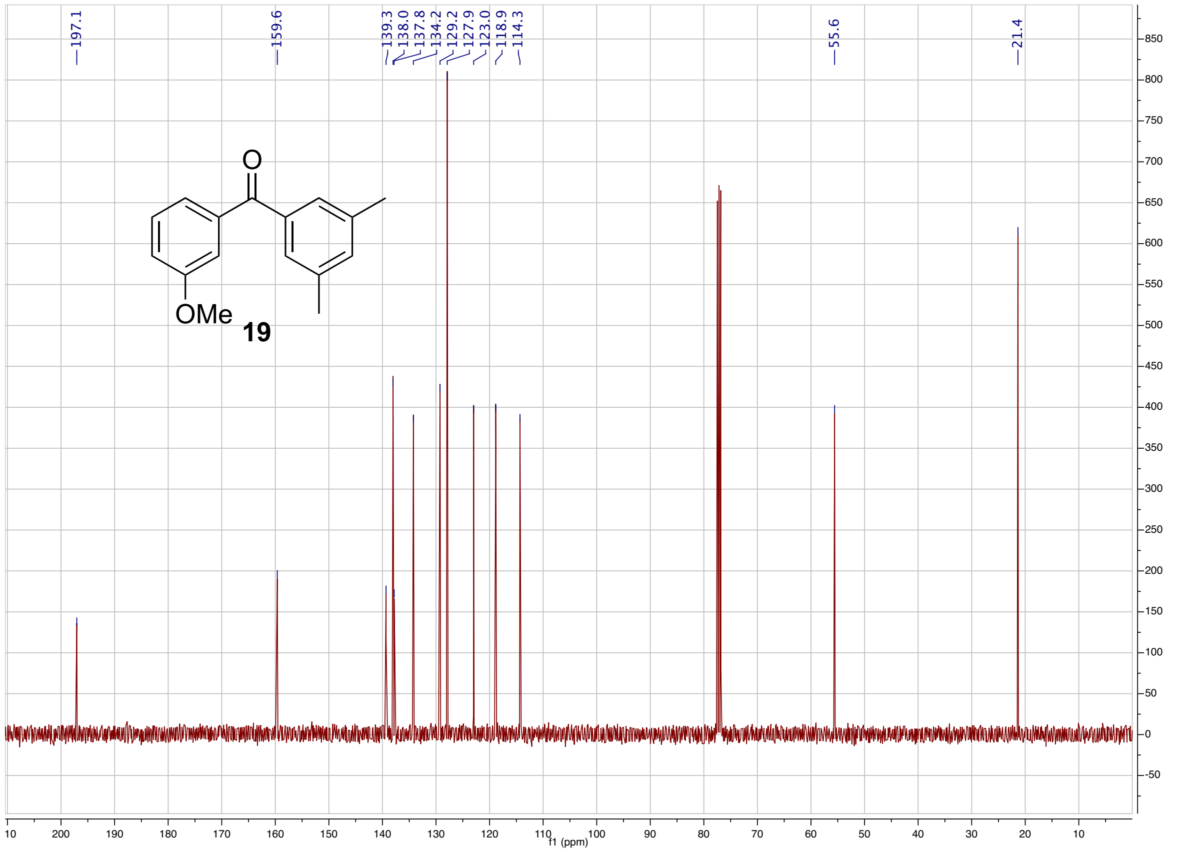

Supplementary Figure 17: ^{1}H and ^{13}C NMR of compound **19**.

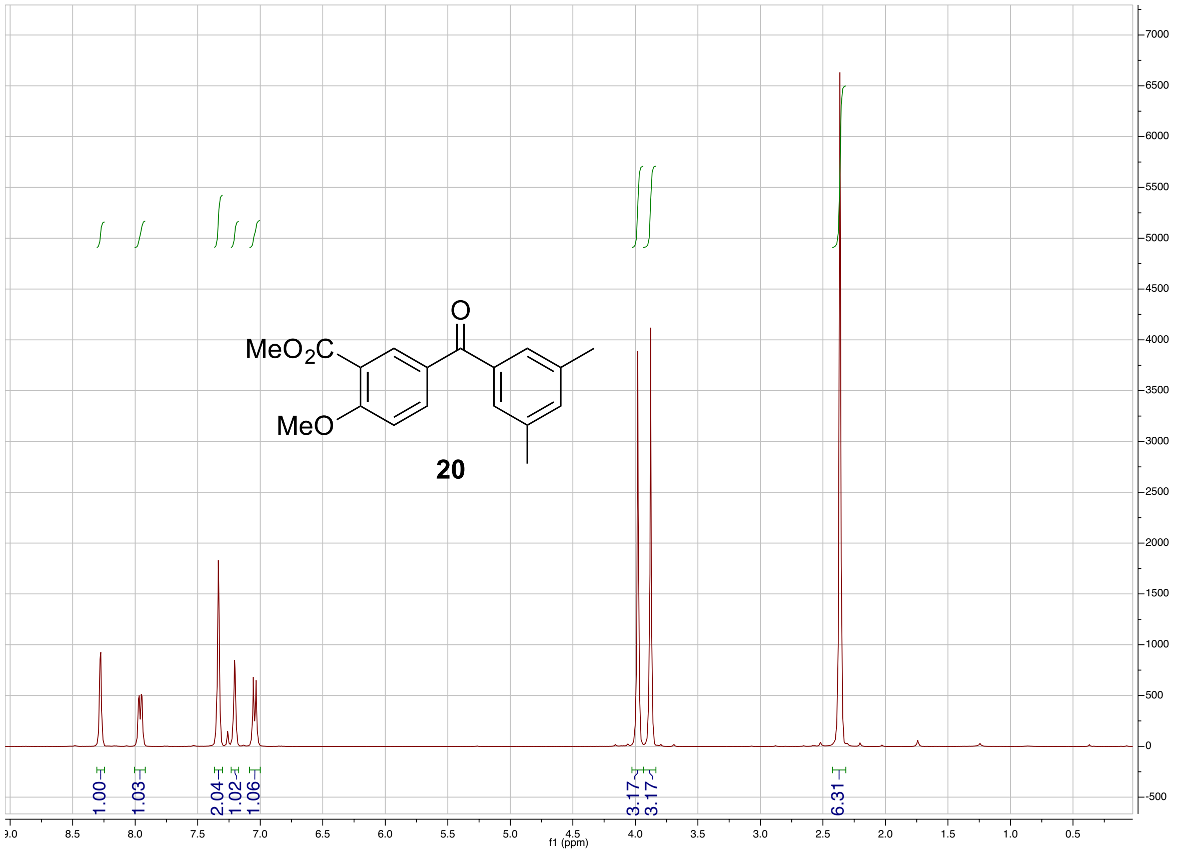

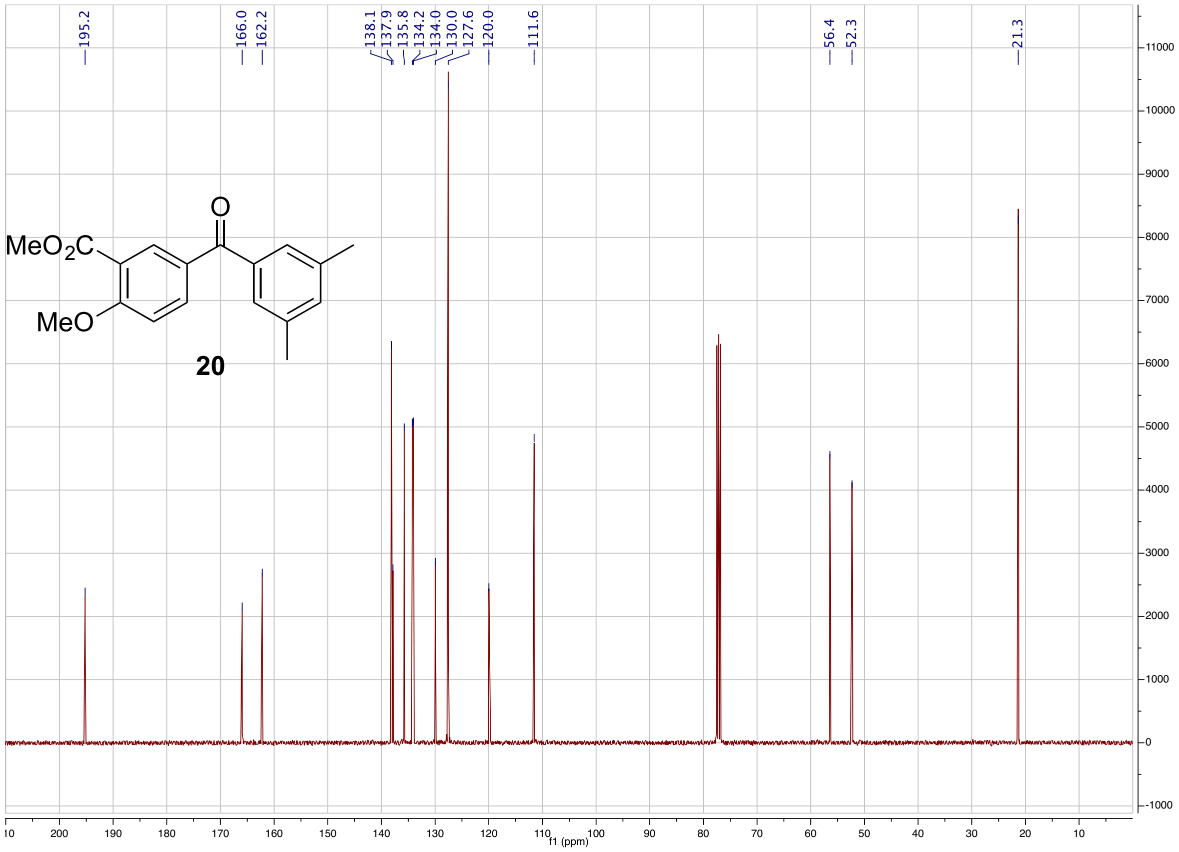

Supplementary Figure 18: ^{1}H and ^{13}C NMR of compound **20**.

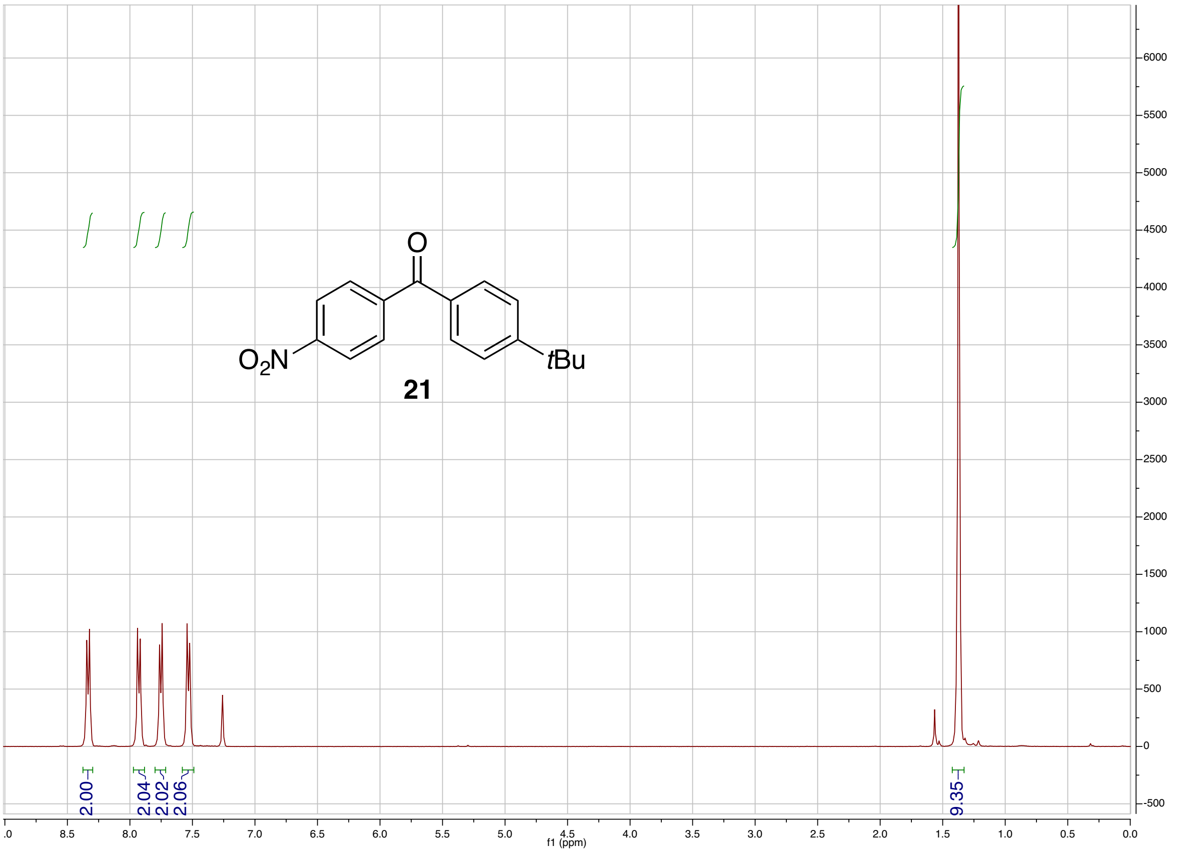

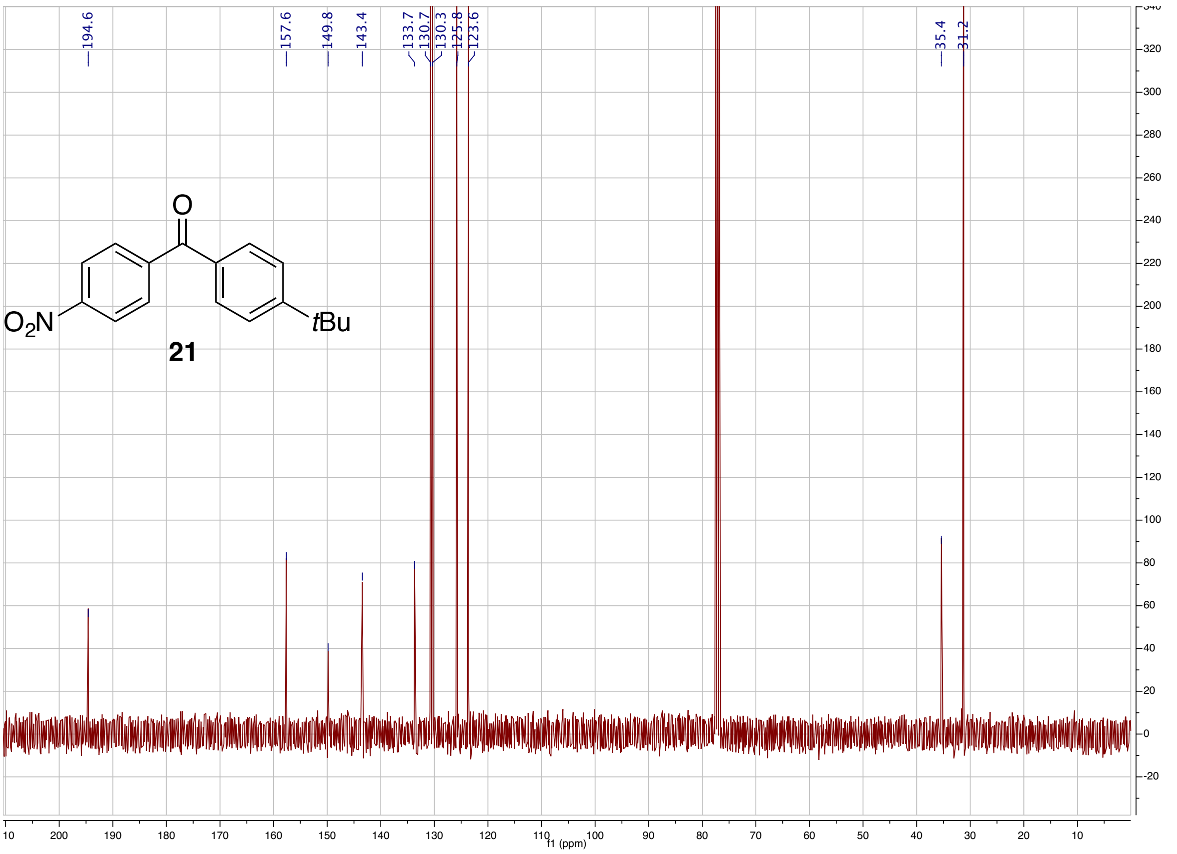

Supplementary Figure 19: ^{1}H and ^{13}C NMR of compound **21**.

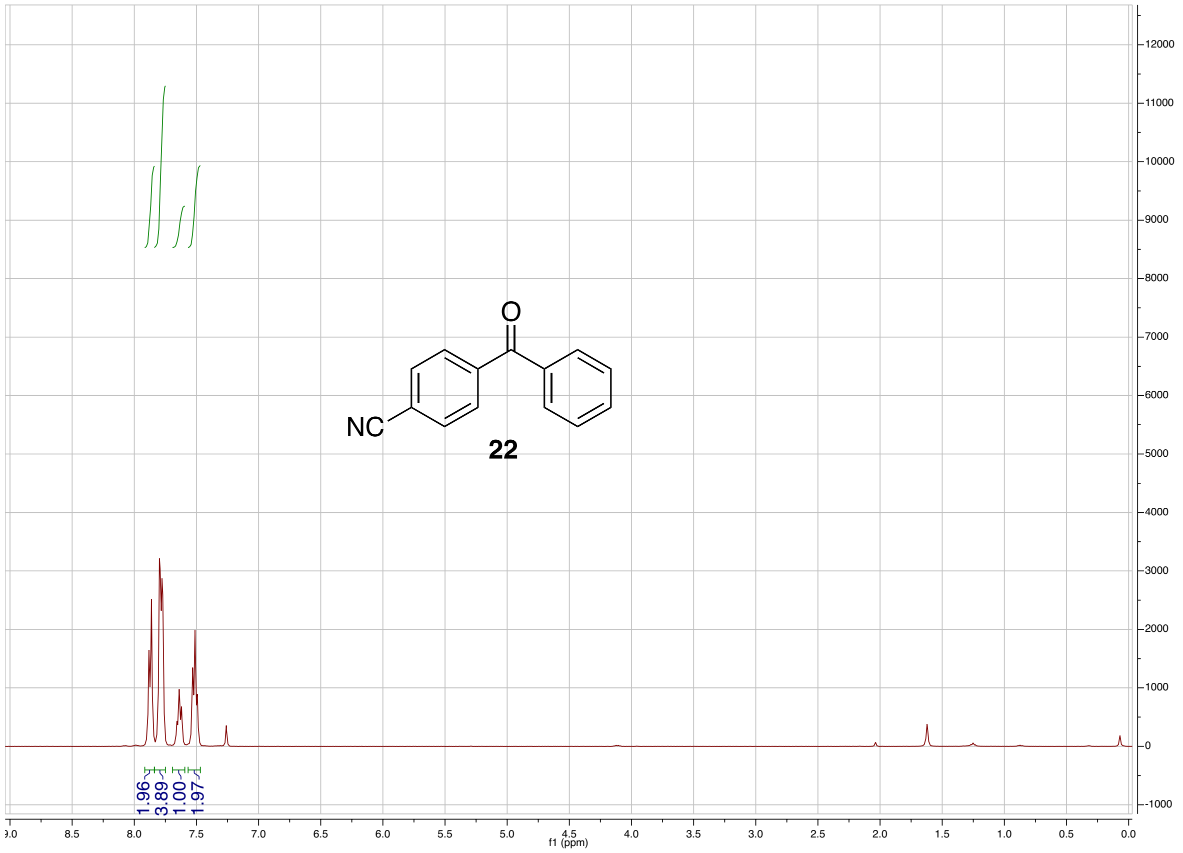

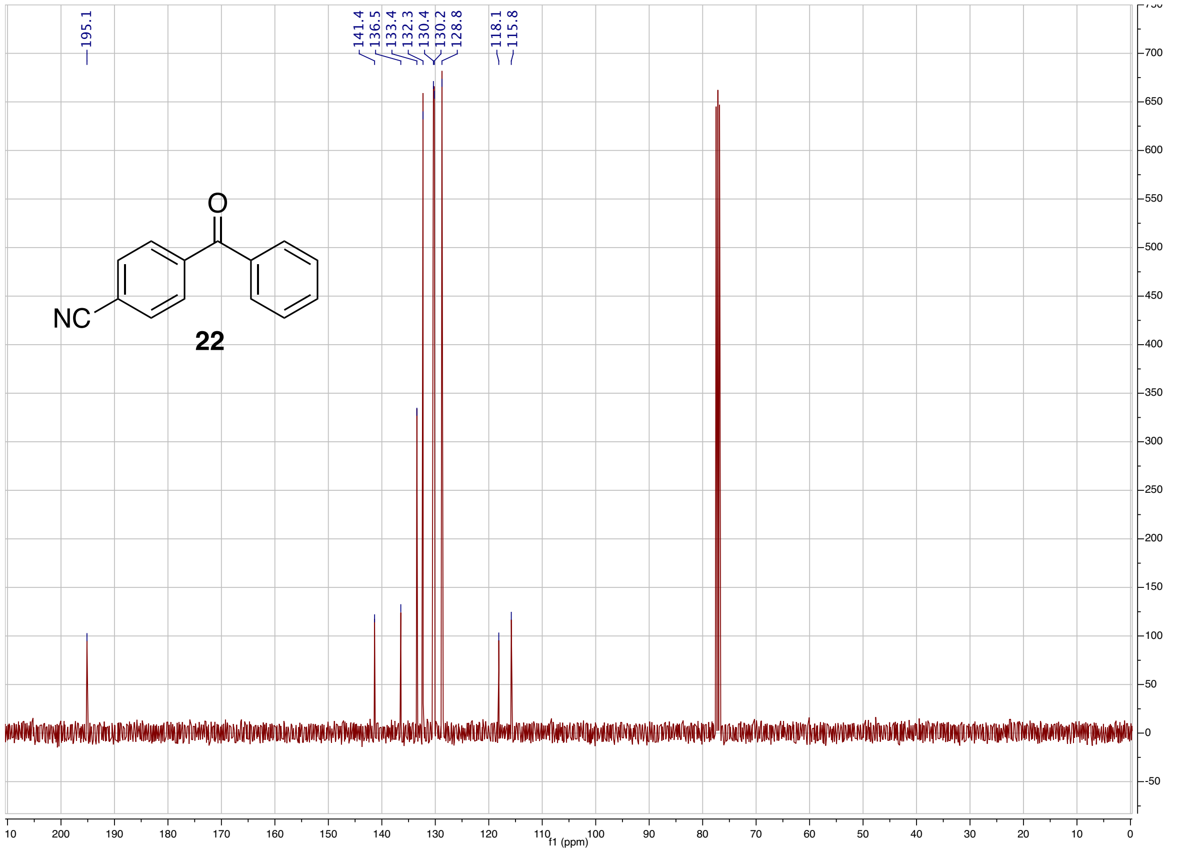

Supplementary Figure 20: ^{1}H and ^{13}C NMR of compound **22**.

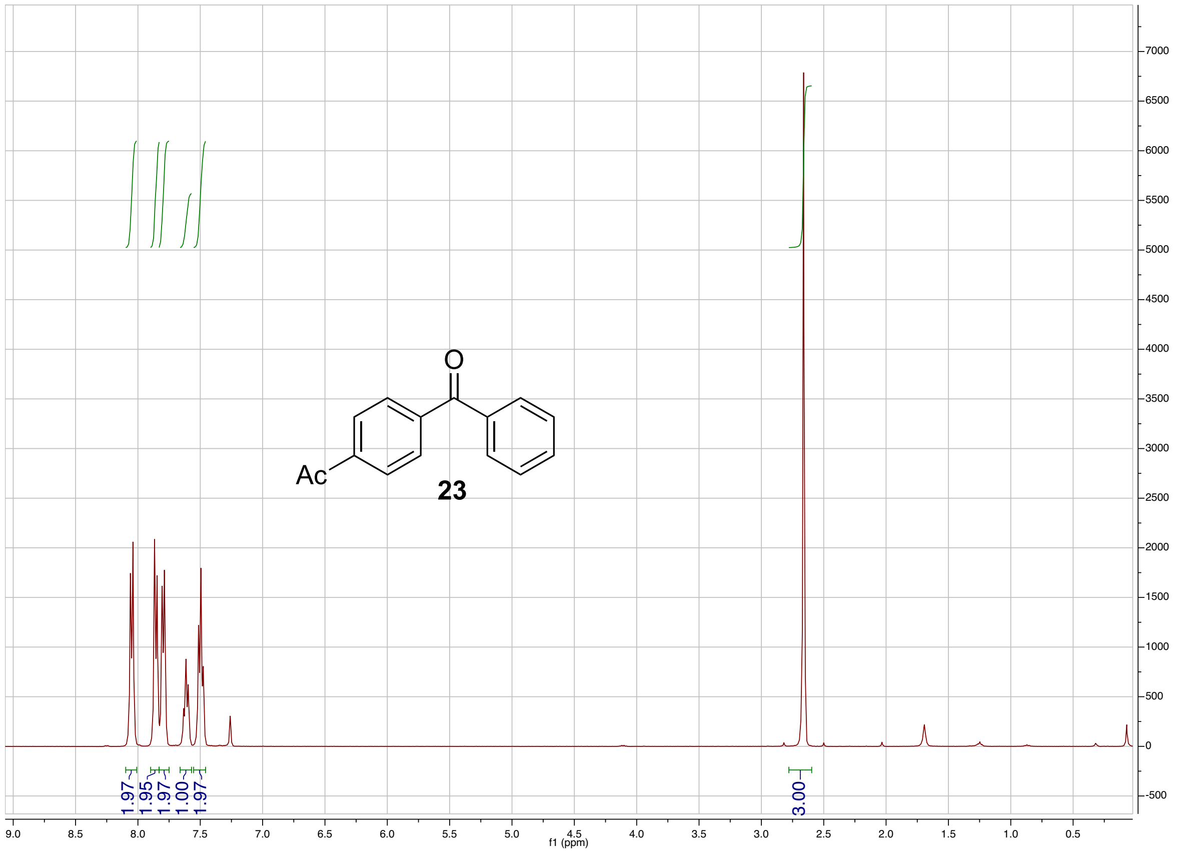

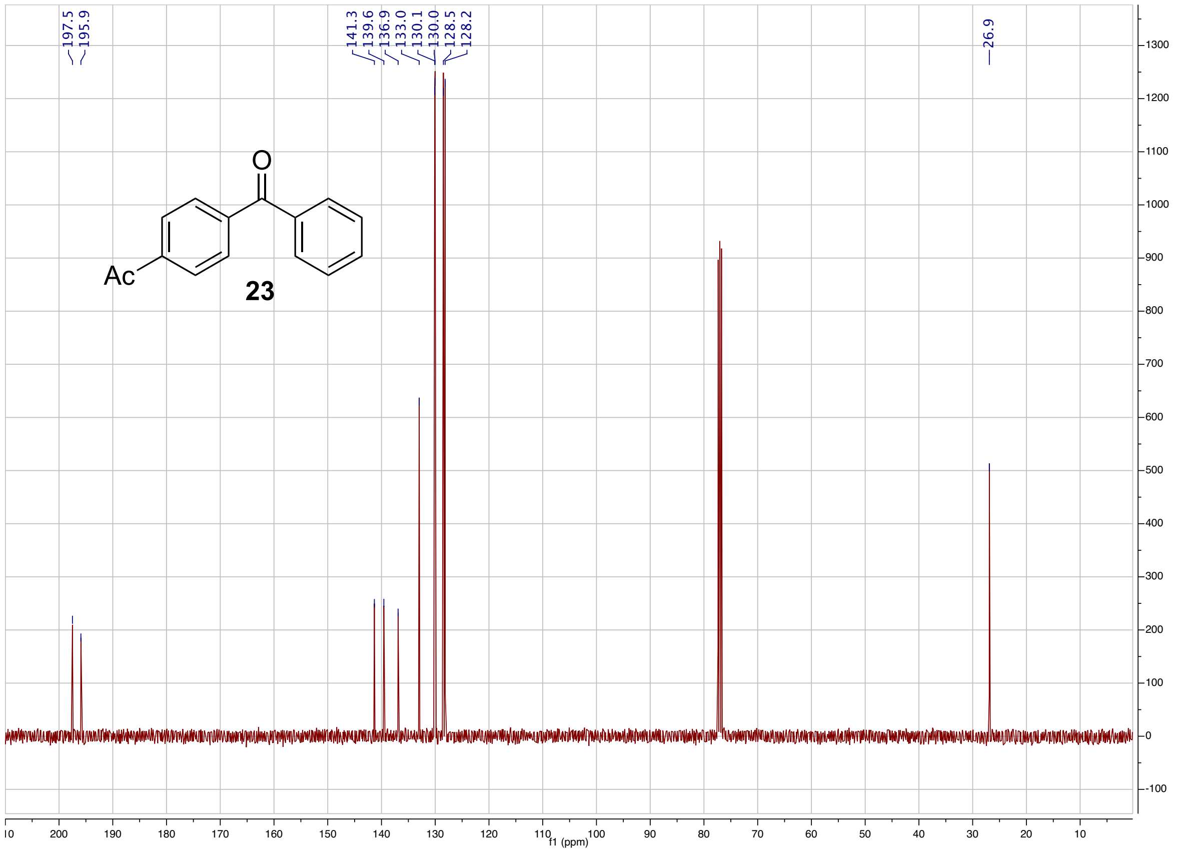

Supplementary Figure 21: ^{1}H and ^{13}C NMR of compound **23**.

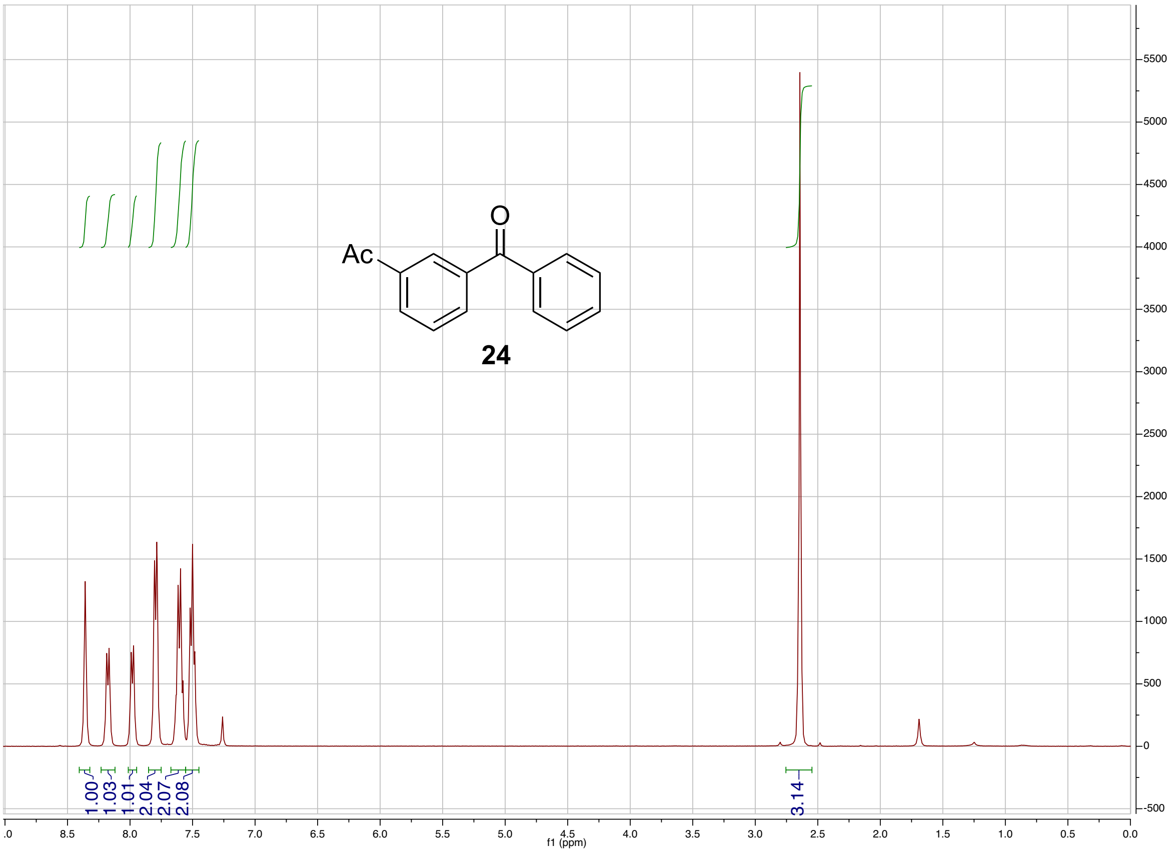

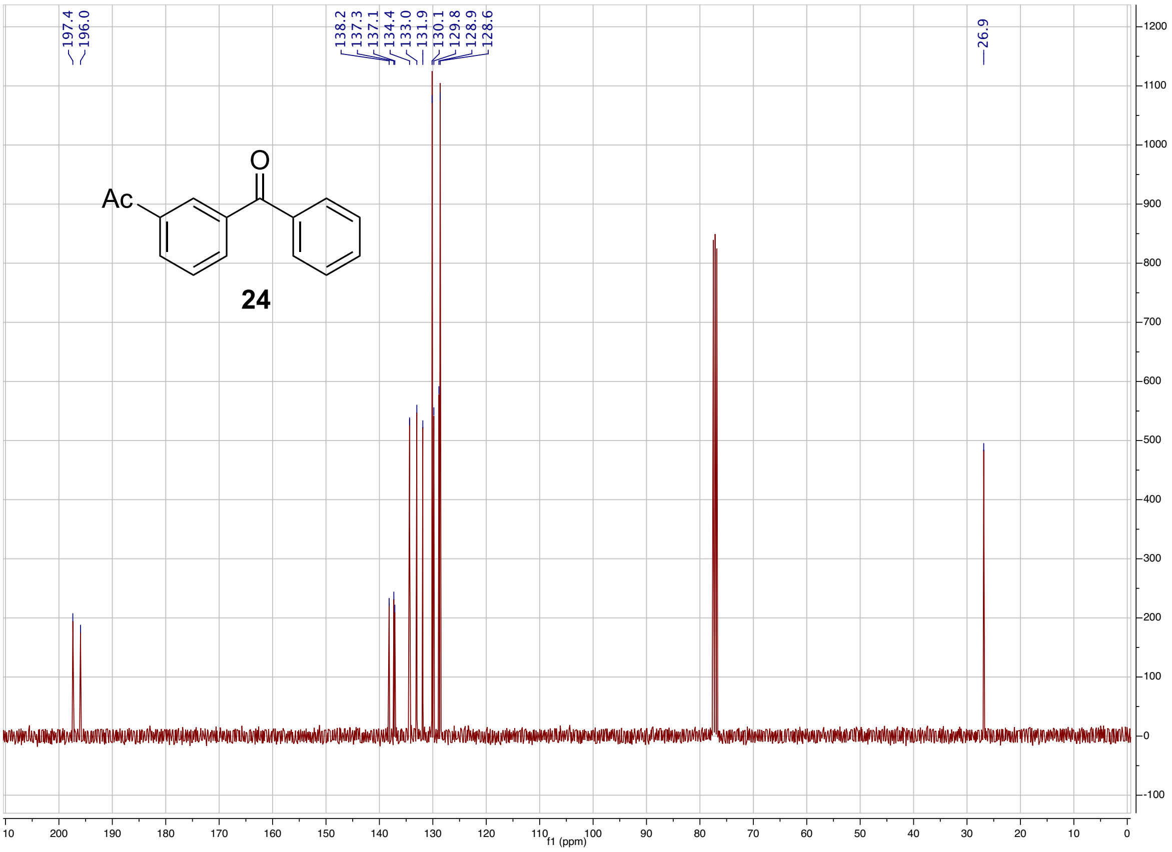

Supplementary Figure 22: ^{1}H and ^{13}C NMR of compound **24**.

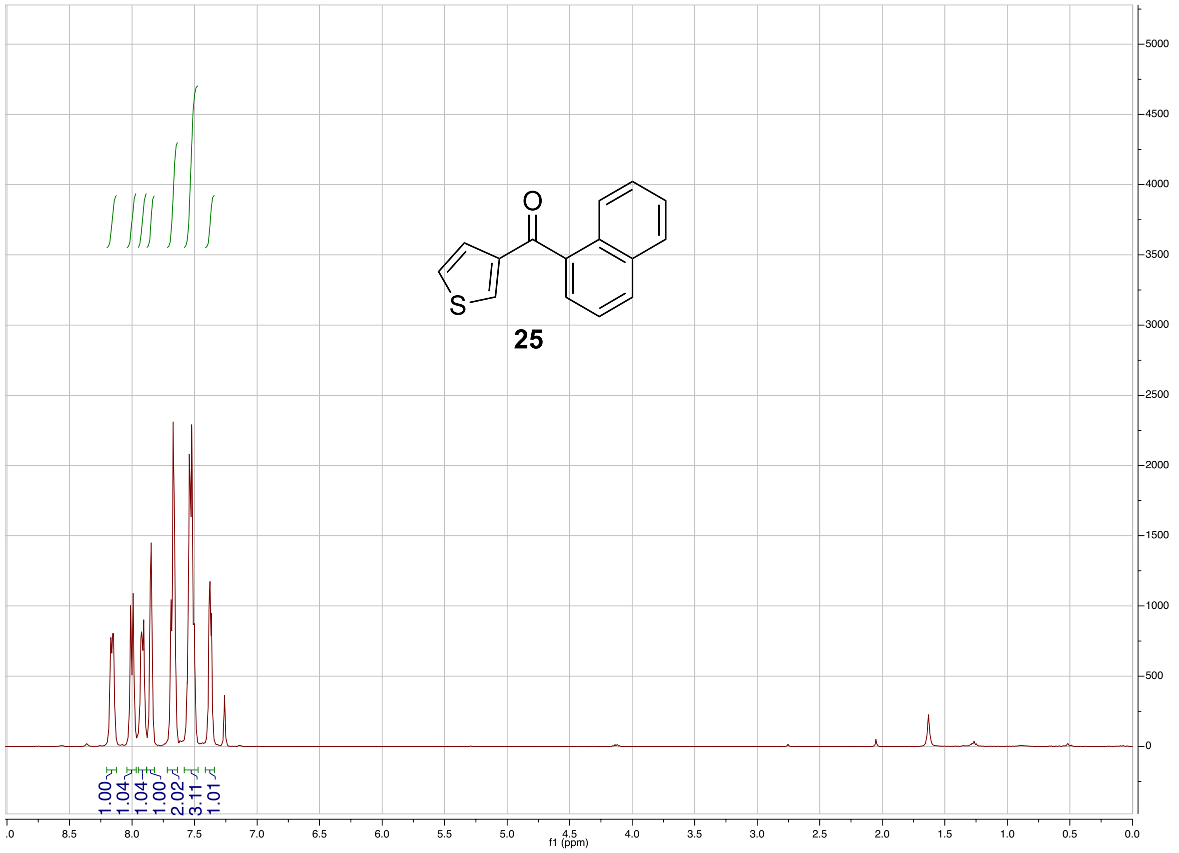

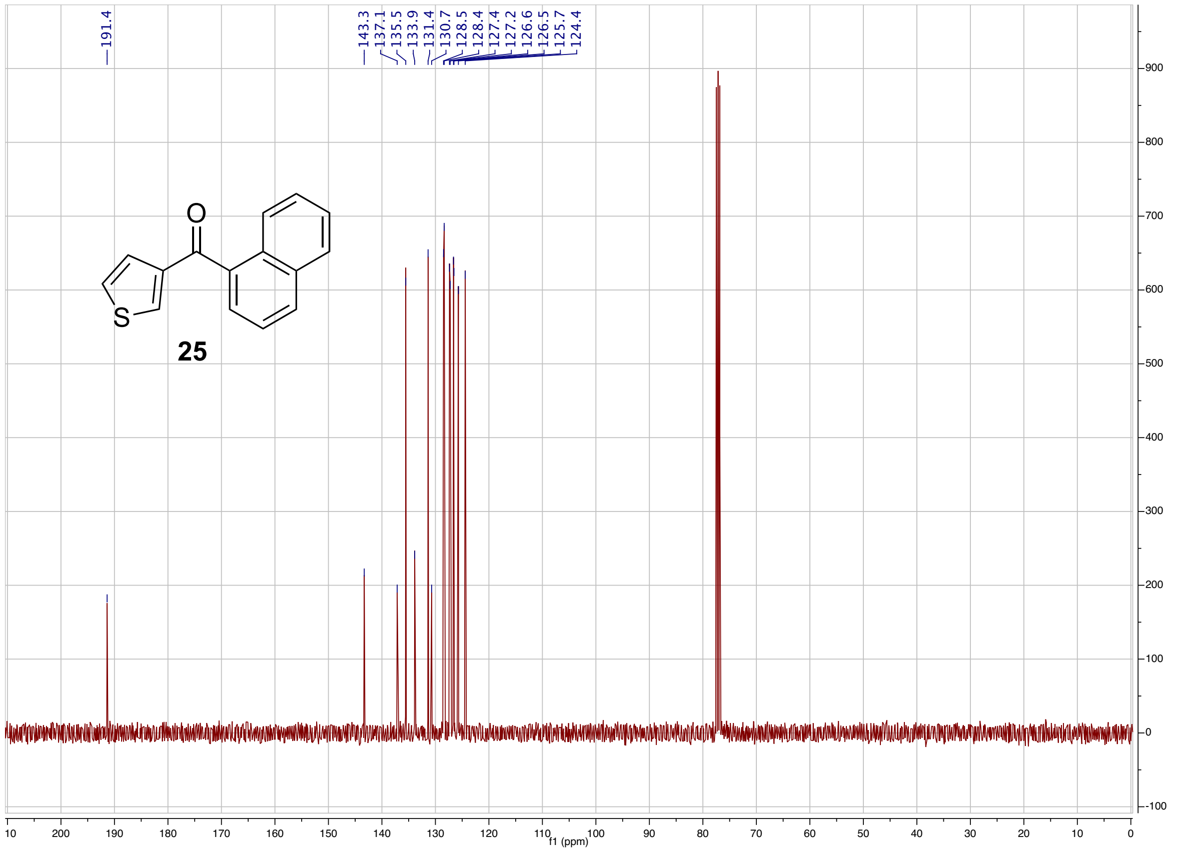

Supplementary Figure 23: ^{1}H and ^{13}C NMR of compound **25**.

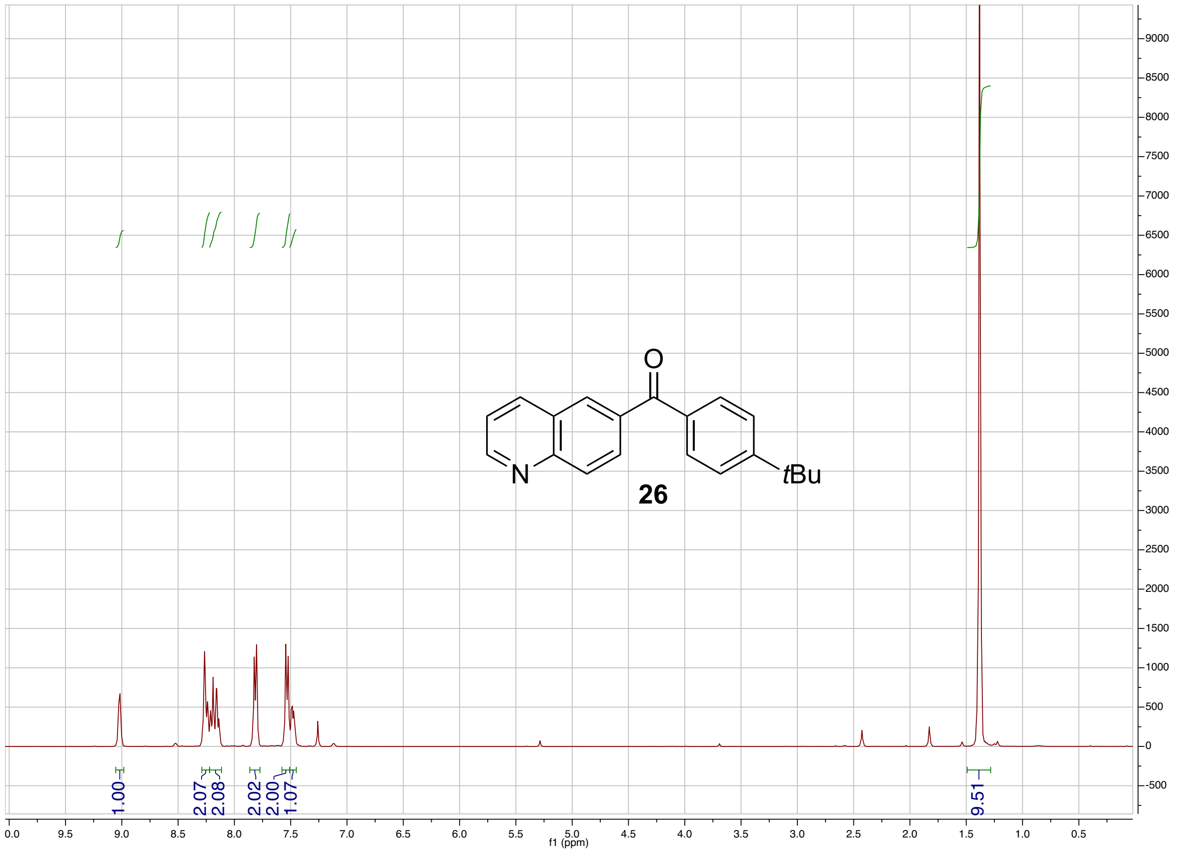

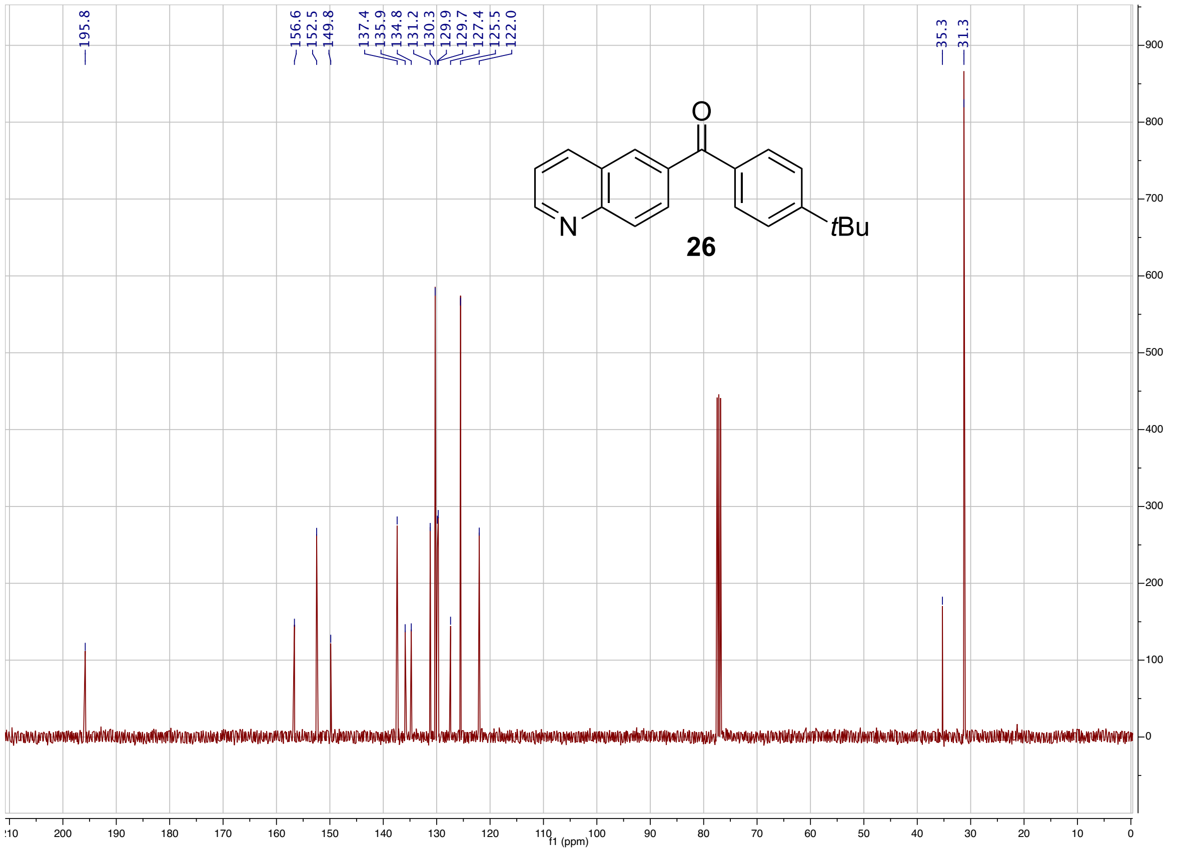

Supplementary Figure 24: ^{1}H and ^{13}C NMR of compound **26**.

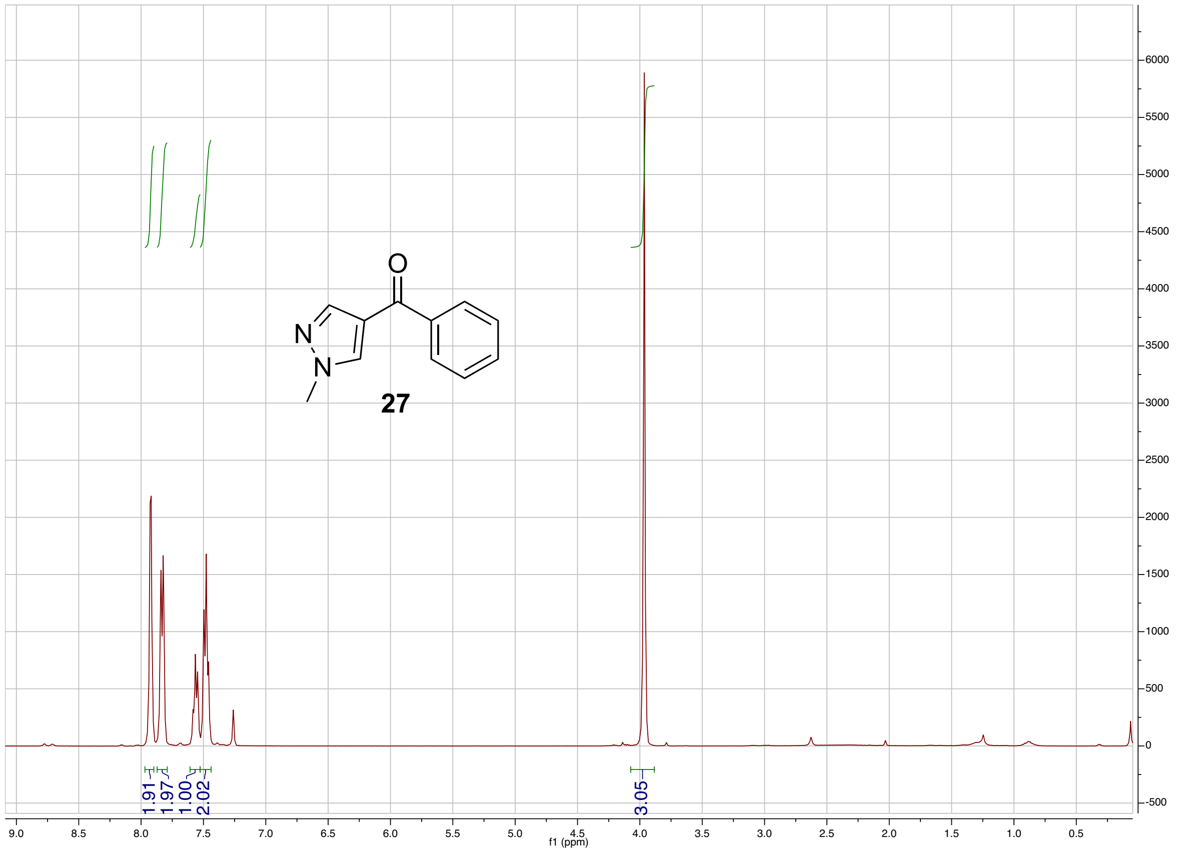

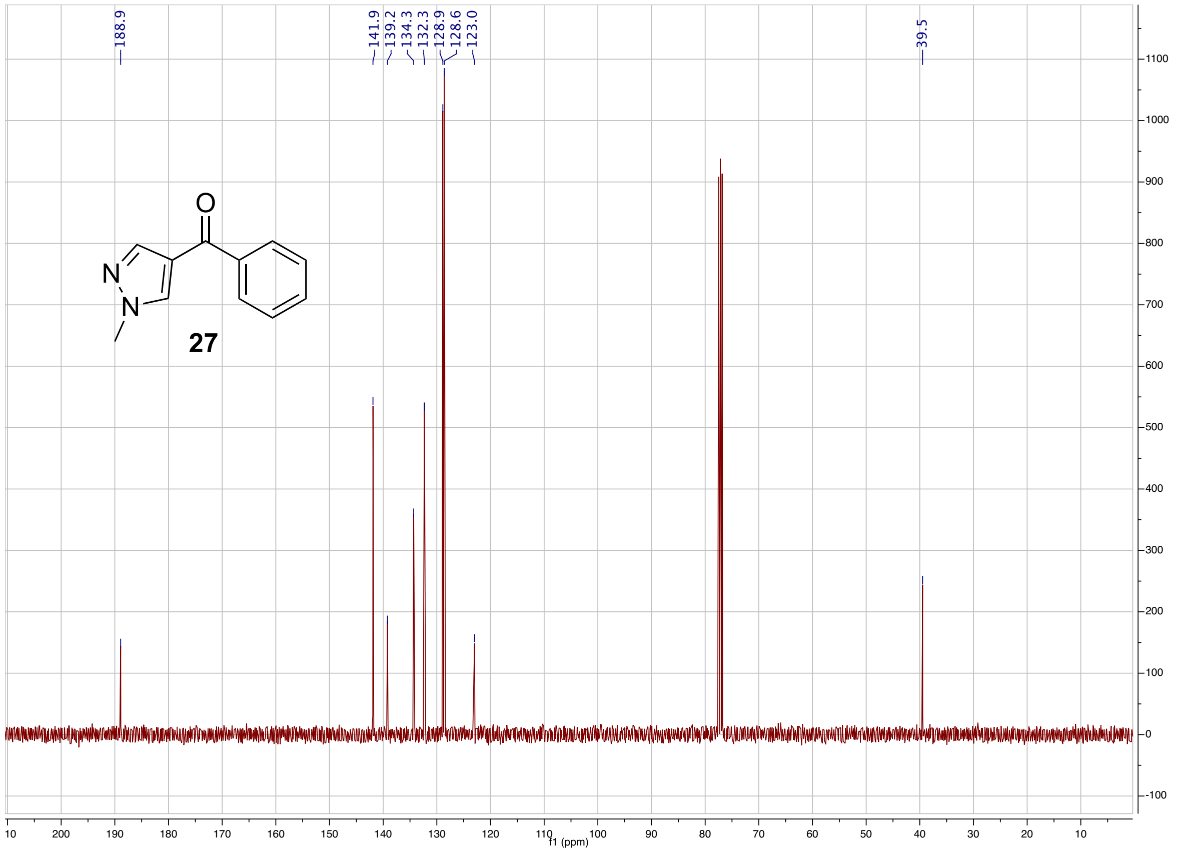

Supplementary Figure 25: ^{1}H and ^{13}C NMR of compound **27**.

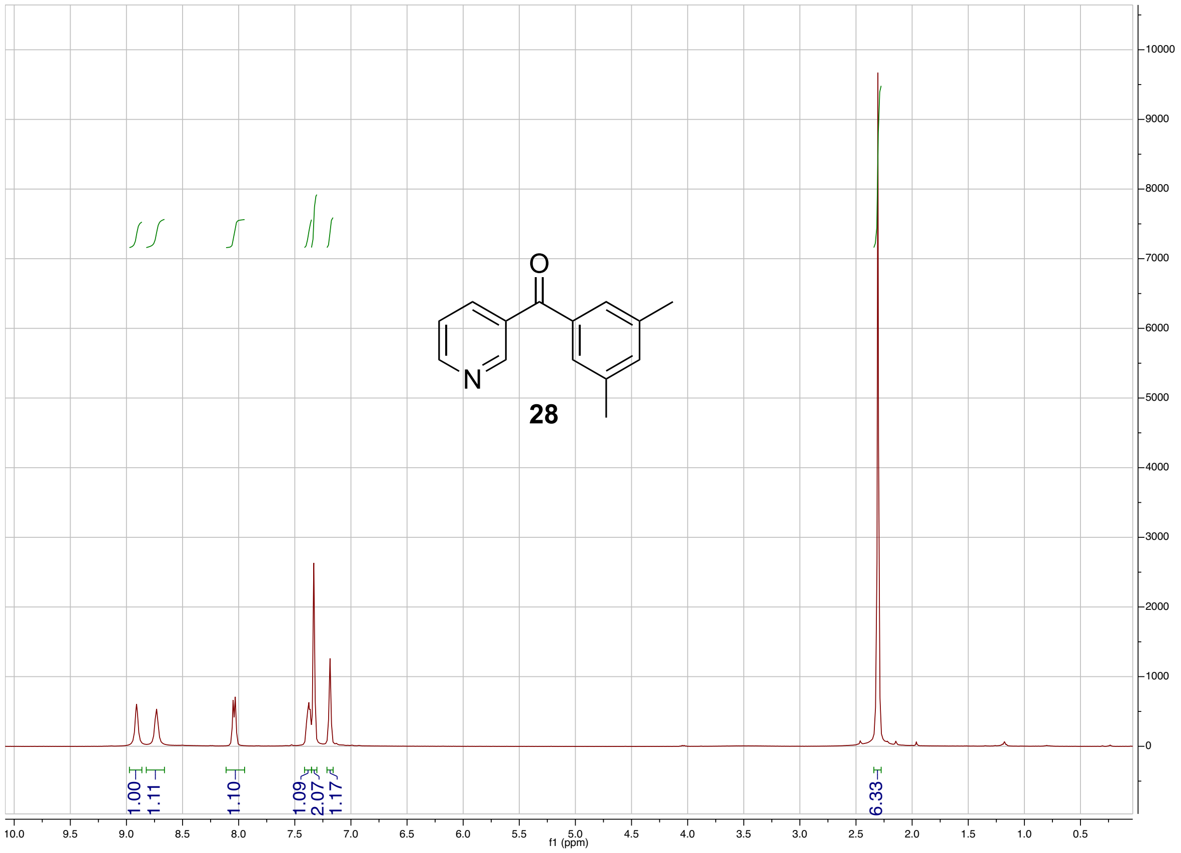

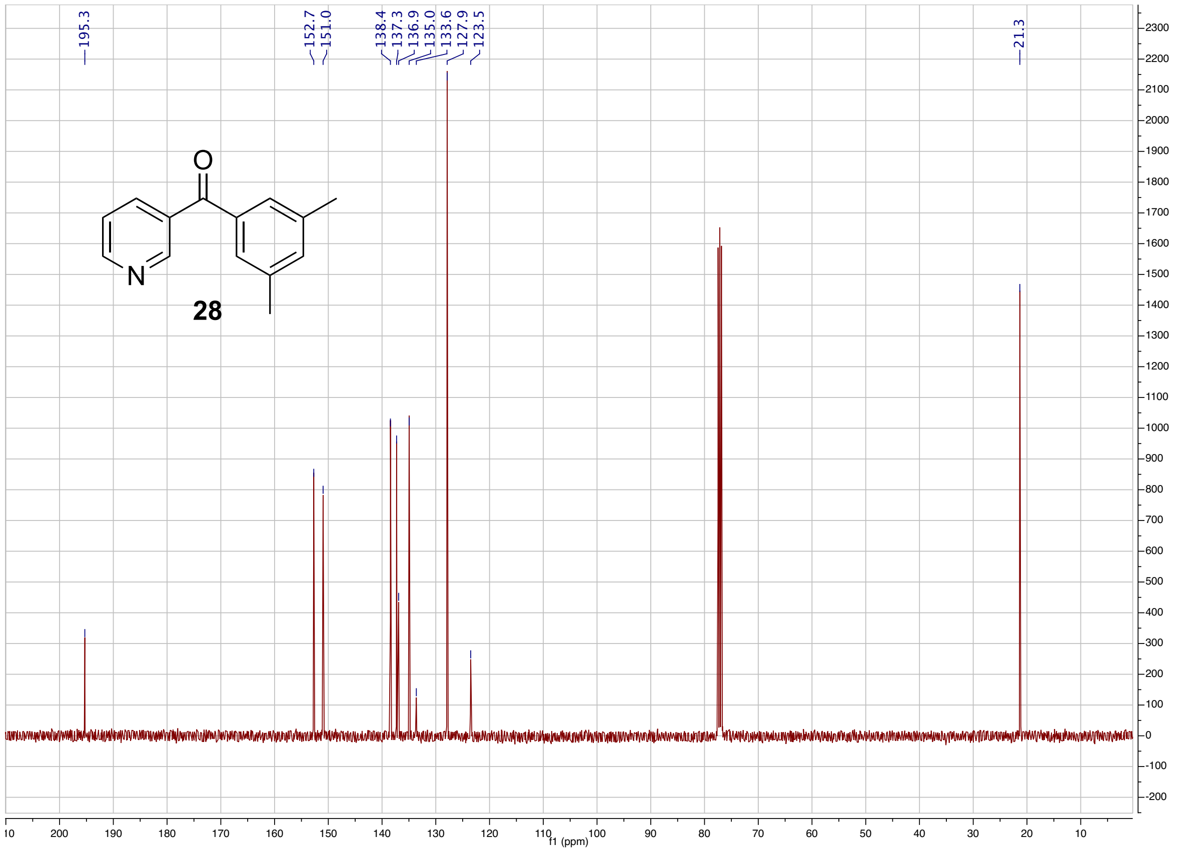

Supplementary Figure 26: ^{1}H and ^{13}C NMR of compound **28**.

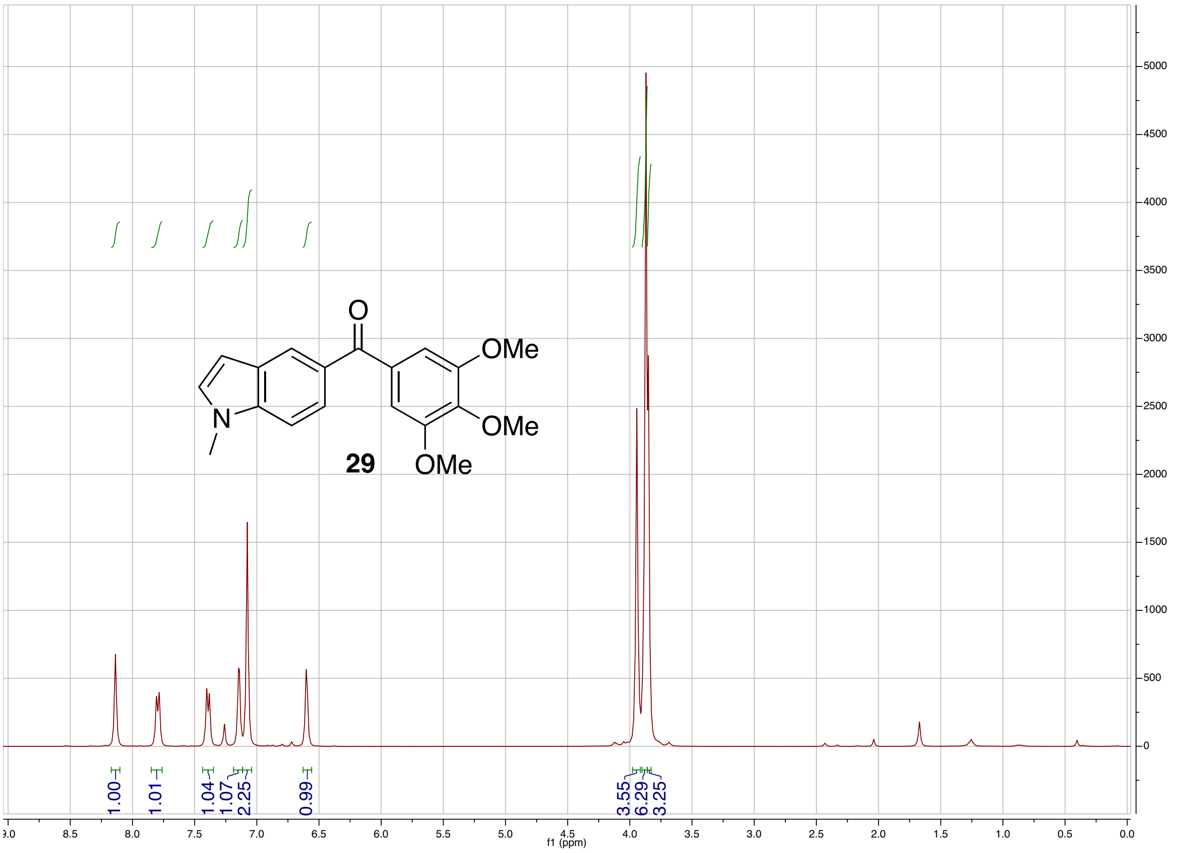

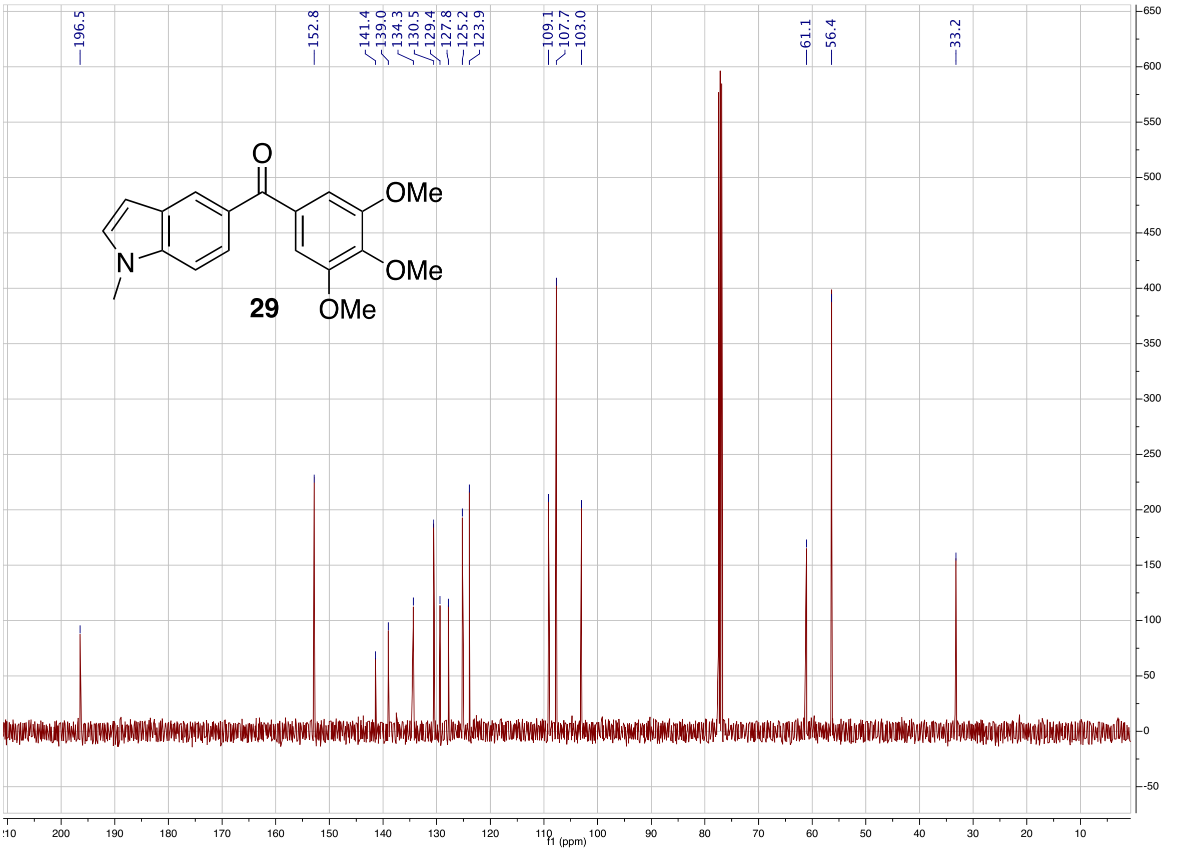

Supplementary Figure 27: ^{1}H and ^{13}C NMR of compound **29**.

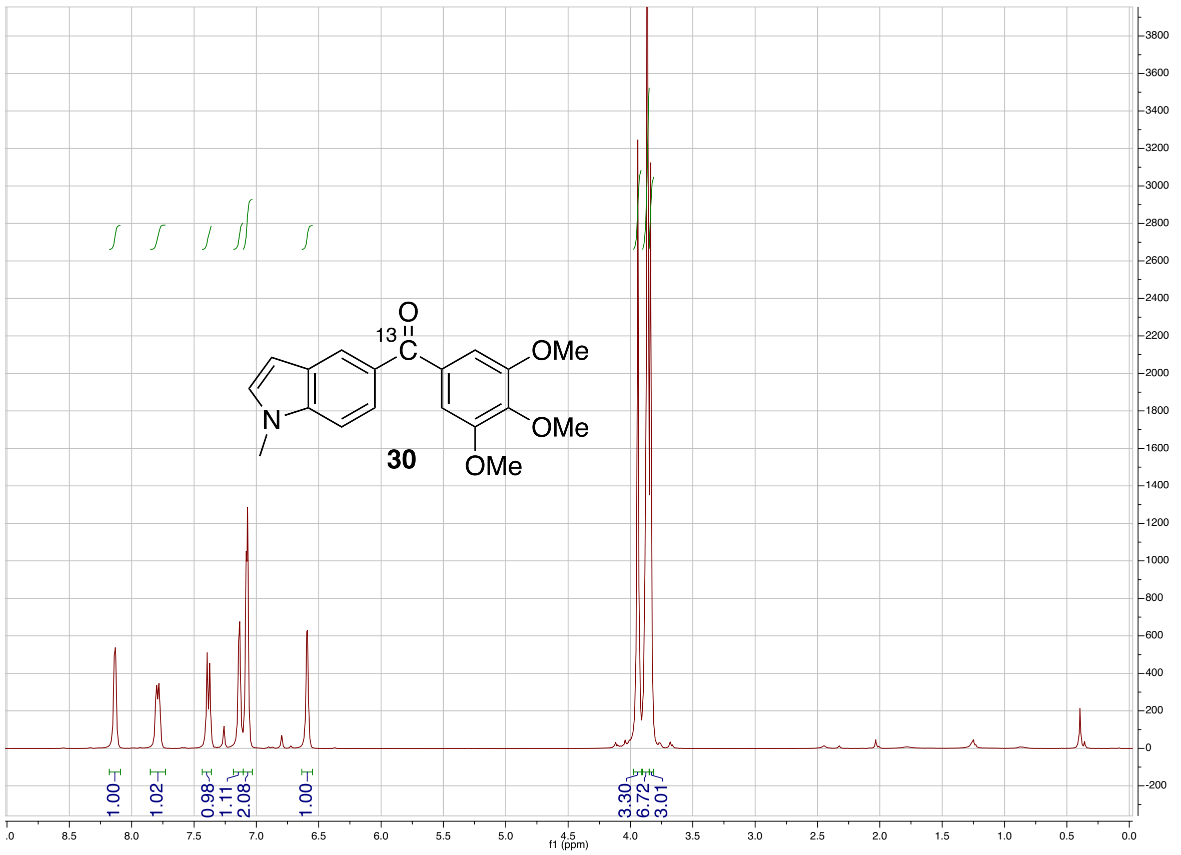

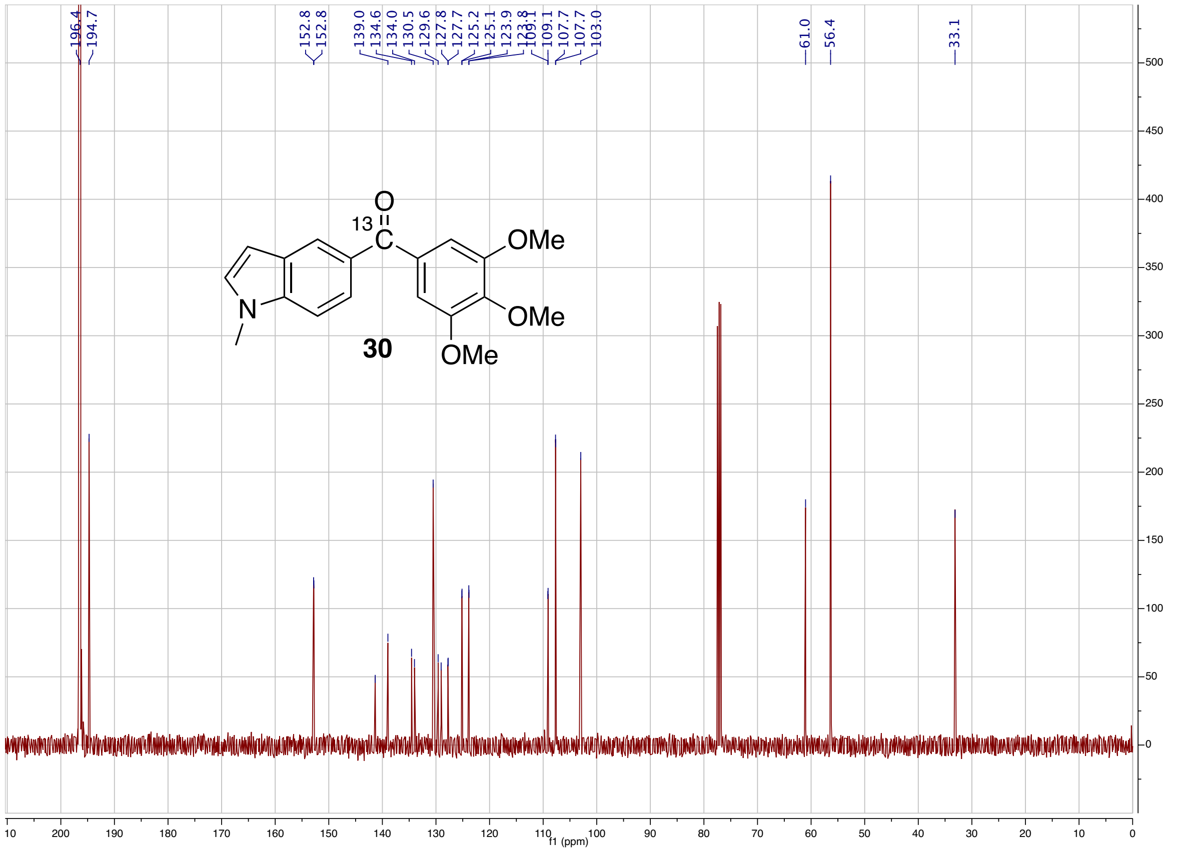

Supplementary Figure 28: ^{1}H and ^{13}C NMR of compound **30**.

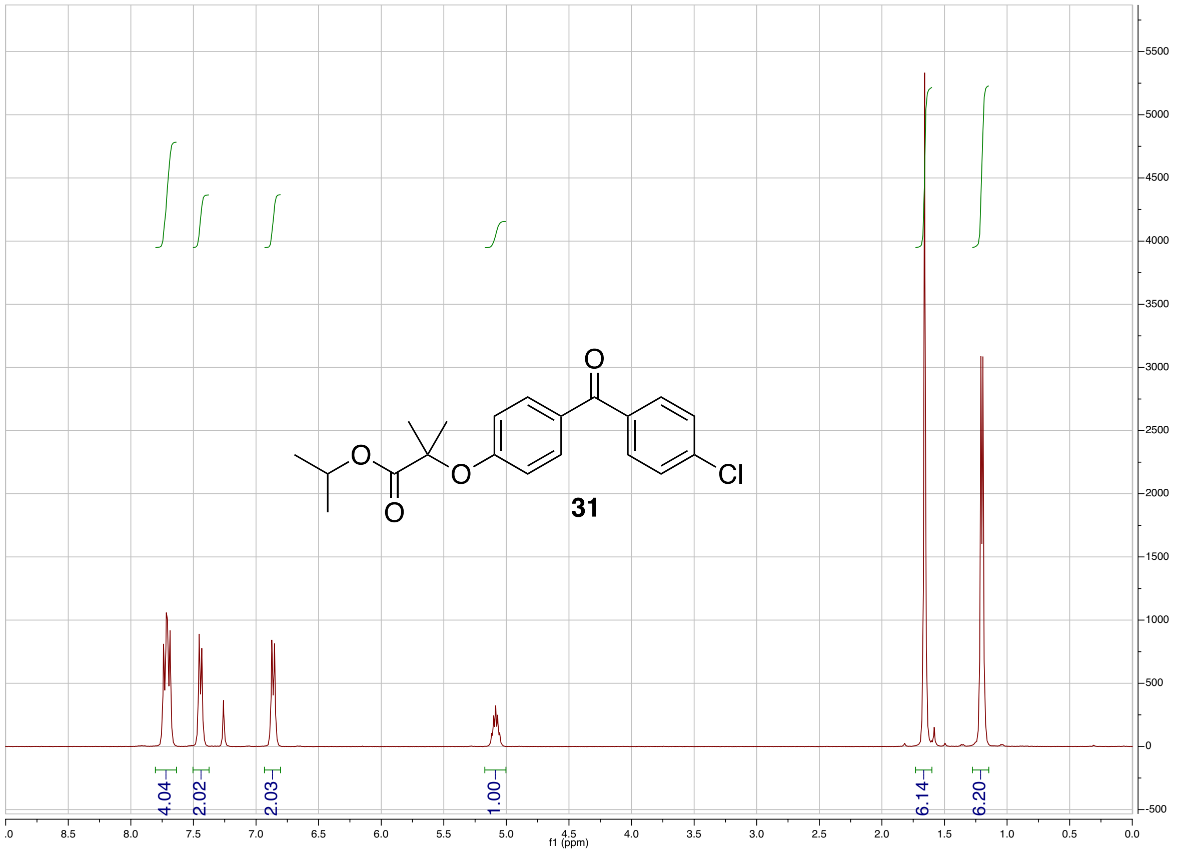

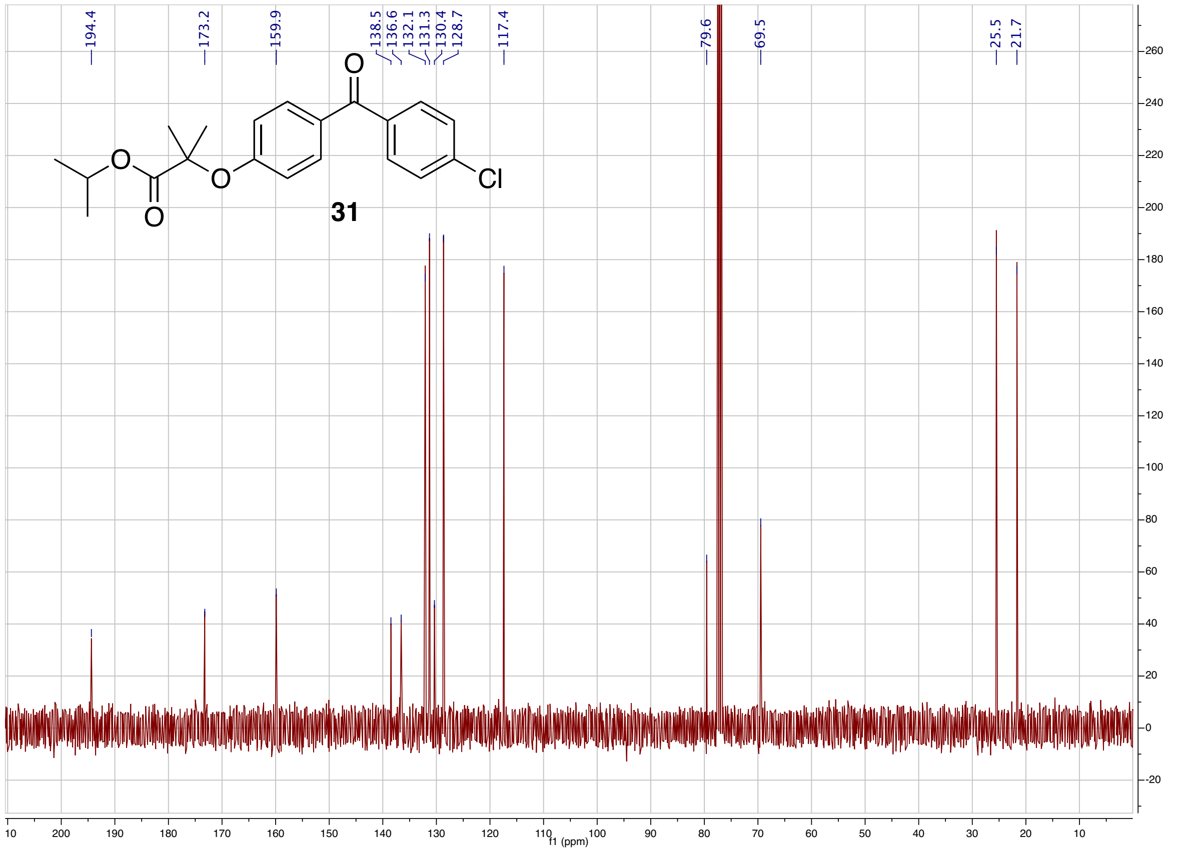

Supplementary Figure 29: ^{1}H and ^{13}C NMR of compound **31**.

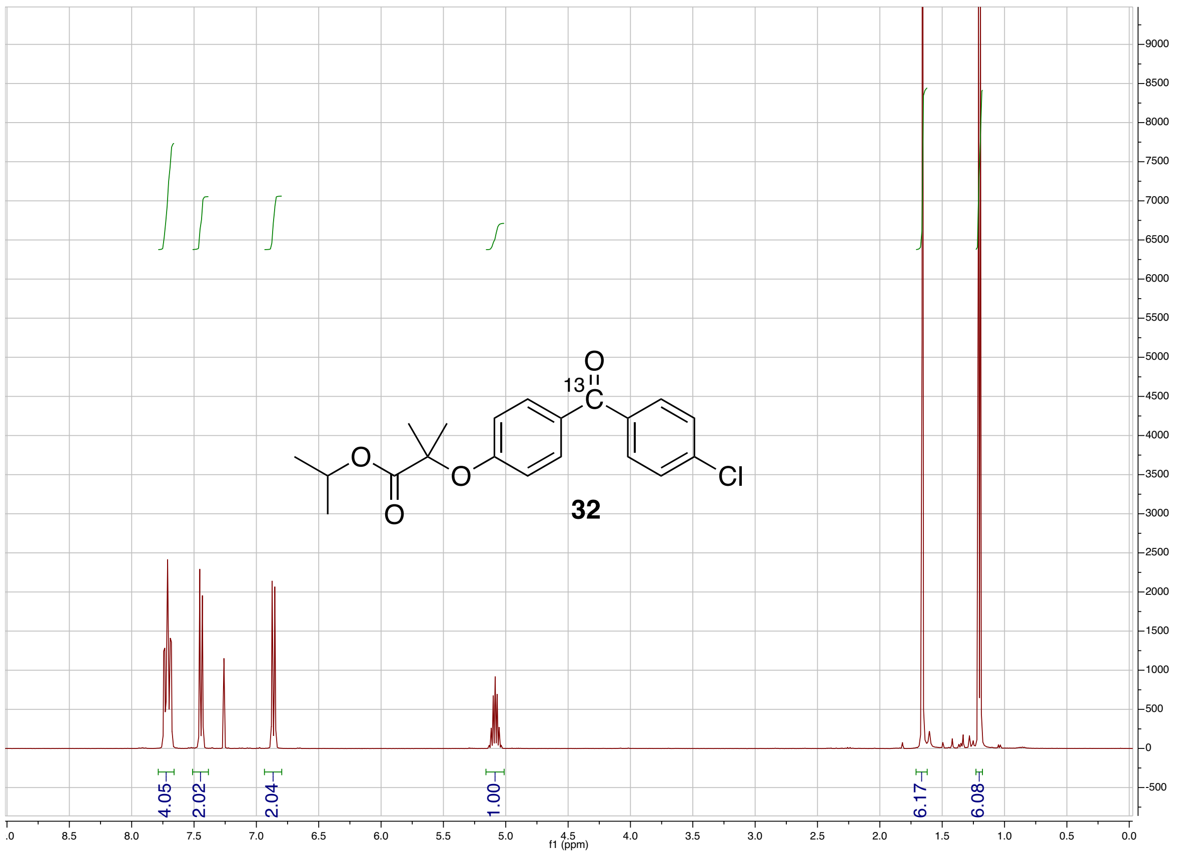

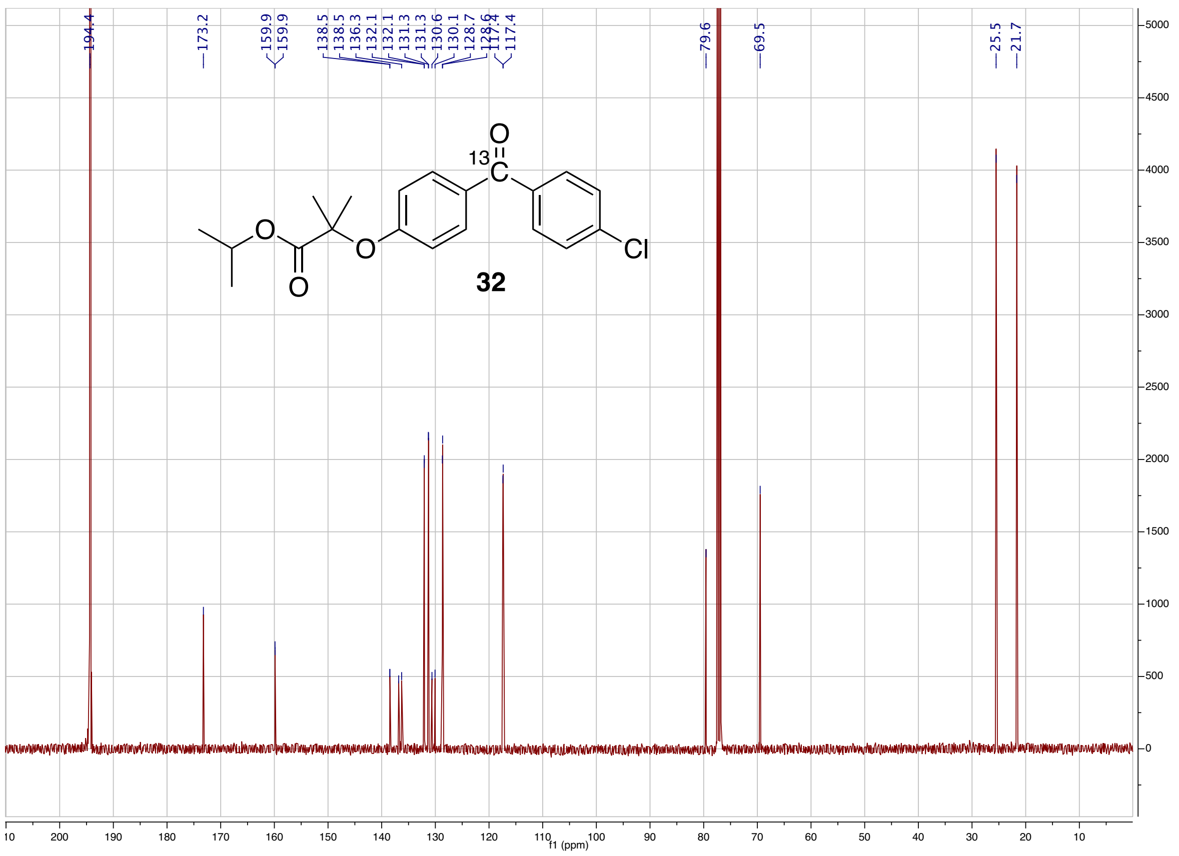

Supplementary Figure 30: ^{1}H and ^{13}C NMR of compound **32**.

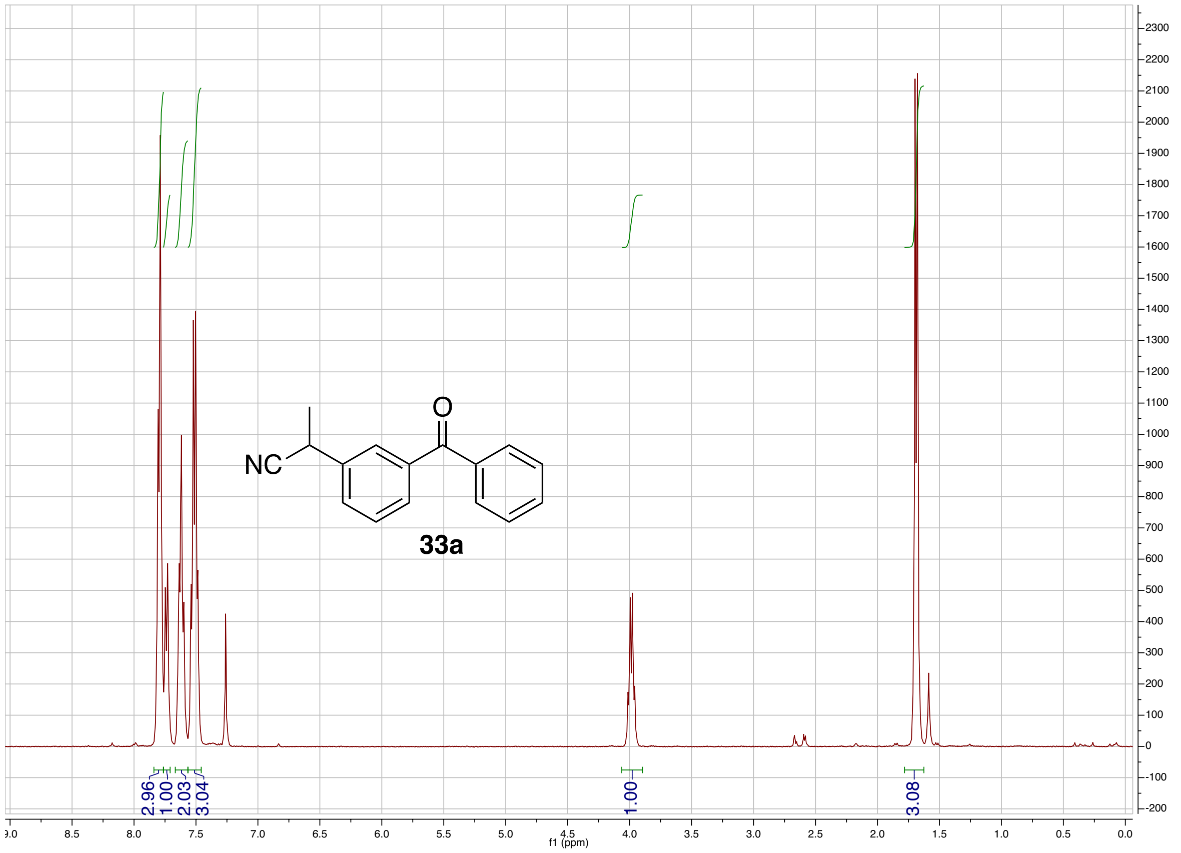

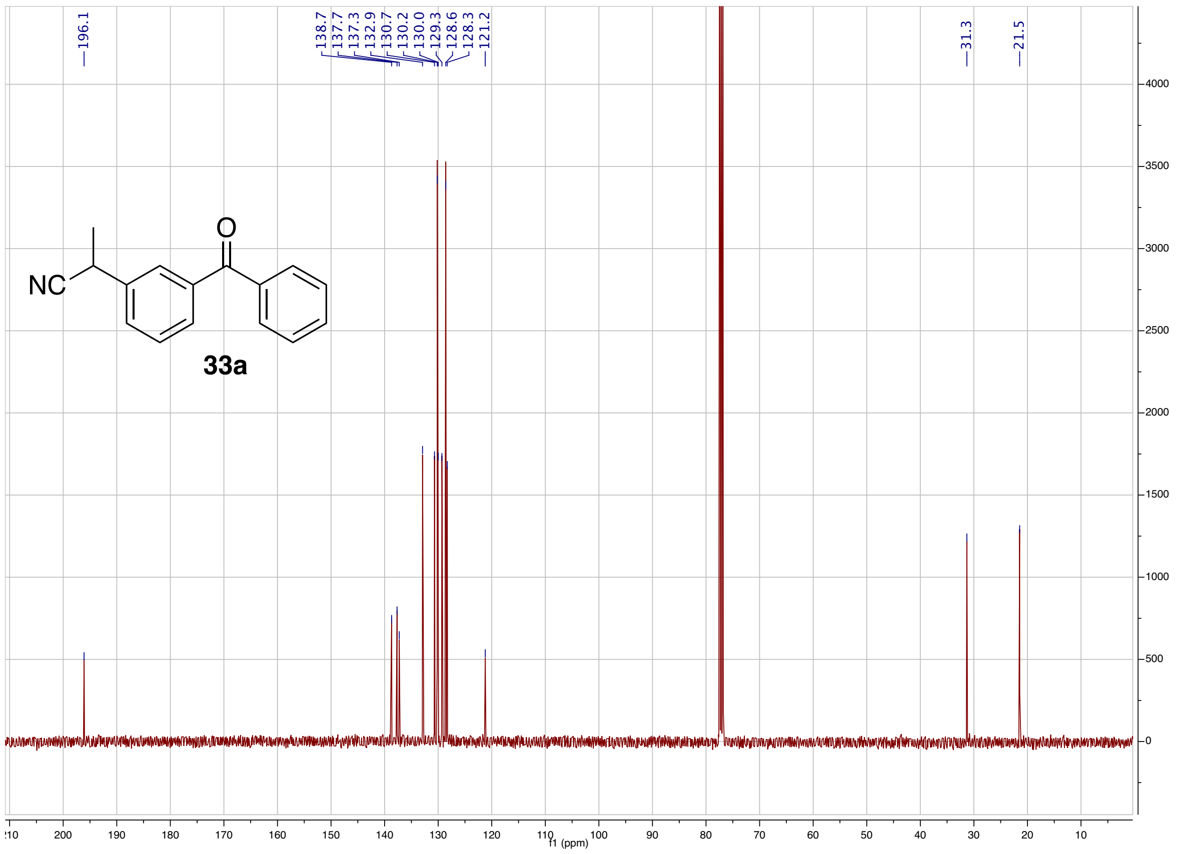

Supplementary Figure 31: ^{1}H and ^{13}C NMR of compound **33a**.

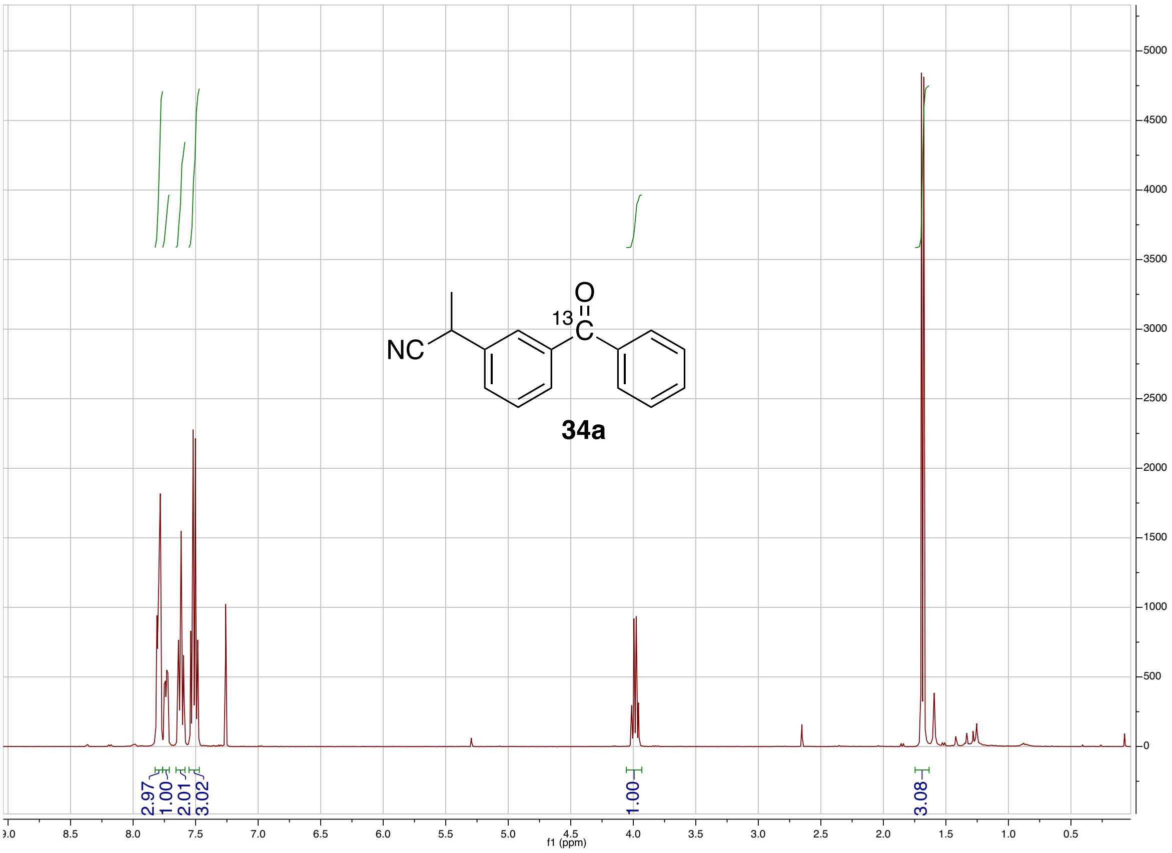

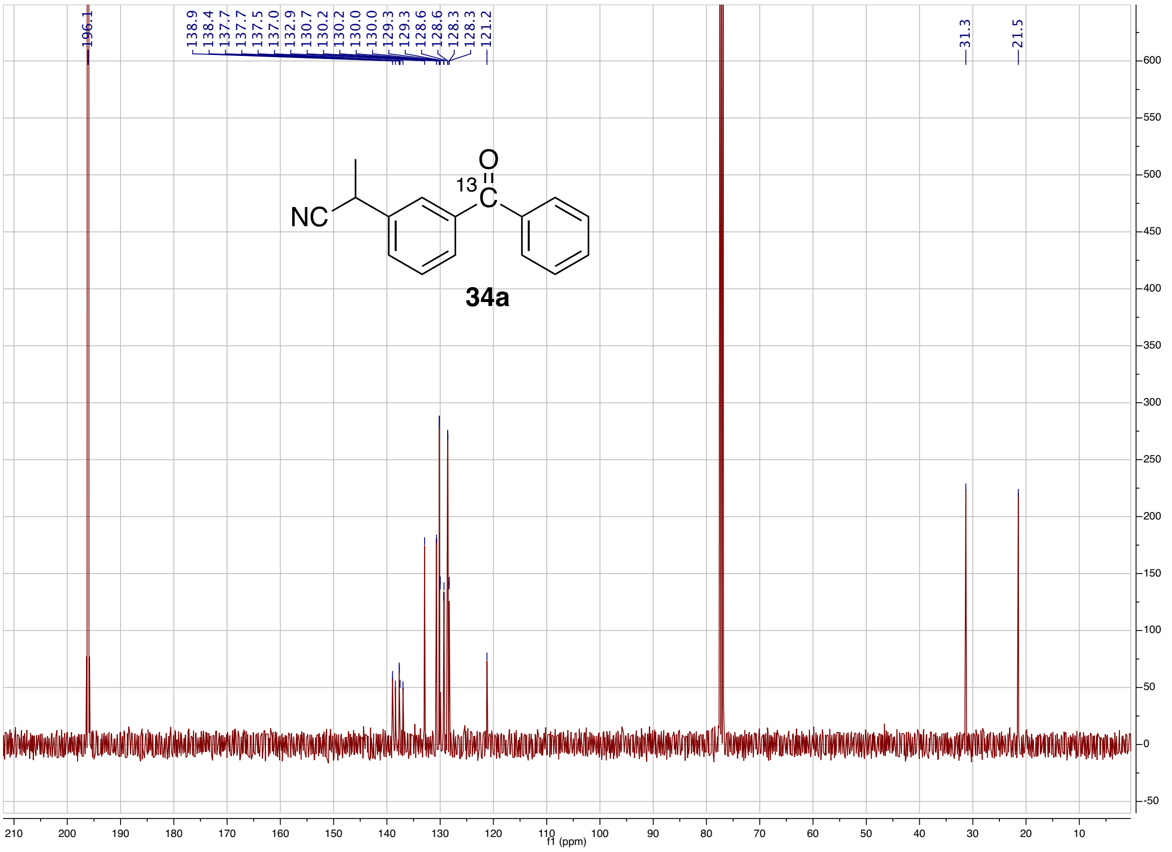

Supplementary Figure 32: ^{1}H and ^{13}C NMR of compound **34a**.

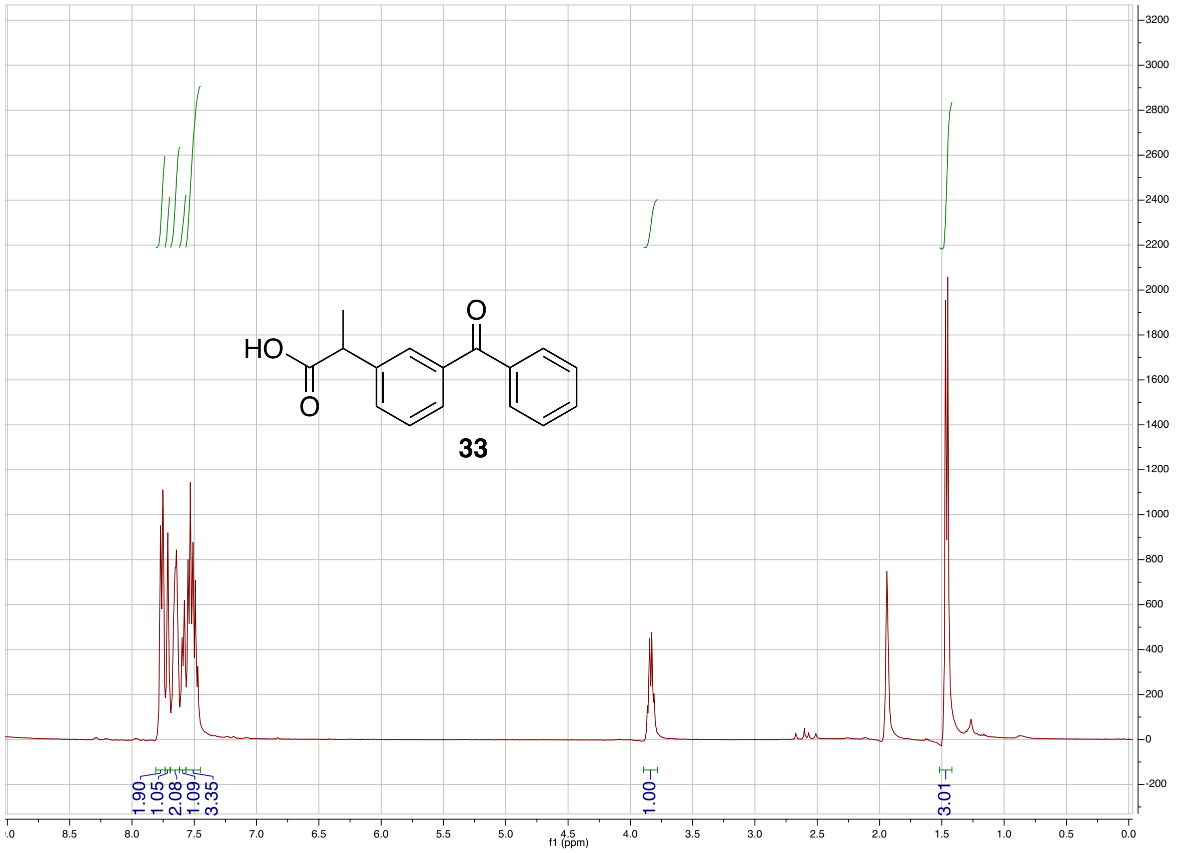

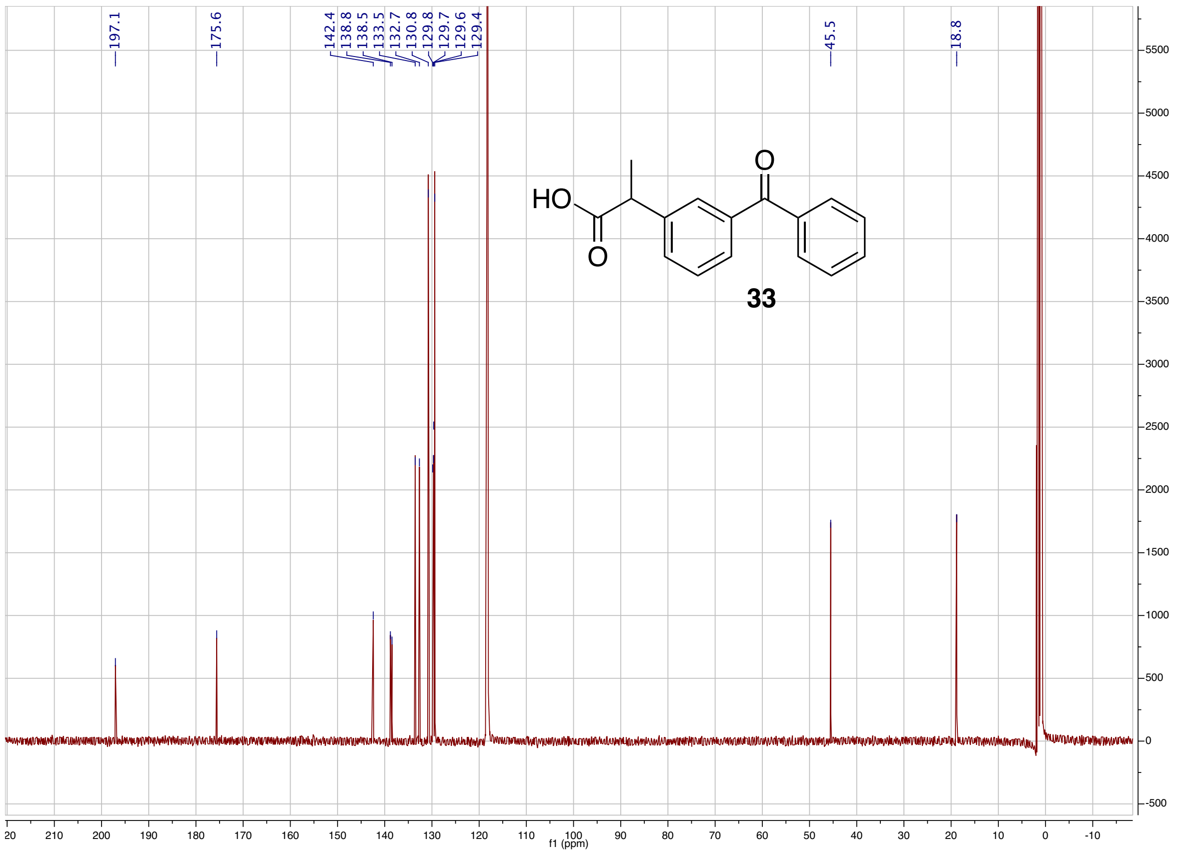

Supplementary Figure 33: ^{1}H and ^{13}C NMR of compound **33**.

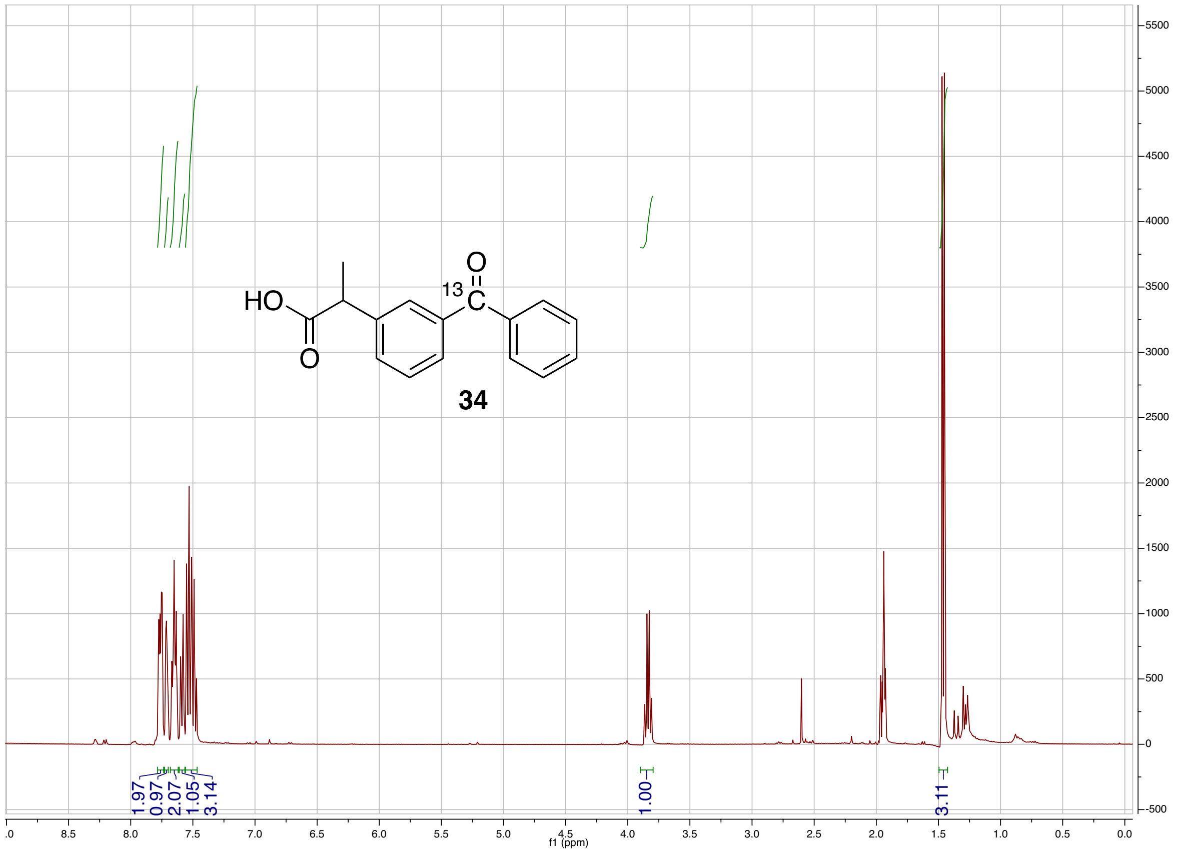

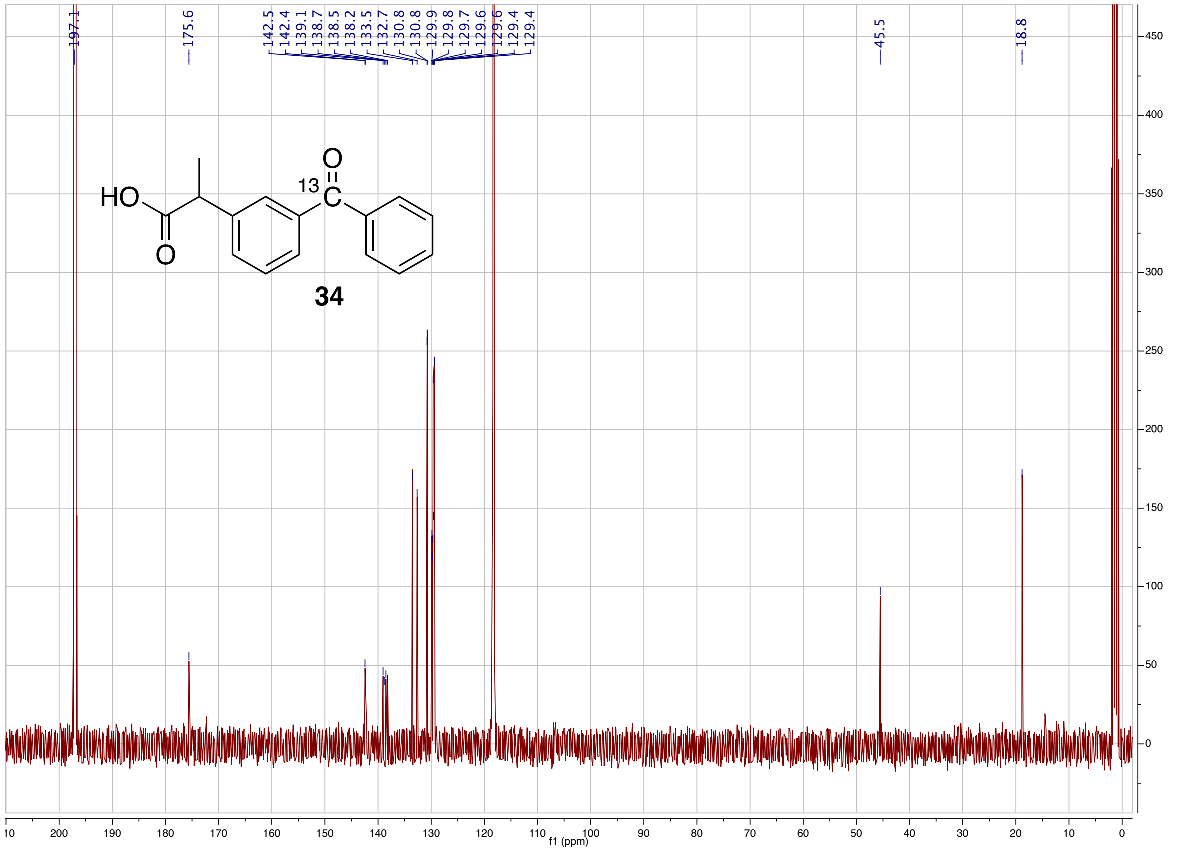

Supplementary Figure 34: ^{1}H and ^{13}C NMR of compound **34**.

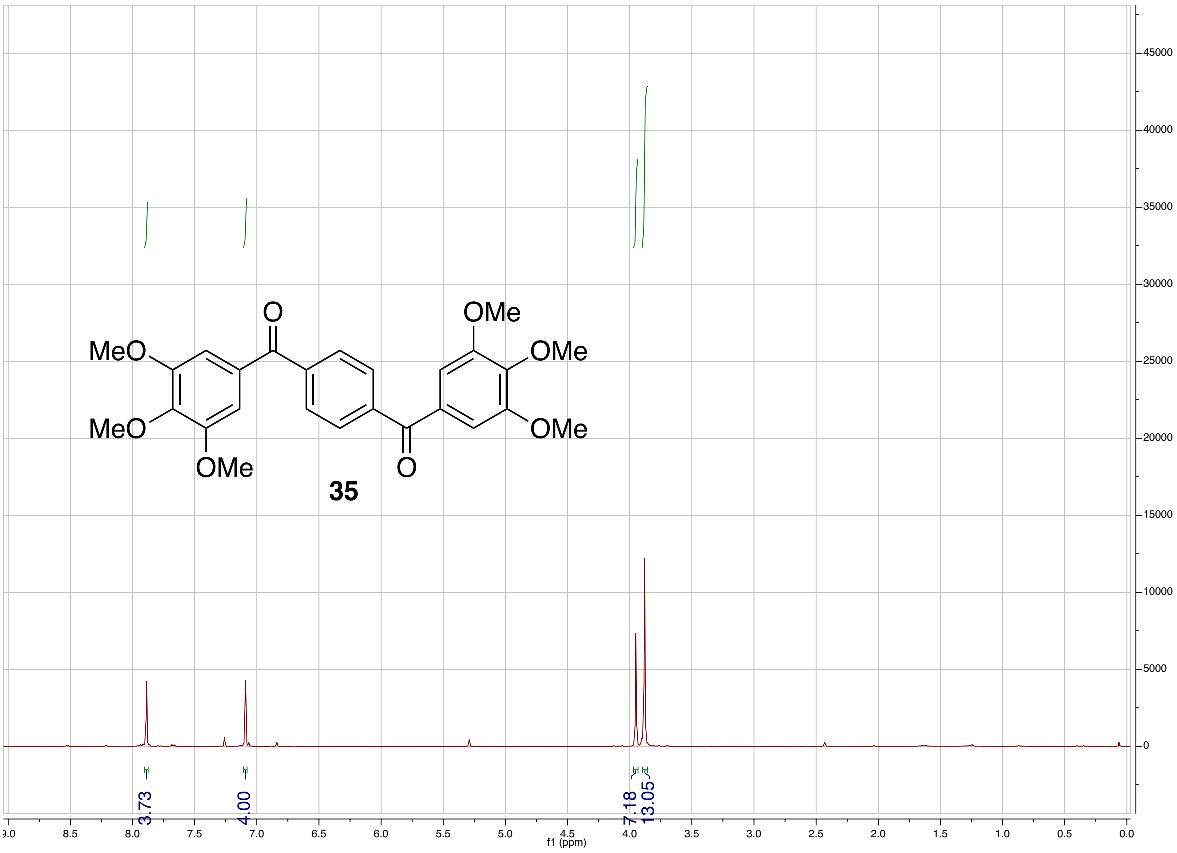

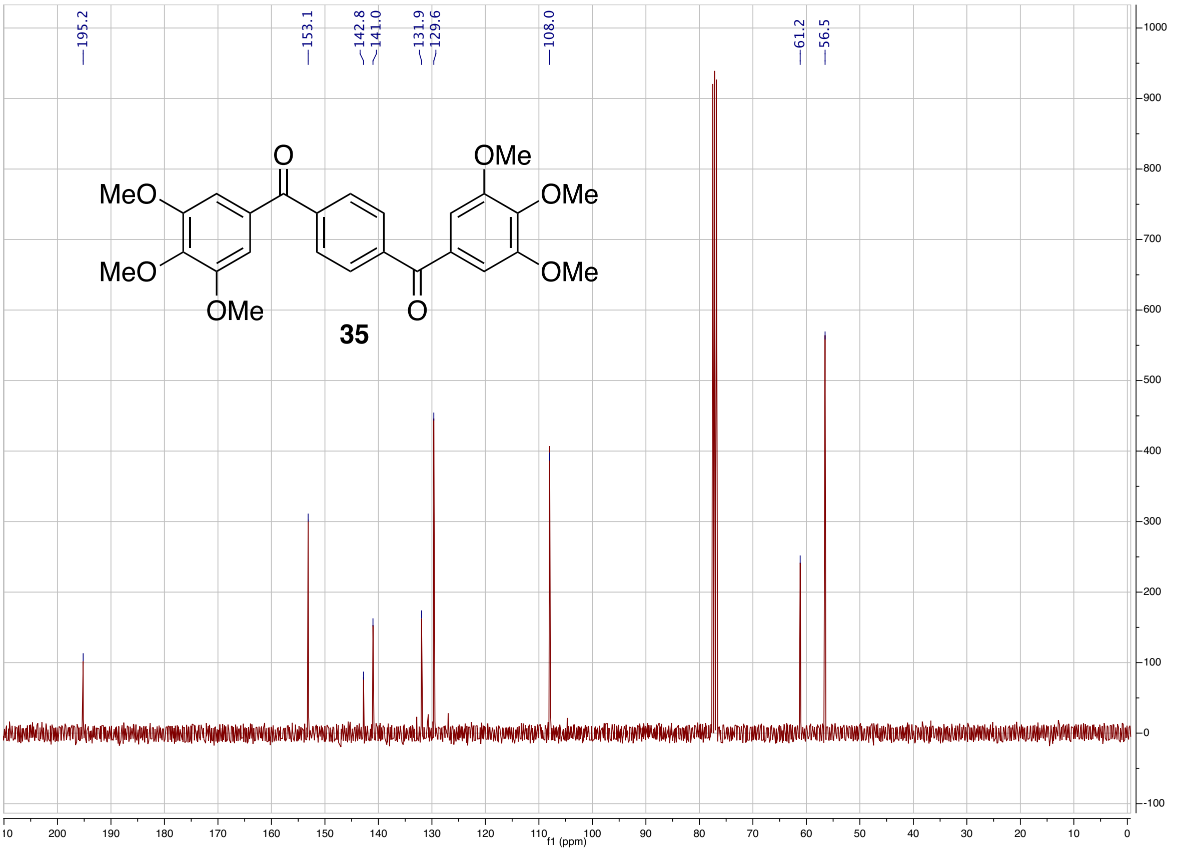

Supplementary Figure 35: ^{1}H and ^{13}C NMR of compound **35**.

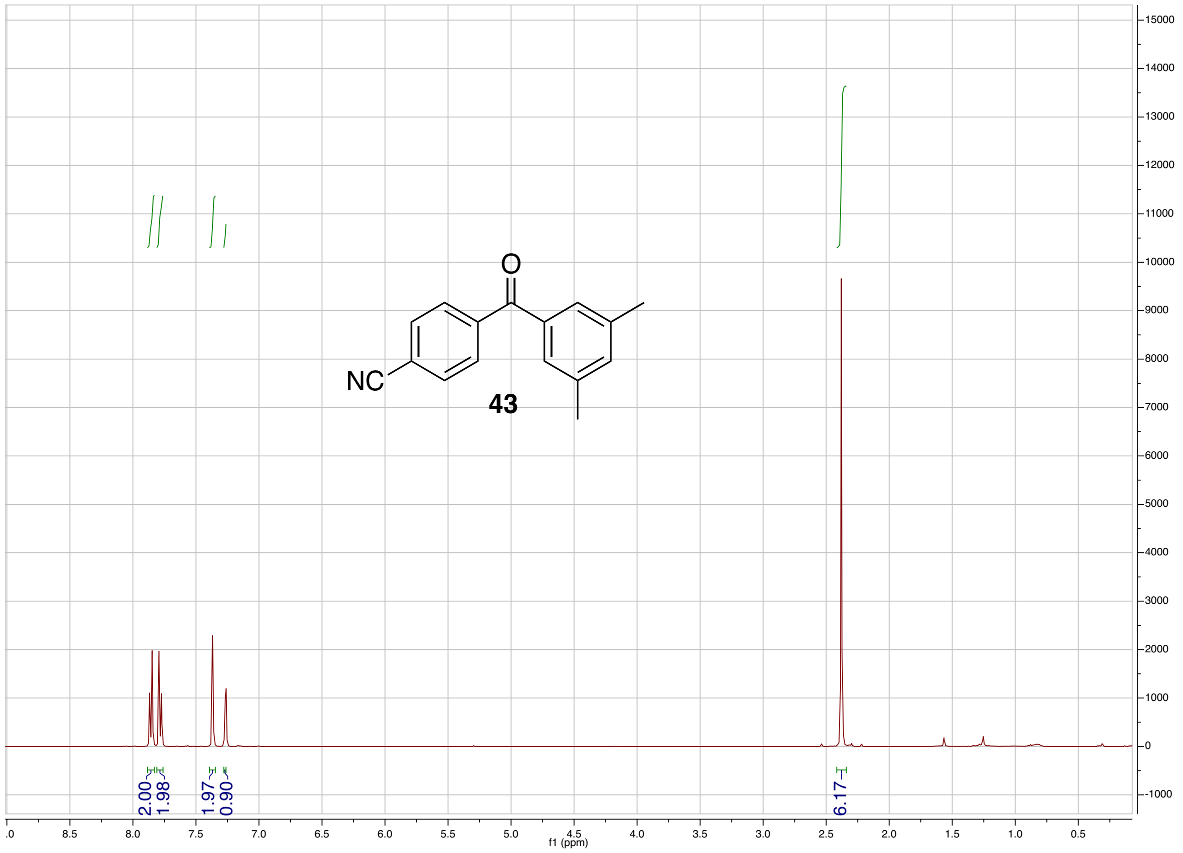

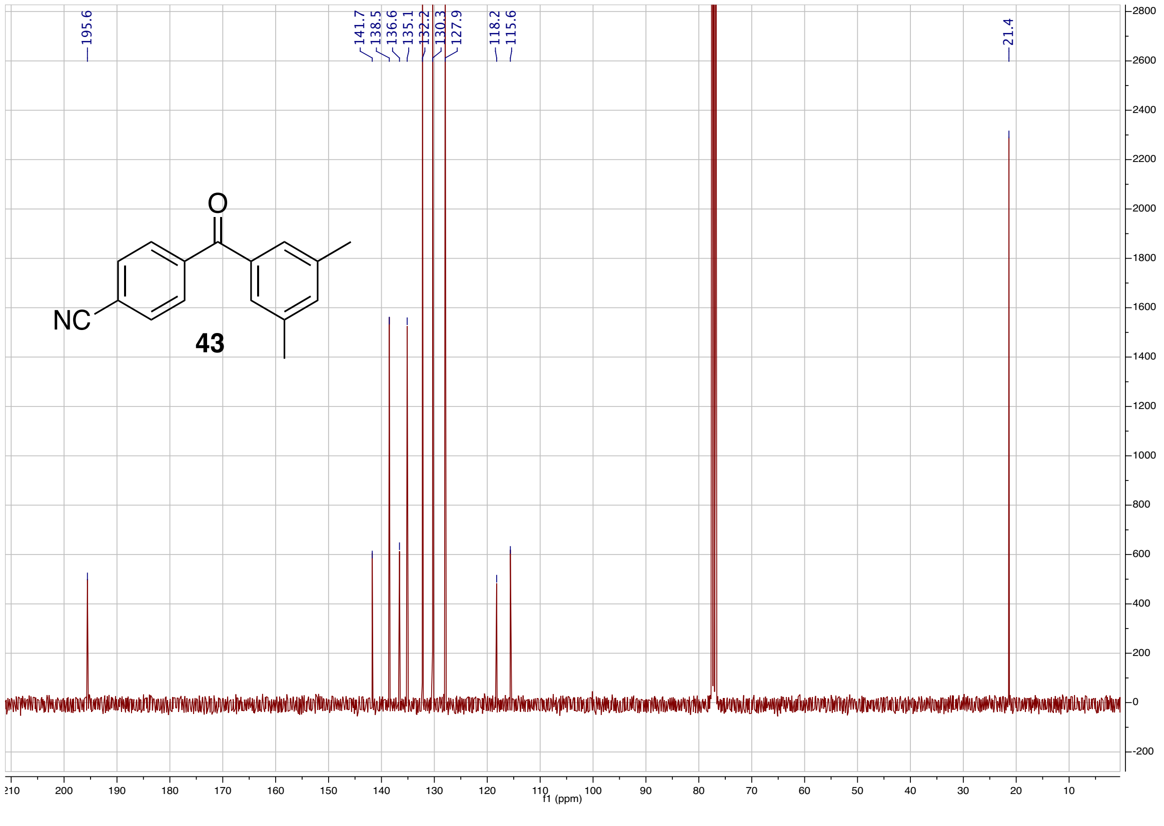

Supplementary Figure 36: ^{1}H and ^{13}C NMR of compound **43**.

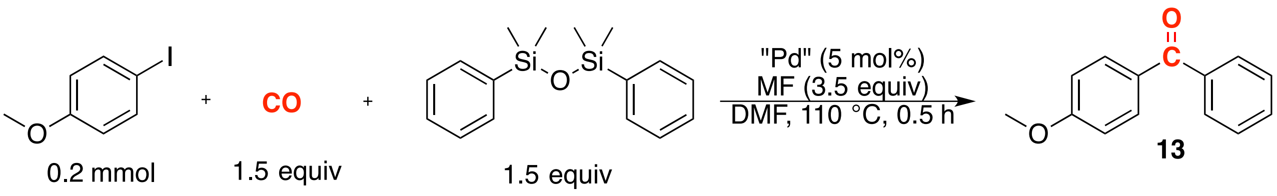

Entry	"Pd"	MF	Yield (**13**)
1	$Pd(dba)_2$	CsF	7%
2	$Pd(OAc)_2$	CsF	8%
3	$Pd(PPh_3)_4$	CsF	0
4	$(Pd(allyl)Cl)_2$	CsF	9%
5	$Pd(PPh_3)_2Cl_2$	CsF	7%
6	$PdCl_2$	CsF	14%
7	$[Pd(cinnamyl)Cl]_2$	CsF	9%
8	$Pd(acac)_2$	CsF	18%
9	$Pd(COD)Cl_2$	CsF	12%
10	$Pd(TFA)_2$	CsF	10%
11	$Pd(OPiv)_2$	CsF	6%
12	$Pd(CH_3CN)_2(OTs)_2$	CsF	7%
13	$PdBr_2$	CsF	15%
14	PdI_2	CsF	12%
15	Na_2PdCl_2	CsF	7%
16	$Pd(acac)_2$	LiF	0
17	$Pd(acac)_2$	NaF	0
18	$Pd(acac)_2$	KF	0
19	$Pd(acac)_2$	TBAF	0
20	$Pd(acac)_2$	AgF	13%

Supplementary Table 1: Screening of Pd- and fluoride sources.

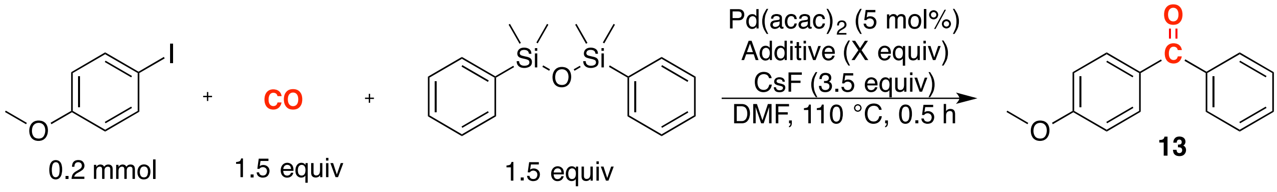

Entry	Additive	Yield (**13**)	Entry	Additive	Yield (**13**)
1	Cs_2CO_3 (1.0)	11%	18	Cu(MeSal) (0.1)	45%
2	K_2CO_3 (1.0)	3%	19	CuOAc (0.1)	30%
3	NaO*t*Bu (1.0)	0%	20	CuBr·DMSO (0.1)	39%
4	TMSOK (1.0)	0%	21	CuCN (0.1)	38%
5	Cy_2NMe (1.0)	16%	22	$Cu(CH_3CN)_4BF_4$ (0.1)	41%
6	Ag_2CO_3 (1.0)	21%	23	$[CuOTf]_2$·benzene (0.1)	39%
7	$PhCO_2Ag$ (1.0)	9%	24	CuF_2 (0.1)	24%
8	$AgNO_3$ (1.0)	16%	25	$Cu(CH_3CN)_4PF_6$ (0.1)	41%
9	Ag_2O (1.0)	29%	26	CuSCN (0.1)	10%
10	Ag_2O (0.1)	34%	27	$Cu(acac)_2$ (0.1)	40%
11	AgF (0.1)	21%	28	$Cu(OAc)_2$ (0.1)	27%
12	CuI (0.1)	32%	29	$CuBr_2$ (0.1)	28%
13	CuCl (0.1)	36%	30	$CuCl_2$ (0.1)	28%
14	CuBr (0.1)	40%	31	$CuSO_4$ (0.1)	14%
15	$Cu(CH_3CN)_4OTf$	40%	32	$Cu(TFA)_2$ (0.1)	22%
16	$CuBr(PPh_3)$(0.1)	10%	33	CuO (0.1)	17%
17	Cu_2O (0.1)	28%	34	$Cu(OTf)_2$ (0.1)	22%

Supplementary Table 2: Screening of additives.

Supplementary Table 3: Screening of stoichiometry and solvents.

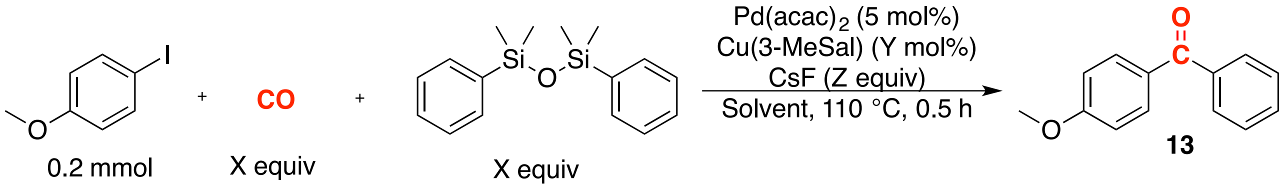

Entry	Cu(3-MeSal)	CsF	$CO/(PhSiMe_2O)_2$	Solvent	Yield (**13**)
1	1 mol%	3.5	1.5 equiv	DMF (2 mL)	25%
2	2 mol%	3.5	1.5 equiv	DMF (2 mL)	36%
3	5 mol%	3.5	1.5 equiv	DMF (2 mL)	40%
4	20 mol%	3.5	1.5 equiv	DMF (2 mL)	36%
5	50 mol%	3.5	1.5 equiv	DMF (2 mL)	25%
6	10 mol%	0.1	1.5 equiv	DMF (2 mL)	0%
7	10 mol%	0.5	1.5 equiv	DMF (2 mL)	0%
8	10 mol%	1.0	1.5 equiv	DMF (2 mL)	41%
9	10 mol%	1.5	1.5 equiv	DMF (2 mL)	45%
10	10 mol%	2.0	1.5 equiv	DMF (2 mL)	44%
11	10 mol%	3.0	1.5 equiv	DMF (2 mL)	45%
12	10 mol%	1.5	1.0 equiv	DMF (2 mL)	35%
13	10 mol%	1.5	1.2 equiv	DMF (2 mL)	40%
14	10 mol%	1.5	2.0 equiv	DMF (2 mL)	45%
15	10 mol%	1.5	2.5 equiv	DMF (2 mL)	28%
16	10 mol%	1.5	3.0 equiv	DMF (2 mL)	35%
17	10 mol%	1.5	1.5 equiv	Toluene (2 mL)	0%
18	10 mol%	1.5	1.5 equiv	Dioxane (2 mL)	0%
19	10 mol%	1.5	1.5 equiv	DMSO (2 mL)	0%
20	10 mol%	1.5	1.5 equiv	NMP (2 mL)	39%
21	10 mol%	1.5	1.5 equiv	Anisole (2 mL)	0
22	10 mol%	1.5	1.5 equiv	DMA (2 mL)	25%

23	10 mol%	1.5	1.5 equiv	HMPA (2 mL)	5%
24	10 mol%	1.5	1.5 equiv	DMF (1 mL)	33%
25	10 mol%	1.5	1.5 equiv	DMF (3 mL)	38%
26	10 mol%	1.5	1.5 equiv	DMF (4 mL)	7%

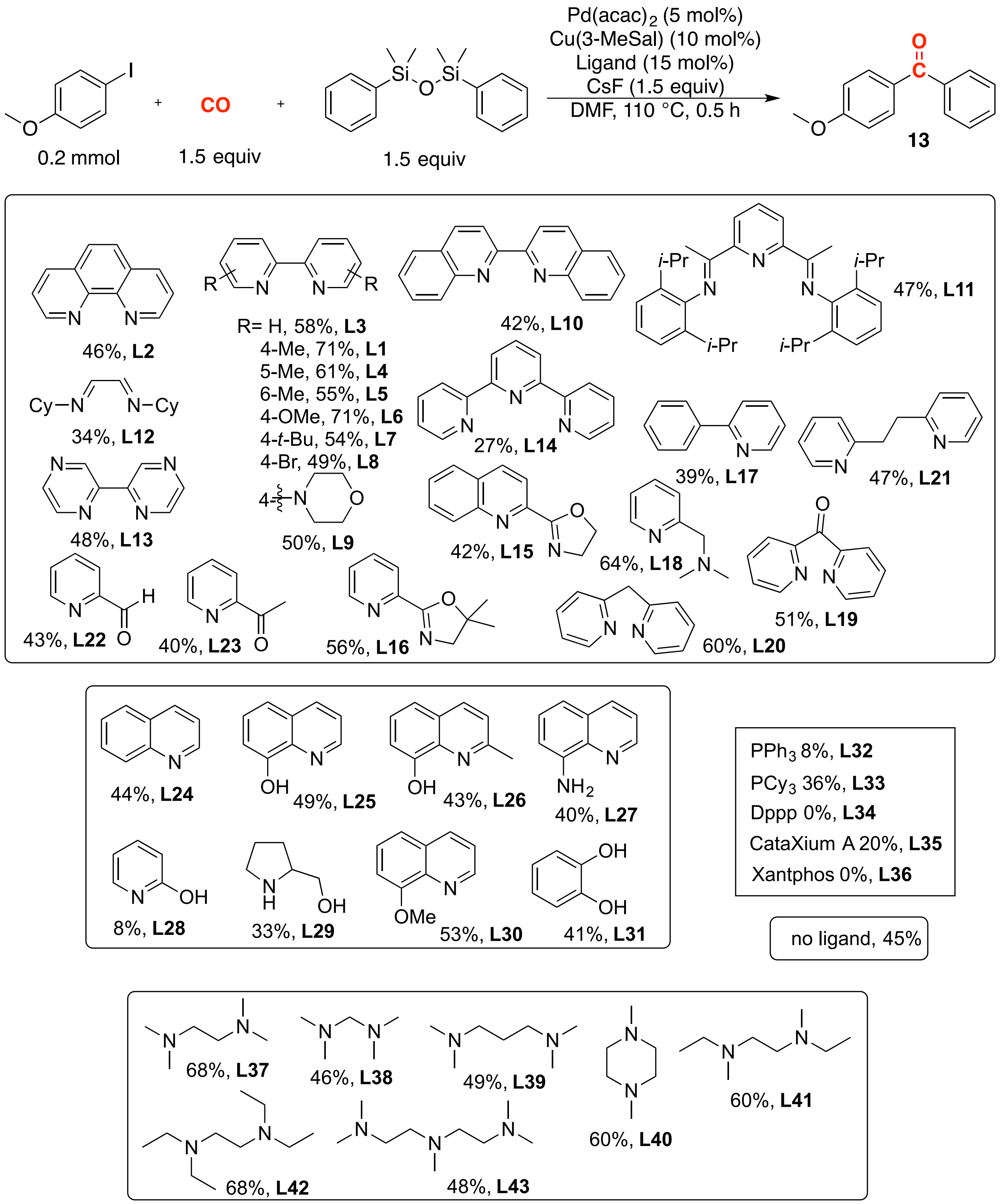

Supplementary Table 4: Screening of ligands.

Supplementary Note 1

All the carbonylative reactions using COgen and disiloxane were carried out in a two-chamber system (COware) and reactions using CO_2 and disilane were conducted in a COtube both in a glovebox under argon. All other chemicals were used as received without further purification. Disilanes and disiloxanes were prepared according to literature procedures (Fleming *et al.* *J. Chem. Soc., Perkin Trans. 1.* **1998**, *7*, 1209-1214). Solvents were dried according to standard procedures and flash chromatography was carried out on silica gel 60 (230-400 mesh). The chemical shifts are reported in ppm relative to solvent residual peak. The ^{1}H NMR spectra were recorded at 400 MHz, ^{13}C NMR spectra were recorded at 100 MHz, ^{19}F NMR spectra were recorded at 367 MHz on a Bruker 400 spectrometer. NMR spectra are reported as follows (s = singlet, d = doublet, t = triplet, q = quartet, quin = quintuplet, sext = sextet, sep = septet, m = multiplet, br = broad, dd = double doublet, dt = double triplet, ddd = double double doublet; coupling constant(s) in Hz; integration). HRMS spectra were recorded on a LC TOF (ES) apparatus.

COware and COtube

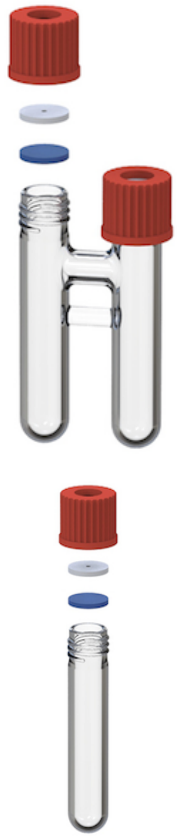

COware: Two glass vials (Chamber A and B) connected with a glass tube to allow gas-transfer.

Total volume = 9.0 mL (COtube) and 20.0 mL (COware).

The system is sealed using a screw cap and a Teflon® coated silicone seal.

Glassware under pressure - Warning!

- Glass equipment should always be examined for damages to its surface, which may weaken its strength.
- One must abide to all laboratory safety procedures and always work behind a shield when working with glass equipment under pressure.
- COware is pressure tested to 224 psi, but should under no circumstances be operated above 60 psi (5 bar).

Supplementary Methods

General procedure for the Carbonylative Hiyama-Denmark reaction using disilanes and CO_2

A flame-dried COtube charged with a stirring bar and disilane (0.3 mmol, 1.5 equiv) was transferred to an argon-filled glovebox. CsF (45.6 mg, 0.3 mmol, 1.5 equiv) and DMF (1.0 mL) was then added in that order. The COtube was sealed with a screwcap fitted with a Teflon seal and removed from the glovebox. CO_2 (7.2 mL, 0.3 mmol, 1.5 equiv) was then injected via syringe and the reaction mixture was stirred at the stated temperature for 1 h. Meanwhile, a stock solution (3 equivalents) was made which was used to provide dublicates of the reaction in the following manner: Aryl iodide (0.6 mmol, 3.0 equiv) and 4,4-dimethyl-2,2-bipyridine (16.5 mg, 0.090 mmol) were added to a flame-dried 8 mL vial charged with a stirring bar and transferred to an argon-filled glovebox together with two flame-dried 4 mL vials. $Pd(acac)_2$ (9.0 mg, 0.030 mmol) and copper(I) 3-methylsalicylate (12.9 mg, 0.060 mmol) were transferred to seperate 4 mL vials and dissolved in DMF (750 μL), respectively. The vials were briefly shaken to obtain a homogenous solution. DMF (1.5 mL) was added to the 8 mL vial followed by heating at 90 °C for 10 seconds. The vial was then removed from the heating block and the Pd- and Cu solutions were then added. The 8 mL vial was then capped with Teflon-containing screw-cap and removed from the glovebox. 1 mL of the stock solution was then injected to the COtube via syringe and reacted for 1 h. The reaction was cooled to room temperature, diluted and transferred with EtOAc to a 25 mL flask and the volatiles were removed *in vacuo*. The crude residue was subjected to flash column chromatography using pentane/ethyl acetate as eluent to afford the desired product. All yields are average of two runs.

General procedure for ^{13}C-labeling of the Carbonylative Hiyama-Denmark reaction using disilanes and $^{13}CO_2$

To chamber A of a flame-dried COware was added disilane (0.3 mmol, 1.5 equiv) and $Ba^{13}CO_3$ (158.7 mg, 0.8 mmol, 4.0 equiv) was added to chamber B. The loaded COware was transferred to an argon-filled glovebox, where CsF (45.6 mg, 0.3 mmol, 1.5 equiv) was added to chamber A followed by DMF (1.0 mL). The COware was sealed with screwcaps fitted with a Teflon seal and removed from the glovebox. Concentrated H_2SO_4 (4.0 mL) was injected into chamber B via syringe and chamber A was heated to the described temperature, whereas

chamber B was stirred at room temperature for 1 hour. Meanwhile, a stock solution (3 equivalents) was made which was used to provide dublicates of the reaction in the following manner: Aryl iodide (0.6 mmol, 3.0 equiv) and 4,4-dimethyl-2,2-bipyridine (16.5 mg, 0.090 mmol) were added to a flame-dried 8 mL vial charged with a stirring bar and transferred to an argon-filled glovebox together with two flame-dried 4 mL vials. $Pd(acac)_2$ (9.0 mg, 0.030 mmol) and copper(I) 3-methylsalicylate (12.9 mg, 0.060 mmol) were transferred to seperate 4 mL vials and dissolved in DMF (750 μL), respectively. The vials were briefly shaken to obtain a homogenous solution. DMF (1.5 mL) was added to the 8 mL vial followed by heating at 90 °C for 10 seconds. The vial was then removed from the heating block and the Pd- and Cu solutions were then added. The 8 mL vial was then capped with Teflon-containing screw-cap and removed from the glovebox. 1 mL of the stock solution was then injected to the COtube via syringe and reacted for 1 h. The reaction was cooled to room temperature, diluted and transferred with EtOAc to a 25 mL flask and the volatiles were removed *in vacuo*. The crude residue was subjected to flash column chromatography using pentane/ethyl acetate as eluent to afford the desired product. All yields are average of two runs.

General conditions for the Carbonylative Hiyama-Denmark coupling using disiloxanes and CO (Figure 4)

To chamber A of a flame-dried COware in an argon-filled glovebox was added aryl halide (0.2 mmol), diaryldisiloxane (0.3 mmol, 1.5 equiv), CsF (45.6 mg, 0.3 mmol, 1.5 equiv), $Pd(acac)_2$ (3.0 mg, 0.010 mmol, 5 mol%), copper(I) 3-methylsalicylate (4.3 mg, 0.020 mmol, 10 mol%), 4,4-dimethyl-2,2-bipyridine (5.5 mg, 0.030 mmol, 15 mol%) and DMF (2 mL). To chamber B was added $Pd(dba)_2$ (5.8 mg, 0.01 mmol), $HBF_4P(t\text{Bu})_3$ (5.8 mg, 0.02 mmol), COgen (81.6 mg, 0.3 mmol), Cy_2NMe (129 μL, 0.6 mmol) and DMF (2 mL). The two-chamber system was sealed with Teflon-containing screw caps and heated at the designated temperature for 1 hour. The reaction was cooled to room temperature, diluted and transferred with EtOAc to a 25 mL flask and the volatiles were removed *in vacuo*. The crude residue was subjected to flash column chromatography using pentane/ethyl acetate as eluent to afford the desired product. All yields are average of two runs.

Evaluating the role of the halide (Figure 4a)

Aryl bromide: 4-Bromobenzonitrile (36.4 mg, 0.2 mmol) was employed using the general conditions for disiloxanes and CO at 110 °C for 18 hours. Omitting KI in chamber A did not provide any formation of biarylketone **42**. Adding KI (49.8 mg, 0.3 mmol) allowed **42** to be isolated in a 22% yield after flash column chromatography. Both reactions were run twice.

Aryl iodide: 4-Iodobenzonitrile (45.8 mg, 0.2 mmol) was employed using the general conditions for disiloxanes and CO at 80 °C for 18 hours. Adding KBr (35.7 mg, 0.3 mmol) to chamber A did not provide any formation of biarylketone **42**. Omitting KBr allowed **42** to be isolated in a 70% yield after flash column chromatography. Both reactions were run twice.

Competition studies using different disiloxanes (Figure 4b-c)

Steric effect: 4-Iodoanisole (46.8 mg, 0.2 mmol) was used with 1,1,3,3-tetramethyl-1,3-di-*o*-tolyldisiloxane (94.4 mg, 0.3 mmol) and 1,1,3,3-tetramethyl-1,3-di-*p*-tolyldisiloxane (94.4 mg, 0.3 mmol) using the general conditions for disiloxanes and CO. The biarylketones **4** (37%) and **5** (19%) formed from both disiloxanes were isolated after column chromatography. Yields are an average of two runs.

Electronic effect: 4-Iodoanisole (46.8 mg, 0.2 mmol) was used with 1,1,3,3-tetramethyl-1,3-bis(4-(trifluoromethyl)phenyl)disiloxane (126.8 mg, 0.3 mmol) and 1,3-bis-(4-methoxyphenyl)-1,1,3,3-tetramethyldisiloxane (104.0 mg, 0.3 mmol) using the general conditions for disiloxanes and CO. The biarylketones **6** (18%) and **8** (35%) formed from both disiloxanes were isolated after column chromatography. Yields are an average of two runs.

Reaction profile – Steric/electronic effect of the disilane (Figure 4b-c)

Nine identical reactions were set up following the general procedure for disilanes and CO_2 using a stock solution based on 4-iodoanisole (468 mg, 2.0 mmol, 10 equiv) and a stock solution the disilane (3.0 mmol, 10 equiv). After addition of the mixture containing aryl iodide after 1 hour the reactions were quenched after 5, 10, 15, 20, 25, 30, 40, 50 and 60 minutes, respectively. The GC-yield of the reactions was plotted as a function of time. All experiments are average of two runs. All reactions were carried out at 110 °C, however, the

CO_2 reduction using the *p*-CF_3-diaryldisilane was conducted at room temperature for 1 h, due to instability of the corresponding disiloxane. The ensuing coupling reaction was conducted at 110 °C.

Characterization of products

(3,5-Dimethylphenyl)(4-methoxyphenyl)methanone (compound 3)

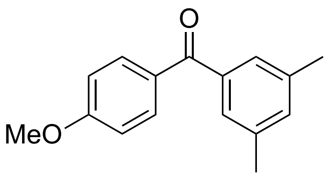

Reaction with 4-iodoanisole (46.8 mg) and 1,2-bis(3,5-dimethylphenyl)-1,1,2,2-tetramethyldisilane (98.0 mg) using the general procedure at 110 °C followed by flash columm chromatography using pentane/EtOAc (30:1) as eluent resulted in 38 mg (79%) of the title compound as a colorless solid. ^{1}H NMR (400 MHz, $CDCl_3$) δ 7.82 (d, J = 8.8 Hz, 2 H), 7.35 (s, 2 H), 7.19 (s, 1 H), 6.96 (d, J = 8.8 Hz, 2 H), 3.88 (s, 3 H), 2.37 (s, 6 H). ^{13}C NMR (100 MHz, $CDCl_3$) δ 195.9, 163.1, 138.4, 137.8 (2 C), 133.5, 132.5 (2 C), 130.4, 127.4 (2 C), 113.4 (2 C), 55.4, 21.2 (2 C). HRMS $C_{16}H_{16}O_2$ $[M+H]^+$; calculated 241.1223, found: 241.1227.

(4-Methoxyphenyl)(*p*-tolyl)methanone (compound 4)

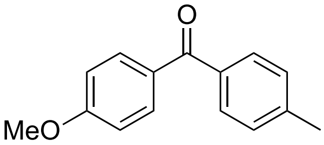

Reaction with 4-iodoanisole (46.8 mg) and 1,1,2,2-tetramethyl-1,2-di-*p*-tolyldisilane (89.6 mg) using the general procedure at 110 °C followed by flash columm chromatography using pentane/EtOAc (20:1) as eluent resulted in 33 mg (72%) of the title compound as a colorless solid. ^{1}H NMR (400 MHz, $CDCl_3$) 7.69 (d, J = 8.8 Hz, 2 H), 7.56 (d, J = 8.0 Hz, 2 H), 7.15 (d, J = 8.0 Hz, 2 H), 6.84 (d, J = 8.8 Hz, 2 H), 3.76 (s, 3 H), 2.32 (s, 3 H). ^{13}C NMR (100 MHz, $CDCl_3$) δ 195.4, 163.1, 142.7, 135.6, 132.5 (2 C), 130.6, 130.1 (2 C), 129.0 (2 C), 113.6 (2 C), 55.6, 21.7. HRMS $C_{15}H_{14}O_2$ $[M+H]^+$; calculated 227.1067, found: 227.1069.

(4-Methoxyphenyl)(*o*-tolyl)methanone (compound 5)

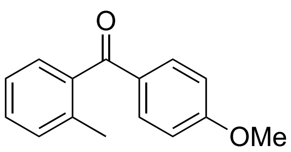

A: Reaction with 1-iodo-2-methylbenzene (43.6 mg) and 1,2-bis(4-methoxyphenyl)-1,1,2,2-tetramethyldisilane (99.2 mg) using the general procedure at 110 °C followed by flash columm chromatography using pentane/EtOAc (30:1) as eluent resulted in 24 mg (53%) of the title compound as a colorless oil. **B:** Reaction with 4-iodoanisole (46.8 mg) and 1,1,2,2-

tetramethyl-1,2-di-*o*-tolyldisilane (89.6 mg) using the general procedure at 110 °C followed by flash columm chromatography using pentane/EtOAc (25:1) as eluent resulted in 21 mg (47%) of the title compound as a colorless oil. ^{1}H NMR (400 MHz, $CDCl_3$) δ 7.76 (d, J = 8.8 Hz, 2 H), 7.33 (m, 1 H), 7.21 (m, 3 H), 6.89 (d, J = 8.8 Hz, 2 H), 3.84 (s, 3 H), 2.27 (s, 3 H). ^{13}C NMR (100 MHz, $CDCl_3$) δ 197.3, 163.7, 139.2, 136.1, 132.5 (2 C), 130.8, 130.5, 129.7, 127.9, 125.1, 113.7 (2 C), 55.5, 19.7. HRMS $C_{15}H_{14}O_2$ $[M+H]^+$; calculated 227.1067, found: 227.1068.

bis(4-Methoxyphenyl)methanone (compound 6)

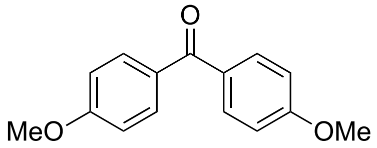

Reaction with 4-iodoanisole (46.8 mg) and 1,2-bis(4-methoxyphenyl)-1,1,2,2-tetramethyldisilane (99.2 mg) using the general procedure at 110 °C followed by flash columm chromatography resulted in 31 mg (65%) of the title compound as a colorless solid. ^{1}H NMR (400 MHz, $CDCl_3$) δ 7.79 (d, J = 8.8 Hz, 4 H), 6.96 (d, J = 8.8 Hz, 4 H), 3.88 (s, 6 H). ^{13}C NMR (100 MHz, $CDCl_3$) δ 194.6, 163.0 (2 C), 132.3 (4 C), 130.9 (2 C), 113.6 (4 C), 55.6 (2 C). HRMS $C_{15}H_{14}O_3$ $[M+H]^+$; calculated 243.1016, found: 243.1018.

(4-Fluorophenyl)(4-methoxyphenyl)methanone (compound 7)

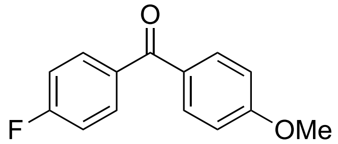

A: Reaction with 1-fluoro-4-iodobenzene (44.4 mg) and 1,2-bis(4-methoxyphenyl)-1,1,2,2-tetramethyldisilane (99.2 mg) using the general procedure at 100 °C followed by flash columm chromatography using pentane/EtOAc (30:1) as eluent resulted in

32 mg (70%) of the title compound as a colorless solid. **B:** Reaction with 4-iodoanisole (46.8 mg) and 1,2-bis(4-fluorophenyl)-1,1,2,2-tetramethyldisilane (92.0 mg) using the general procedure at 100 °C followed by flash columm chromatography using pentane/EtOAc (30:1) as eluent resulted in 28 mg (61%) of the title compound as a colorless solid. ^{1}H NMR (400 MHz, $CDCl_3$) δ 7.80 (dt, J = 2.4, 8.8 Hz, 4 H), 7.15 (t, J = 8.4 Hz, 2 H), 6.97 (d, J = 8.8 Hz, 2 H), 3.89 (s, 3 H). ^{13}C NMR (100 MHz, $CDCl_3$) δ 194.3, 165.2 (d, J = 251.5 Hz), 163.4, 134.6 (d, J = 3.1 Hz), 132.5 (2 C), 132.4 (d, J = 9.0 Hz, 2 C), 130.2, 115.5 (d, J = 21.6 Hz, 2 C), 113.8 (2 C), 55.7. ^{19}F NMR (367 MHz, $CDCl_3$) δ -107.0. HRMS $C_{14}H_{11}FO_2$ $[M+H]^+$; calculated 231.0816, found: 231.0815.

(4-Methoxyphenyl)(4-(trifluoromethyl)phenyl)methanone (compound 8)

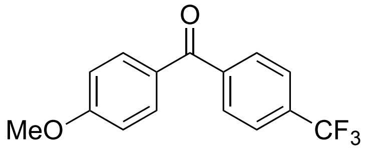

Reaction with 4-iodoanisole (46.8 mg) and 1,1,2,2-tetramethyl-1,2-bis(4-(trifluoromethyl)phenyl)disilane (122.0 mg) using the general procedure at 110 °C followed by flash columm chromatography using pentane/EtOAc (30:1) as eluent resulted in 32 mg (57%) of the title compound as a colorless solid. ^{1}H NMR (400 MHz, $CDCl_3$) δ 7.86-7.80 (m, 4 H), 7.74 (d, J = 8.4 Hz, 2 H), 6.98 (d, J = 8.8 Hz, 2 H), 3.90 (s, 3 H). ^{13}C NMR (100 MHz, $CDCl_3$) δ 194.4, 163.9, 141.7, 133.4 (q, J = 32.4 Hz), 132.8 (2 C), 129.9 (2 C), 129.5, 125.4 (q, J = 3.7 Hz, 2 C), 123.9 (q, J = 270.9 Hz), 114.0 (2 C), 55.7. ^{13}F NMR (367 MHz, $CDCl_3$) δ -62.9. HRMS $C_{15}H_{11}F_3O_2$ $[M+H]^+$; calculated 281.0784, found: 281.0791.

(2,3-Dihydrobenzofuran-5-yl)(3,5-dimethylphenyl)methanone (compound 9)

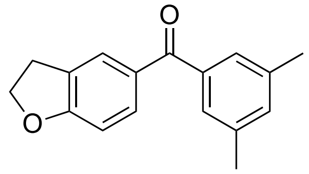

Reaction with methyl 5-iodo-2,3-dihydrobenzofuran (49.2 mg) and 1,2-bis(3,5-dimethylphenyl)-1,1,2,2-tetramethyldisilane (98.0 mg) using the general procedure at 110 °C followed by flash columm chromatography using pentane/EtOAc (100:1) as eluent resulted in

38 mg (78%) of the title compound as a colorless solid. ^{1}H NMR (400 MHz, $CDCl_3$) δ 7.75 (s, 1 H), 7.67–7.60 (m, 1 H), 7.33 (s, 2 H), 7.18 (s, 1 H), 6.81 (d, J = 8.4 Hz, 1 H), 4.67 (t, J = 8.8 Hz, 2 H), 3.26 (t, J = 8.8 Hz, 2 H), 2.37 (s, 6 H). ^{13}C NMR (100 MHz, $CDCl_3$) δ 196.0, 164.0, 138.7, 137.7 (2 C), 133.3, 132.4, 130.7, 127.5, 127.4, 127.3 (2 C), 108.7, 72.1, 29.0, 21.2 (2 C). HRMS $C_{17}H_{16}O_2$ $[M+H]^+$; calculated 253.1223, found: 253.1224.

(4-(Dibenzylamino)phenyl)(3,5-dimethylphenyl)methanone (compound 10)

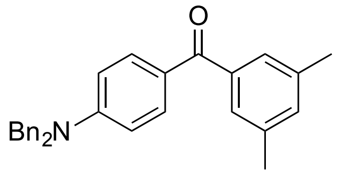

Reaction with *N*,*N*-dibenzyl-4-iodoaniline (79.9 mg) and 1,2-bis(3,5-dimethylphenyl)-1,1,2,2-tetramethyldisilane (98.0 mg) using the general procedure at 110 °C followed by flash columm chromatography using pentane/EtOAc (10:1) as eluent resulted in 61 mg (76%) of the title compound as a pale yellow solid. ^{1}H NMR (400 MHz, $CDCl_3$) δ 7.61 (d, J = 8.8 Hz, 2 H), 7.19 (m, 8 H), 7.09 (d, J = 7.2 Hz, 4 H), 7.00 (s, 1 H), 6.61 (d, J = 8.8 Hz, 2 H), 4.60 (s, 4 H), 2.21 (s, 6 H). ^{13}C NMR (100 MHz, $CDCl_3$) δ 195.4, 152.5, 139.1, 137.6, 137.3 (2 C), 132.9 (2 C), 132.8 (2 C), 128.9 (4 C), 127.3 (2 C), 127.2 (2 C), 126.4 (4 C), 126.0, 111.1 (2 C), 54.0 (2 C), 21.3 (2 C). HRMS $C_{29}H_{27}NO$ $[M+H]^+$; calculated 406.2165, found: 406.2171.

(4-(2,5-Dimethyl-1*H*-pyrrol-1-yl)phenyl)(3,5-dimethylphenyl)methanone (compound 11)

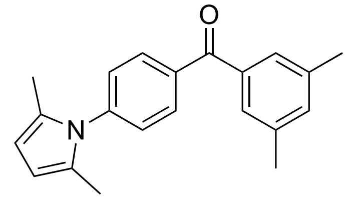

Reaction with 1-(4-iodophenyl)-2,5-dimethyl-1*H*-pyrrole (59.4 mg) and 1,2-bis(3,5-dimethylphenyl)-1,1,2,2-tetramethyldisilane (98.0 mg) using the general procedure at 110 °C followed by flash columm chromatography using pentane/EtOAc (50:1) as eluent resulted in 43 mg (71%) of the title compound as a yellow solid. ^{1}H NMR (400 MHz, $CDCl_3$) δ 7.95 (d, J = 8.0 Hz, 2 H), 7.48 (s, 2 H), 7.36 (d, J = 8.0 Hz, 2 H), 7.29 (s, 1 H), 5.98 (s, 2 H), 2.44 (s, 6 H), 2.13 (s, 6 H). ^{13}C NMR (100 MHz, $CDCl_3$) δ 196.2, 142.5, 138.1 (2 C), 137.4, 136.8, 134.3, 130.9 (2 C), 128.7 (2 C), 127.9 (2 C), 127.7 (2 C), 106.5 (2 C), 21.3 (2 C), 13.2 (2 C). HRMS $C_{21}H_{21}NO$ $[M+H]^+$; calculated 304.1696, found: 304.1695.

(3,5-Dimethylphenyl)(*p*-tolyl)methanone (compound 12)

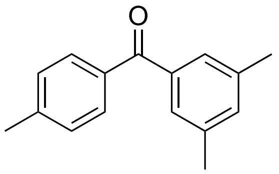

Reaction with 1-iodo-4-methylbenzene (43.6 mg) and 1,2-bis(3,5-dimethylphenyl)-1,1,2,2-tetramethyldisilane (98.0 mg) using the general procedure at 110 °C followed by flash columm chromatography using pentane/EtOAc (100:1) as eluent resulted in 31 mg (70%) of the title compound as a colorless solid. ^{1}H NMR (400 MHz, $CDCl_3$) δ 7.74 (d, J = 8.0 Hz, 2 H), 7.41 (s, 2 H), 7.30 (d, J = 8.0 Hz, 2 H), 7.23 (s, 1 H), 2.47 (s, 3 H), 2.40 (s, 6 H). ^{13}C NMR (100 MHz, $CDCl_3$) δ 196.9, 143.0, 138.0, 137.8 (2 C), 135.2, 133.8, 130.2 (2 C), 128.9 (2 C), 127.7 (2 C), 21.6, 21.2 (2 C). HRMS $C_{16}H_{16}O$ $[M+H]^+$; calculated 225.1274, found: 225.1276.

(4-Methoxyphenyl)(phenyl)methanone (compound 13)

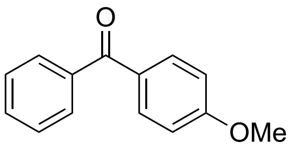

Reaction with iodobenzene (40.8 mg) and 1,2-bis(4-methoxyphenyl)-1,1,2,2-tetramethyldisilane (99.2 mg) using the general procedure at 110 °C followed by flash columm chromatography using pentane/EtOAc (40:1) as eluent resulted in 26 mg (62%) of the title compound as a colorless solid. ^{1}H NMR (400 MHz, $CDCl_3$) δ 7.83 (d, J = 8.8 Hz, 2 H), 7.76 (d, J = 7.2 Hz, 2 H), 7.56 (t, J = 7.2 Hz, 1 H), 7.47 (t, J = 7.2 Hz, 2 H), 6.96 (d, J = 8.8 Hz, 2 H), 3.89 (s, 3 H). ^{13}C NMR (100 MHz, $CDCl_3$) δ 195.5, 163.2, 138.3, 132.5 (2 C), 131.9, 130.1, 129.7 (2 C), 128.2 (2 C), 113.5 (2 C), 55.5. HRMS $C_{14}H_{12}O_2$ $[M+H]^+$; calculated 213.0910, found: 213.0911.

[1,1'-Biphenyl]-4-yl(3,5-dimethylphenyl)methanone (compound 14)

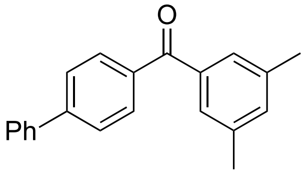

Reaction with 4-iodo-1,1′-biphenyl (56.0 mg) and 1,2-bis(3,5-dimethylphenyl)-1,1,2,2-tetramethyldisilane (98.0 mg) using the general procedure at 110 °C followed by flash columm chromatography using pentane/EtOAc (100:1) as eluent resulted in

40 mg (71%) of the title compound as a colorless solid. ^{1}H NMR (400 MHz, $CDCl_3$) δ 7.89 (d, J = 8.0 Hz, 2 H), 7.71 (d, J = 8.0 Hz, 2 H), 7.66 (d, J = 7.2 Hz, 2 H), 7.49 (t, J = 7.2 Hz, 2 H), 7.42 (m, 3 H), 7.24 (s, 1 H), 2.40 (s, 6 H). ^{13}C NMR (100 MHz, $CDCl_3$) δ 196.7, 145.0, 140.0, 137.9 (2 C), 137.8, 136.5, 134.0, 130.7 (2 C), 128.9 (2 C), 128.1, 127.7 (2 C), 127.3 (2 C), 126.9 (2 C), 21.2 (2 C). HRMS $C_{21}H_{18}O$ $[M+H]^+$; calculated 287.1430, found: 287.1433.

(3,5-Dimethylphenyl)(3,4,5-trimethoxyphenyl)methanone (compound 15)

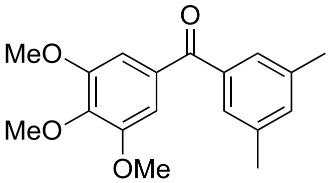

Reaction with 5-iodo-1,2,3-trimethoxybenzene (58.8 mg) and 1,2-bis(3,5-dimethylphenyl)-1,1,2,2-tetramethyldisilane (98.0 mg) using the general procedure at 90 °C followed by flash columm chromatography using pentane/EtOAc (10:1) as eluent resulted in 38 mg (63%) of the title compound as a colorless solid. ^{1}H NMR (400 MHz, $CDCl_3$) δ 7.39 (s, 2 H), 7.22 (s, 1 H), 7.06 (s, 2 H), 3.94 (s, 3 H), 3.87 (s, 6 H), 2.38 (s, 6 H). ^{13}C NMR (100 MHz, $CDCl_3$) δ 196.1, 152.8 (2 C), 141.9, 137.9 (2 C), 137.8, 133.9, 132.9, 127.6 (2 C), 107.7 (2 C), 60.9, 56.3 (2 C), 21.2 (2 C). HRMS $C_{18}H_{20}O_4$ $[M+H]^+$; calculated 301.1434, found: 301.1439.

(4-Methoxyphenyl)(4-(prop-1-en-2-yl)phenyl)methanone (compound 16)

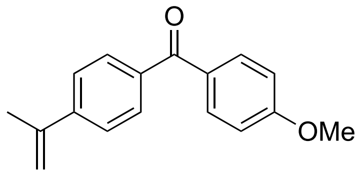

Reaction with 1-iodo-4-(prop-1-en-2-yl)benzene (48.8 mg) and 1,2-bis(4-methoxyphenyl)-1,1,2,2-tetramethyldisilane (99.2 mg) using the general procedure at 110 °C followed by flash columm chromatography using pentane/EtOAc (30:1) as eluent resulted in 27 mg (54%) of the title compound as a colorless solid. ^{1}H NMR (400 MHz, $CDCl_3$) δ 7.83 (d, J = 8.8 Hz, 2 H), 7.74 (d, J = 8.4 Hz, 2 H), 7.56 (d, J = 8.4 Hz, 2 H), 6.97 (d, J = 8.8 Hz, 2 H), 5. 49 (s, 1 H), 5.21 (s, 1 H), 3.89 (s, 3 H), 2.20 (s, 3 H). ^{13}C NMR (100 MHz, $CDCl_3$) δ 195.2, 163.3, 144.9, 142.6, 137.1, 132.6 (2 C), 130.4, 130.0 (2 C), 125.4 (2 C), 114.5, 113.7 (2 C), 55.6, 21.8. HRMS $C_{17}H_{16}O_2$ $[M+H]^+$; calculated 253.1223, found: 253.1226.

(4-Chlorophenyl)(4-methoxyphenyl)methanone (compound 17)

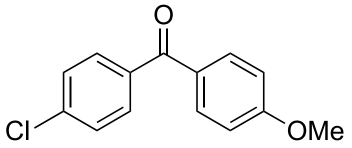

Reaction with 1-chloro-4-iodobenzene (47.7 mg) and 1,2-bis(4-methoxyphenyl)-1,1,2,2-tetramethyldisilane (99.2 mg) using the general procedure at 110 °C followed by flash columm chromatography using pentane/EtOAc (40:1) as eluent resulted in 30 mg (62%) of the title compound as a colorless solid. ^{1}H NMR (400 MHz, $CDCl_3$) δ 7.79 (d, J = 8.4 Hz, 2 H), 7.70 (d, J = 8.4 Hz, 2 H), 7.45 (d, J = 8.4 Hz, 2 H), 6.96 (d, J = 8.4 Hz, 2 H), 3.89 (s, 3 H). ^{13}C NMR (100 MHz, $CDCl_3$) δ 194.2, 163.4, 138.2, 136.5, 132.4 (2 C), 131.1 (2 C), 129.8, 128.5 (2 C), 113.6 (2 C), 55.5. HRMS $C_{14}H_{11}ClO_2$ $[M+H]^+$; calculated 247.0520, found: 247.0520.

4-(4-Methoxybenzoyl)phenyl 4-methylbenzenesulfonate (compound 18)

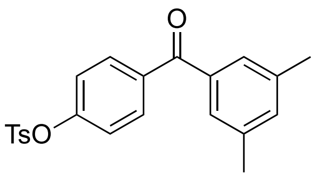

Reaction with 4-iodophenyl 4-methylbenzenesulfonate (74.8 mg) and 1,2-bis(3,5-dimethylphenyl)-1,1,2,2-tetramethyldisilane (98.0 mg) using the general procedure at 100 °C followed by flash columm chromatography using pentane/EtOAc (15:1) as eluent resulted in 40 mg (52%) of the title compound as a colorless solid. ^{1}H NMR (400 MHz, $CDCl_3$) δ 7.75 (dd, J = 2.4, 8.4 Hz, 4 H), 7.38-7.31 (m, 4 H), 7.23 (s, 1 H), 7.11 (d, J = 8.4 Hz, 2 H), 2.46 (s, 3 H), 2.37 (s, 6 H). ^{13}C NMR (100 MHz, $CDCl_3$) δ 195.8, 152.5, 145.9, 138.2 (2 C), 137.3, 136.6, 134.5, 132.3, 131.8 (2 C), 130.0 (2 C), 128.6 (2 C), 127.8 (2 C), 122.3 (2 C), 21.9, 21.4 (2 C). HRMS $C_{22}H_{20}O_4S$ $[M+H]^+$; calculated 381.1155, found: 381.1154.

(3,5-Dimethylphenyl)(3-methoxyphenyl)methanone (compound 19)

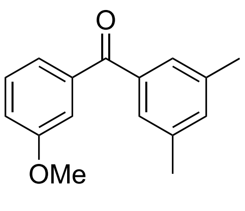

Reaction with 3-iodoanisole (46.8 mg) and 1,2-bis(3,5-dimethylphenyl)-1,1,2,2-tetramethyldisilane (98.0 mg) using the general procedure at 110 °C followed by flash column chromatography using pentane/EtOAc (50:1) as eluent resulted in 31 mg (64%) of the title compound as a colorless solid. HNMR (400 MHz, $CDCl_3$) δ 7.40 (s, 2 H), 7.34 (dt, J = 7.6, 4.4 Hz, 3 H), 7.22 (s, 1 H), 7.15–7.10 (m, 1 H), 3.86 (s, 3 H), 2.37 (s, 6 H). ^{13}C NMR (100 MHz, $CDCl_3$) δ 196.9, 159.5, 139.2, 137.9 (2 C), 137.7, 134.1, 129.1, 127.8 (2 C), 122.8, 118.7, 114.2, 55.4, 21.2 (2 C). HRMS $C_{16}H_{16}O_2$ $[M+H]^+$; calculated 241.1223, found: 241.1224.

Methyl 5-(3,5-dimethylbenzoyl)-2-methoxybenzoate (compound 20)

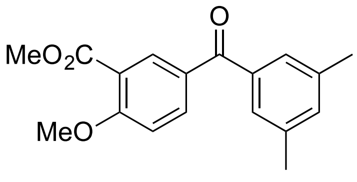

Reaction with methyl 5-iodo-2-methoxybenzoate (58.4 mg) and 1,2-bis(3,5-dimethylphenyl)-1,1,2,2-tetramethyldisilane (98.0 mg) using the general procedure at 100 °C followed by flash columm chromatography using pentane/EtOAc (30:1) as eluent resulted in 49 mg (83%) of the title compound as a colorless solid. ^{1}H NMR (400 MHz, $CDCl_3$) δ 8.28 (d, J = 2.4 Hz, 1 H), 7.96 (dd, J = 8.8, 2.4 Hz, 1 H), 7.33 (s, 2 H), 7.20 (s, 1 H), 7.04 (d, J = 8.8 Hz, 1 H), 3.98 (s, 3 H), 3.88 (s, 3 H), 2.36 (s, 6 H). ^{13}C NMR (100 MHz, $CDCl_3$) δ 195.1, 165.8, 162.1, 138.0 (2 C), 137.7, 135.6, 134.1, 133.9, 129.8, 127.4 (2 C), 119.9, 111.4, 56.3, 52.2, 21.2 (2 C). HRMS $C_{18}H_{18}O_4$ [M+H]$^+$; calculated 299.1278, found: 299.1282.

(4-(*tert*-Butyl)phenyl)(4-nitrophenyl)methanone (compound 21)

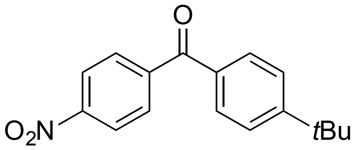

Reaction with 1-iodo-4-nitrobenzene (49.8 mg) and 1,2-bis(4-*tert*-butyl)phenyl-1,1,2,2-tetramethyldisilane (114.8 mg) using the general procedure at 80 °C followed by flash columm chromatography using pentane/EtOAc (40:1) as eluent resulted in 23 mg (40%) of the title compound as a colorless solid. ^{1}H NMR (400 MHz, $CDCl_3$) δ 8.33 (d, J = 8.8 Hz, 2 H), 7.93 (d, J = 8.8 Hz, 2 H), 7.75 (d, J = 8.4 Hz, 2 H), 7.53 (d, J = 8.4 Hz, 2 H), 1.37 (s, 9 H). ^{13}C NMR (100 MHz, $CDCl_3$) δ 194.6, 157.6, 149.8, 143.4, 133.7, 130.7 (2 C), 130.3 (2 C), 125.8 (2 C), 123.6 (2 C), 35.4, 31.2 (3 C). HRMS $C_{17}H_{17}NO_3$ [M+H]$^+$; calculated 284.1281, found: 284.1279.

4-Benzoylbenzonitrile (compound 22)

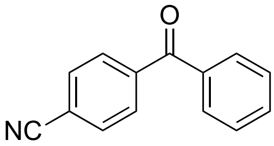

Reaction with 4-iodobenzonitrile (45.8 mg) and 1,1,2,2-tetramethyl-1,2-diphenyldisilane (81.2 mg) using the general procedure at 80 °C followed by flash columm chromatography using pentane/EtOAc (10:1) as eluent resulted in 28 mg (67%) of the title compound as a colorless solid. ^{1}H NMR (400 MHz, $CDCl_3$) δ 7.87 (d, J = 8.0 Hz, 2 H), 7.79 (dd, J = 8.0, 2.0 Hz, 4 H), 7.64 (t, J = 7.6 Hz, 1 H), 7.51 (t, J = 7.6 Hz, 2 H). ^{13}C NMR (100 MHz, $CDCl_3$) δ 195.0, 141.2, 136.3, 133.3, 132.1 (2 C), 130.2 (2 C), 130.0 (2 C), 128.6 (2 C), 118.0, 115.6. HRMS $C_{14}H_9NO$ $[M+H]^+$; calculated 208.0757, found: 208.0759.

1-(4-Benzoylphenyl)ethan-1-one (compound 23)

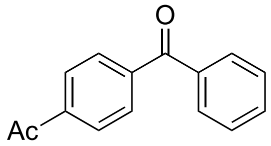

Reaction with 1-(4-iodophenyl)ethan-1-one (49.2 mg) and 1,1,2,2-tetramethyl-1,2-diphenyldisilane (81.2 mg) using the general procedure at 80 °C followed by flash columm chromatography using pentane/EtOAc (10:1) as eluent resulted in 28 mg (64%) of the title compound as a colorless solid. ^{1}H NMR (400 MHz, $CDCl_3$) δ 8.05 (d, J = 8.0 Hz, 2 H), 7.86 (d, J = 8.0 Hz, 2 H), 7.80 (d, J = 6.8 Hz, 2 H), 7.61 (t, J = 7.6 Hz, 1 H), 7.49 (t, J = 7.6 Hz, 2 H), 2.66 (s, 3 H). ^{13}C NMR (100 MHz, $CDCl_3$) δ 197.5, 195.9, 141.3, 139.5, 136.9, 133.0, 130.1 (2 C), 130.0 (2 C), 128.4 (2 C), 128.1 (2 C), 26.9. HRMS $C_{15}H_{12}O_2$ $[M+H]^+$; calculated 225.0910, found: 225.0911.

1-(3-Benzoylphenyl)ethan-1-one (compound 24)

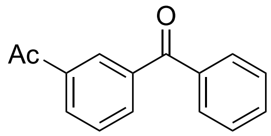

Reaction with 1-(3-iodophenyl)ethan-1-one (49.2 mg) and 1,1,2,2-tetramethyl-1,2-diphenyldisilane (81.2 mg) using the general procedure at 80 °C followed by flash columm chromatography using

pentane/EtOAc (10:1) as eluent resulted in 34 mg (75%) of the title compound as a colorless solid. ^{1}H NMR (400 MHz, $CDCl_3$) δ 8.36 (s, 1 H), 8.18 (d, J = 8.0 Hz, 1 H), 7.98 (d, J = 7.6 Hz, 1 H), 7.80 (d, J = 7.2 Hz, 2 H), 7.63-7.58 (m, 2 H), 7.50 (t, J = 7.6 Hz, 2 H), 2.64 (s, 3 H). ^{13}C NMR (100 MHz, $CDCl_3$) δ 197.3, 195.9, 138.1, 137.2, 137.0, 134.3, 132.9, 131.8, 130.0 (2 C), 129.7, 128.8, 128.5 (2 C), 26.8. HRMS $C_{15}H_{12}O_2$ $[M+H]^+$; calculated 225.0910, found: 225.0910.

Naphthalen-1-yl(thiophen-3-yl)methanone (compound 25)

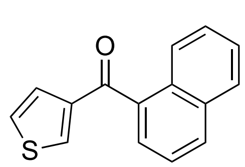

Reaction with 3-iodothiophene (42.0 mg) and 1,1,2,2-tetramethyl-1,2-di(naphthalen-1-yl)disilane (111.2 mg) using the general procedure at 80 °C followed by flash columm chromatography using pentane/EtOAc (10:1) as eluent resulted in 35 mg (74%) of the title compound as a colorless solid. ^{1}H NMR (400 MHz, $CDCl_3$) δ 8.19-8.12 (m, 1 H), 8.00 (d, J = 8.0 Hz, 1 H), 7.96-7.89 (m, 1 H), 7.86-7.82 (m, 1 H), 7.71-7.63 (m, 2 H), 7.57-7.48 (m, 3 H), 7.38-7.36 (m, 1 H). ^{13}C NMR (100 MHz, $CDCl_3$) δ 191.4, 143.3, 137.1, 135.5, 133.9, 131.4, 130.7, 128.5, 128.4, 127.4, 127.2, 126.6, 126.5, 125.7, 124.4. HRMS $C_{15}H_{10}OS$ $[M+H]^+$; calculated 239.0525, found: 239.0524.

(4-(*tert*-Butyl)phenyl)(quinolin-6-yl)methanone (compound 26)

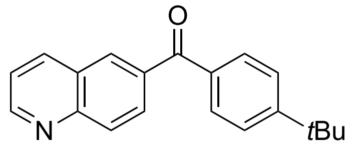

Reaction with 6-iodoquinoline (51.0 mg) and 1,2-bis(4-*tert*-butyl)phenyl-1,1,2,2-tetramethyldisilane (114.8 mg) using the general procedure at 80 °C followed by flash columm chromatography using pentane/EtOAc (4:1) as eluent resulted in 41 mg (70%) of the title compound as a colorless solid. ^{1}H NMR (400 MHz, $CDCl_3$) δ 9.02 (dd, J = 1.6, 4.0 Hz, 1 H), 8.30-8.12 (m, 4 H), 7.81 (d, J = 8.4 Hz, 2 H), 7.53 (d, J = 8.4 Hz, 2 H), 7.48 (dd, J = 4.4, 8.4 Hz, 1 H), 1.38 (s, 9 H). ^{13}C NMR (100 MHz, $CDCl_3$) δ 195.8, 156.6, 152.5, 149.8, 137.4, 135.9, 134.8, 131.2, 130.3 (2 C), 129.9, 129.7, 127.4, 125.5 (2 C), 122.0, 35.3, 31.3 (3 C). HRMS $C_{20}H_{19}NO$ $[M+H]^+$; calculated 290.1539, found: 290.1541.

(1-Methyl-1*H*-pyrazol-4-yl)(phenyl)methanone (compound 27)

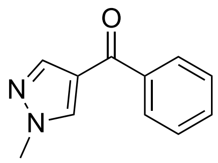

Reaction with 4-iodo-1-methyl-1*H*-pyrazole (41.6 mg) and 1,1,2,2-tetramethyl-1,2-diphenyldisilane (81.2 mg) using the general procedure at 80 °C followed by flash columm chromatography using pentane/EtOAc (2:1) as eluent resulted in 19 mg (50%) of the title compound as a colorless solid. ^{1}H NMR (400 MHz, $CDCl_3$) δ 7.92 (d, J = 3.2 Hz, 2 H), 7.83 (d, J = 7.2 Hz, 2 H), 7.56 (t, J = 7.2 Hz, 1 H), 7.48 (t, J = 7.2 Hz, 2 H), 3.96 (s, 3 H). ^{13}C NMR (100 MHz, $CDCl_3$) δ 188.8, 141.8, 139.1, 134.2, 132.2, 128.7 (2 C), 128.5 (2 C), 122.8, 39.3. HRMS $C_{11}H_{10}N_2O$ $[M+H]^+$; calculated 187.0866, found: 187.0866.

(3,5-Dimethylphenyl)(pyridin-3-yl)methanone (compound 28)

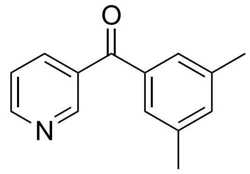

Reaction with 3-iodopyridine (41.0 mg) and 1,2-bis(3,5-dimethylphenyl)-1,1,2,2-tetramethyldisilane (98.0 mg) using the general procedure at 90 °C followed by flash columm chromatography using pentane/EtOAc (5:1) as eluent resulted in 29 mg (70%) of the title compound as a colorless solid. ^{1}H NMR (400 MHz, $CDCl_3$) δ 8.91 (s, 1 H), 8.73 (s, 1 H), 8.04 (d, J = 7.6 Hz, 1 H), 7.38 (dd, J = 7.2, 4.8 Hz, 1 H), 7.33 (s, 2 H), 7.19 (s, 1 H), 2.31 (s, 6 H). ^{13}C NMR (100 MHz, $CDCl_3$) δ 195.2, 152.6, 150.8, 138.3 (2 C), 137.2, 136.8, 134.8, 133.5, 127.7 (2 C), 123.4, 21.2 (2 C). HRMS $C_{14}H_{13}NO$ $[M+H]^+$; calculated 212.1070, found: 212.1073.

(1-Methyl-1*H*-indol-5-yl)(3,4,5-trimethoxyphenyl)methanone (compound 29)

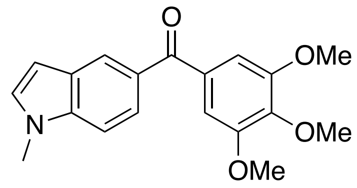

Reaction with 5-iodo-1-methyl-1*H*-indole (51.4 mg) and 1,1,2,2-tetramethyl-1,2-bis(3,4,5-trimethoxyphenyl)disilane (135.2 mg) using the general procedure at 90 °C followed by flash columm chromatography using pentane/EtOAc (4:1) as

eluent resulted in 50 mg (77%) of the title compound as a colorless solid. ^{1}H NMR (400 MHz, $CDCl_3$) δ 8.14 (s, 1 H), 7.80 (d, J = 8.4 Hz, 1 H), 7.39 (d, J = 8.4 Hz, 1 H), 7.15 (s, 1 H), 7.08 (s, 2 H), 6.60 (s, 1 H), 3.94 (s, 3 H), 3.87 (s, 6 H), 3.85 (s, 3 H). ^{13}C NMR (100 MHz, $CDCl_3$) δ 196.5, 152.8 (2 C), 141.4, 139.0, 134.3, 130.5, 129.4, 127.8, 125.2, 123.9, 109.1, 107.7 (2 C), 103.0, 61.1, 56.4 (2 C), 33.2. HRMS $C_{19}H_{19}NO_4$ $[M+H]^+$; calculated 326.1387, found: 326.1387.

[^{13}C] (1-Methyl-1*H*-indol-5-yl)(3,4,5-trimethoxyphenyl)methanone (compound 30)

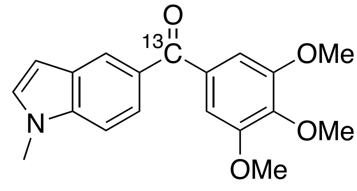

Reaction with 5-iodo-1-methyl-1*H*-indole (51.4 mg) and 1,1,2,2-tetramethyl-1,2-bis(3,4,5-trimethoxyphenyl)disilane (135.2 mg) using the general procedure for ^{13}C-labeling at 90 °C followed by flash columm chromatography using pentane/EtOAc (4:1) as eluent resulted in 52 mg (80%) of the title compound as a colorless solid. ^{1}H NMR (400 MHz, $CDCl_3$) δ 8.13 (d, J = 2.4 Hz, 1 H), 7.79 (d, J = 6.8 Hz, 1 H), 7.39 (d, J = 8.4 Hz, 1 H), 7.14 (d, J = 2.8 Hz, 1 H), 7.08 (d, J = 4.0 Hz, 2 H), 6.59 (d, J = 2.8 Hz, 1 H), 3.94 (s, 3 H), 3.86 (s, 6 H), 3.84 (s, 3 H). ^{13}C NMR (100 MHz, $CDCl_3$) δ 196.4 (^{13}C-enriched), 152.8 (d, J = 5.6 Hz, 2 C), 141.3, 139.0, 134.2 (d, J = 54.6 Hz) 130.5, 129.3 (d, J = 56.4 Hz), 127.8 (d, J = 4.7 Hz), 125.2 (d, J = 3.4 Hz), 123.9 (d, J =3.0 Hz), 109.1 (d, J = 4.4 Hz), 107.7 (d, J = 3.0 Hz, 2 C), 103.0, 61.0, 56.4 (2 C), 33.1. HRMS $C_{18}{}^{13}CH_{19}NO_4$ $[M+H]^+$; calculated 327.1420, found: 327.1422.

Isopropyl 2-(4-(4-chlorobenzoyl)phenoxy)-2-methylpropanoate (compound 31)

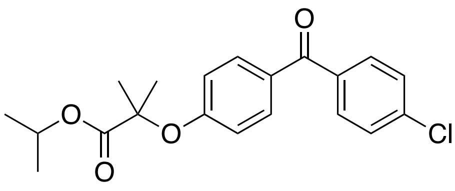

Reaction with isopropyl 2-(4-iodophenoxy)-2-methylpropanoate (69.6 mg) and 1,2-bis(4-chlorophenyl)-1,1,2,2-tetramethyldisilane (101.8 mg) using the general procedure at 90 °C followed by flash

columm chromatography using pentane/EtOAc (20:1) as eluent resulted in 38 mg (52%) of the title compound as a colorless solid. ^{1}H NMR (400 MHz, $CDCl_3$) δ 7.77-7.66 (m, 4 H), 7.44 (d, J = 8.4 Hz, 2 H), 6.86 (d, J = 8.8 Hz, 2 H), 5.09 (sep, J = 6.4 Hz, 1 H), 1.66 (s, 6 H), 1.20 (d, J = 6.4 Hz, 6 H). ^{13}C NMR (100 MHz, $CDCl_3$) δ 194.4, 173.2, 159.9, 138.5, 136.6, 132.1 (2 C), 131.3 (2 C), 130.4, 128.7 (2 C), 117.4 (2 C), 79.6, 69.5, 25.5 (2 C), 21.7 (2 C). HRMS $C_{20}H_{21}ClO_4$ $[M+H]^+$; calculated 361.1201, found: 361.1203.

[^{13}C] Isopropyl 2-(4-(4-chlorobenzoyl)phenoxy)-2-methylpropanoate (compound 32)

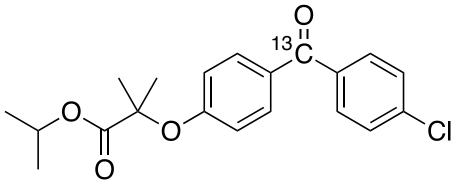

Reaction with isopropyl 2-(4-iodophenoxy)-2-methylpropanoate (69.6 mg) and 1,2-bis(4-chlorophenyl)-1,1,2,2-tetramethyldisilane (101.8 mg) using the general procedure for ^{13}C-labeling at 90 °C followed by flash columm chromatography using pentane/EtOAc (20:1) as eluent resulted in 40 mg (56%) of the title compound as a colorless solid. ^{1}H NMR (400 MHz, $CDCl_3$) δ 7.73 (dd, J = 4.0, 8.8 Hz, 2 H), 7.70 (dd, J = 3.6, 8.8 Hz, 2 H), 7.44 (d, J = 8.4 Hz, 2 H), 6.86 (d, J = 8.8 Hz, 2 H), 5.09 (sep, J = 6.4 Hz, 1 H), 1.66 (s, 6 H), 1.20 (d, J = 6.4 Hz, 6 H). ^{13}C NMR (100 MHz, $CDCl_3$) δ 194.4 (^{13}C-enriched), 173.2, 159.9 (d, J = 1.1 Hz), 138.5 (d, J = 1.2 Hz), 136.6 (d, J = 55.0 Hz), 132.1 (d, J = 3.1 Hz, 2 C), 131.3 (d, J = 2.9 Hz, 2 C), 130.4 (d, J = 57.0 Hz), 128.7 (d, J = 4.2 Hz, 2 C), 117.4 (d, J = 4.3 Hz, 2 C), 79.6, 69.5, 25.5 (2 C), 21.7 (2 C). HRMS $C_{19}{}^{13}CH_{21}ClO_4$ $[M+H]^+$; calculated 362.1235, found: 362.1239.

2-(3-Benzoylphenyl)propanenitrile (compound 33a)

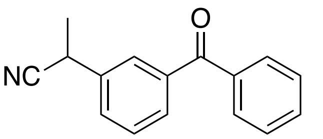

Reaction with 2-(3-iodophenyl)propanenitrile (51.4 mg) and 1,1,2,2-tetramethyldisilane-1,2-diphenyldisilane (81.2 mg) using the general procedure at 80 °C followed by flash columm chromatography using pentane/EtOAc (20:1) as eluent resulted in 31 mg (65%) of the title compound as a colorless solid. ^{1}H NMR (400 MHz, $CDCl_3$) δ 7.81 (s, 1 H), 7.79 (s, 2 H), 7.74 (d, J = 7.6 Hz, 1 H), 7.62 (t, J = 7.2 Hz, 2 H), 7.51 (q, J = 7.2 Hz, 3 H), 3.99 (q, J = 7.2 Hz, 1 H), 1.69 (d, J = 7.6 Hz, 3 H). ^{13}C NMR (100 MHz, $CDCl_3$) δ 196.1, 138.7, 137.7, 137.3, 132.9, 130.7, 130.2 (2 C), 130.0, 129.3, 128.6 (2 C), 128.3, 121.3, 31.3, 21.5. HRMS $C_{16}H_{13}NO$ $[M+H]^+$; calculated 236.1070, found: 236.1070.

[^{13}C] 2-(3-Benzoylphenyl)propanenitrile (compound 34a)

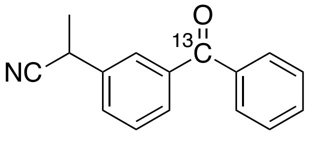

Reaction with 2-(3-iodophenyl)propanenitrile (51.4 mg) and 1,1,2,2-tetramethyldisilane-1,2-diphenyldisilane (81.2 mg) using the general procedure for ^{13}C-labeling at 80 °C followed by flash columm chromatography using pentane/EtOAc (20:1) as eluent resulted in 30 mg (63%) of the title compound as a colorless solid. ^{1}H NMR (400 MHz, $CDCl_3$) δ 7.83-7.77 (m, 3 H), 7.76-7.72 (m, 1 H), 7.62 (t, J = 7.6 Hz, 2 H), 7.51 (q, J = 7.2 Hz, 3 H), 3.99 (q, J = 7.2 Hz, 1 H), 1.69 (d, J = 7.2 Hz, 3 H). ^{13}C NMR (100 MHz, $CDCl_3$) δ 196.1 (^{13}C-enriched), 138.7 (d, J = 54.2 Hz), 137.7 (d, J = 4.0 Hz), 137.3 (d, J = 54.9 Hz), 132.9, 130.7, 130.2 (d, J = 2.8 Hz, 2 C), 130.0 (d, J = 2.9 Hz), 129.3 (d, J = 4.1 Hz), 128.6 (d, J = 4.1 Hz, 2 C), 128.3 (d, J = 2.7 Hz), 121.2, 31.3, 21.5. HRMS $C_{15}{}^{13}CH_{13}NO$ $[M+H]^+$; calculated 237.1103, found: 237.1106.

2-(3-Benzoylphenyl)propanoic acid (compound 33)

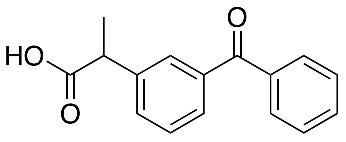

33a (30.6 mg, 0.13 mmol) was transferred to a COtube. AcOH (1 mL), conc. H_2SO_4 (1 mL) and water (1 mL) was added and the COtube was sealed and heated to 100 °C for 18 hours. The mixture was cooled to room temperature and diluted with water and extracted with EtOAc (3x). The combined organic phases were washed with water (3x) and brine (1x), dried over $MgSO_4$, filtered and concentrated *in vacuo* to give the title compound as colorless oil (32 mg, 96%). ^{1}H NMR (400 MHz, CD_3CN) δ 9.17 (bs, 1 H), 7.76 (d, J = 6.8 Hz, 2 H), 7.71 (s, 1 H), 7.69-7.62 (m, 2 H), 7.59 (d, J = 7.6 Hz, 1 H), 7.53 (t, J = 7.6 Hz, 2 H), 7.48 (d, J = 7.6 Hz, 1 H), 3.84 (q, J = 7.2 Hz, 1 H), 1.46 (d, J = 7.2 Hz, 3 H). ^{13}C NMR (100 MHz, CD_3CN) δ 197.0, 175.6, 142.4, 138.8, 138.4, 133.5, 132.6, 130.7 (2 C), 129.8, 129.6 (2 C), 129.4 (2 C), 45.5, 18.8. HRMS $C_{16}H_{14}O_3$ $[M-H]^-$; calculated 253.0870, found: 253.0881.

[^{13}C] 2-(3-Benzoylphenyl)propanoic acid (compound 34)

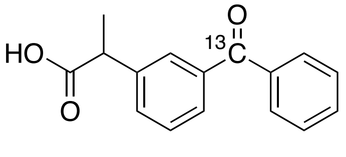

34a (30 mg, 0.13 mmol) was transferred to a COtube. AcOH (1 mL), conc. H_2SO_4 (1 mL) and water (1 mL) was added and the COtube was sealed and heated to 100 °C for 18 hours. The mixture was cooled to room temperature and diluted with water and extracted with EtOAc (3x). The combined organic phases were washed with water (3x) and brine (1x), dried over $MgSO_4$, filtered and concentrated *in vacuo* to give the title compound as colorless oil (32 mg,

95%). ^{1}H NMR (400 MHz, CD_3CN) δ 7.79-7.74 (m, 2 H), 7.72-7.70 (m, 1 H), 7.68-7.63 (m, 2 H), 7.62 (d, J = 7.6 Hz, 1 H), 7.53 (t, J = 7.6 Hz, 2 H), 7.48 (d, J = 7.6 Hz, 1 H), 3.84 (q, J = 7.2 Hz, 1 H), 1.46 (d, J = 7.2 Hz, 3 H). ^{13}C NMR (100 MHz, CD_3CN) δ 197.0 (^{13}C-enriched), 175.6, 142.4 (d, J = 3.8 Hz), 139.9 (d, J = 33.3 Hz), 138.3 (d, J = 33.6 Hz), 133.5, 132.6, 130.7 (d, J = 2.6 Hz, 2 C), 129.8 (d, J = 2.7 Hz), 129.7-129.6 (m, 2 C), 129.4 (d, J = 3.9 Hz, 2 C), 45.5, 18.8. HRMS $C_{15}{}^{13}CH_{14}O_3$ $[M-H]^-$; calculated 254.0904, found: 254.0908.

1,4-Phenylenebis((3,4,5-trimethoxyphenyl)methanone) (compound 35)

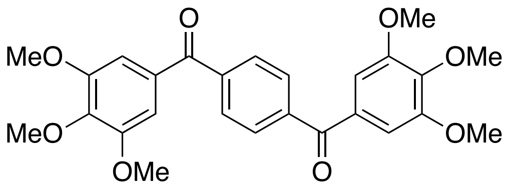

Reaction with 1,4-diiodobenzene (0.1 mmol, 33.0 mg) and 1,1,2,2-tetramethyl-1,2-bis(3,4,5-trimethoxyphenyl)disilane (135.2 mg) using the general procedure at 80 °C followed by flash columm chromatography using pentane/EtOAc (20% → 40%) as eluent resulted in 34 mg (73%) of the title compound as a colorless solid. ^{1}H NMR (400 MHz, $CDCl_3$) δ 7.89 (s, 4 H), 7.09 (s, 4 H), 3.95 (s, 6 H), 3.88 (s, 12 H). ^{13}C NMR (100 MHz, $CDCl_3$) δ 195.2 (2 C), 153.1 (4 C), 142.8 (2 C), 141.0 (2 C), 131.9 (2 C), 129.6 (4 C), 108.0 (4 C), 61.2 (2 C), 56.5 (4 C). HRMS $C_{26}H_{26}O_8$ $[M+H]^+$; calculated 467.1700, found: 467.1714.

4-(3,5-Dimethylbenzoyl)benzonitrile (compound 43)

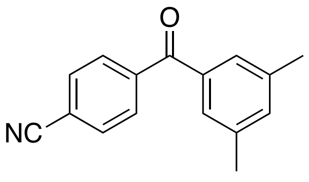

Isolated following the procedure described for evaluating the halide effect by flash columm chromatography using pentane/EtOAc (20:1) as eluent as a colorless solid. ^{1}H NMR (400 MHz, $CDCl_3$) δ 7.86 (d, J = 8.4 Hz, 2 H), 7.79 (d, J = 8.4 Hz, 2 H), 7.37 (s, 2 H), 7.27 (s, 1H), 2.38 (s, 6 H). ^{13}C NMR (100 MHz, $CDCl_3$) δ 195.6, 141.7, 138.5, 136.6, 135.1 (2 C), 132.2 (2 C), 130.3 (2 C), 127.9 (2 C), 118.2, 115.6, 21.4 (2 C). HRMS $C_{16}H_{13}NO$ $[M+H]^+$; calculated 236.1070, found: 236.1077.
